# Heteroaryl derivatives of suvorexant as OX1R selective PET ligand candidates: Cu-mediated ^18^F-fluorination of boroxines, in vitro and initial in vivo evaluation

**DOI:** 10.1186/s13550-024-01141-2

**Published:** 2024-09-04

**Authors:** Kim-Viktoria Bolik, Jan Hellmann, Simone Maschauer, Eduard Neu, Jürgen Einsiedel, Patrick Riss, Nora Vogg, Jörg König, Martin F. Fromm, Harald Hübner, Peter Gmeiner, Olaf Prante

**Affiliations:** 1 Department of Nuclear Medicine, Molecular Imaging and Radiochemistry, Friedrich-Alexander-Universität Erlangen-Nürnberg (FAU), Kussmaulallee 10/12, 91054 Erlangen, Germany; 2 Department of Chemistry and Pharmacy, Medicinal Chemistry, Friedrich-Alexander-Universität Erlangen-Nürnberg (FAU), Nikolaus-Fiebiger-Str. 10, 91058 Erlangen, Germany; 3 Department of Chemistry, Johannes Gutenberg-Universität (JGU), Fritz Strassmann Weg 2, 55128 Mainz, Germany; 4 Institute of Experimental and Clinical Pharmacology and Toxicology, Friedrich-Alexander-Universität Erlangen-Nürnberg (FAU), Erlangen, Germany; 5 FAU NeW – Research Center New Bioactive Compounds, Friedrich-Alexander-Universität Erlangen-Nürnberg (FAU), Nikolaus-Fiebiger-Str. 10, 91058 Erlangen, Germany

**Keywords:** Orexin receptor, Suvorexant, F-18, Cu-mediated ^18^F-fluorination, PET

## Abstract

**Background:**

The orexin receptor (OXR) plays a role in drug addiction and is aberrantly expressed in colorectal tumors. Subtype-selective OXR PET ligands suitable for in vivo use have not yet been reported. This work reports the development of ^18^F-labeled OXR PET ligand candidates derived from the OXR antagonist suvorexant and the OX1R-selective antagonist JH112.

**Results:**

Computational analysis predicted that fluorine substitution (1e) and introduction of the fluorobenzothiazole scaffold (1f) would be suitable for maintaining high OX1R affinity. After multi-step synthesis of 1a–1f, in vitro OXR binding studies confirmed the molecular dynamics calculations and revealed single-digit nanomolar OX1R affinities for 1a–f, ranging from 0.69 to 2.5 nM. The benzothiazole 1f showed high OX1R affinity (K_i_ = 0.69 nM), along with 77-fold subtype selectivity over OX2R. Cu-mediated ^18^F-fluorination of boroxine precursors allowed for a shortened reaction time of 5 min to provide the non-selective OXR ligand [^18^F]1c and its selective OX1R congener [^18^F]1f in activity yields of 14% and 22%, respectively, within a total synthesis time of 52–76 min. [^18^F]1c and [^18^F]1f were stable in plasma and serum in vitro, with logD_7.4_ of 2.28 ([^18^F]1c) and 2.37 ([^18^F]1f), and high plasma protein binding of 66% and 77%, respectively. Dynamic PET imaging in rats showed similar brain uptake of [^18^F]1c (0.17%ID/g) and [^18^F]1f (0.15%ID/g). However, preinjection of suvorexant did not significantly block [^18^F]1c or [^18^F]1f uptake in the rat brain. Pretreatment with cyclosporine A to study the role of P-glycoprotein (P-gp) in limiting brain accumulation moderately increased brain uptake of [^18^F]1c and [^18^F]1f. Accordingly, in vitro experiments demonstrated that the P-gp inhibitor zosuquidar only moderately inhibited polarized, basal to apical transport of 1c (p < 0.05) and had no effect on the transport of 1f, indicating that P-gp does not play a relevant role in brain accumulation of [^18^F]1c and [^18^F]1f in vivo.

**Conclusions:**

The in vitro and in vivo results of [^18^F]1c and [^18^F]1f provide a solid basis for further development of suitable OXR PET ligands for brain imaging.

**Supplementary Information:**

The online version contains supplementary material available at 10.1186/s13550-024-01141-2.

## Introduction

Orexin receptors (OXRs) are class A G-protein coupled receptors (GPCRs). The importance of the OXR system in various physiological functions and its role in neurological disorders and cancer have been the subject of numerous studies [1, 2]. The endogenous OXR ligands, OX-A and OX-B, which are almost exclusively expressed in the hypothalamus, bind to OX1R and OX2R with different selectivities, such that OX-A is non-selective, whereas OX-B shows preferential binding to OX2R. Each OXR subtype showed distinct expression patterns in the central nervous system. For example, the prefrontal cortex showed predominant expression of the OX1R, whereas the nucleus accumbens possessed only the OX2R [3]. While the OX2R has been reported to modulate the sleep–wake rhythm and OX2R agonism increased wakefulness in narcoleptic mice and human [4, 5], the OX1R has a major impact on emotional behavior and OX1R antagonism influenced cocaine-seeking behavior in mice [6]. Interestingly, OXR expression has been demonstrated in human adrenals [7], where they stimulate glucocorticoid secretion. In adrenocortical tumors, OXR are upregulated and orexins stimulate tumor proliferation, indicating an autocrine-paracrine mechanism for the regulation of adrenal tumor growth [8]. More recent work has shown, that various tumor cell lines, including colorectal, pancreatic and liver cancer cell lines, express OX1R [9–12] and tumor growth of pancreatic AsPC-1 tumor xenografts in nude mice was inhibited by treatment with OX-A [13]. Therefore, the OX1R represents an interesting target for diagnostic and therapeutic approaches related to brain disorders and cancer.

**Fig. 1:**
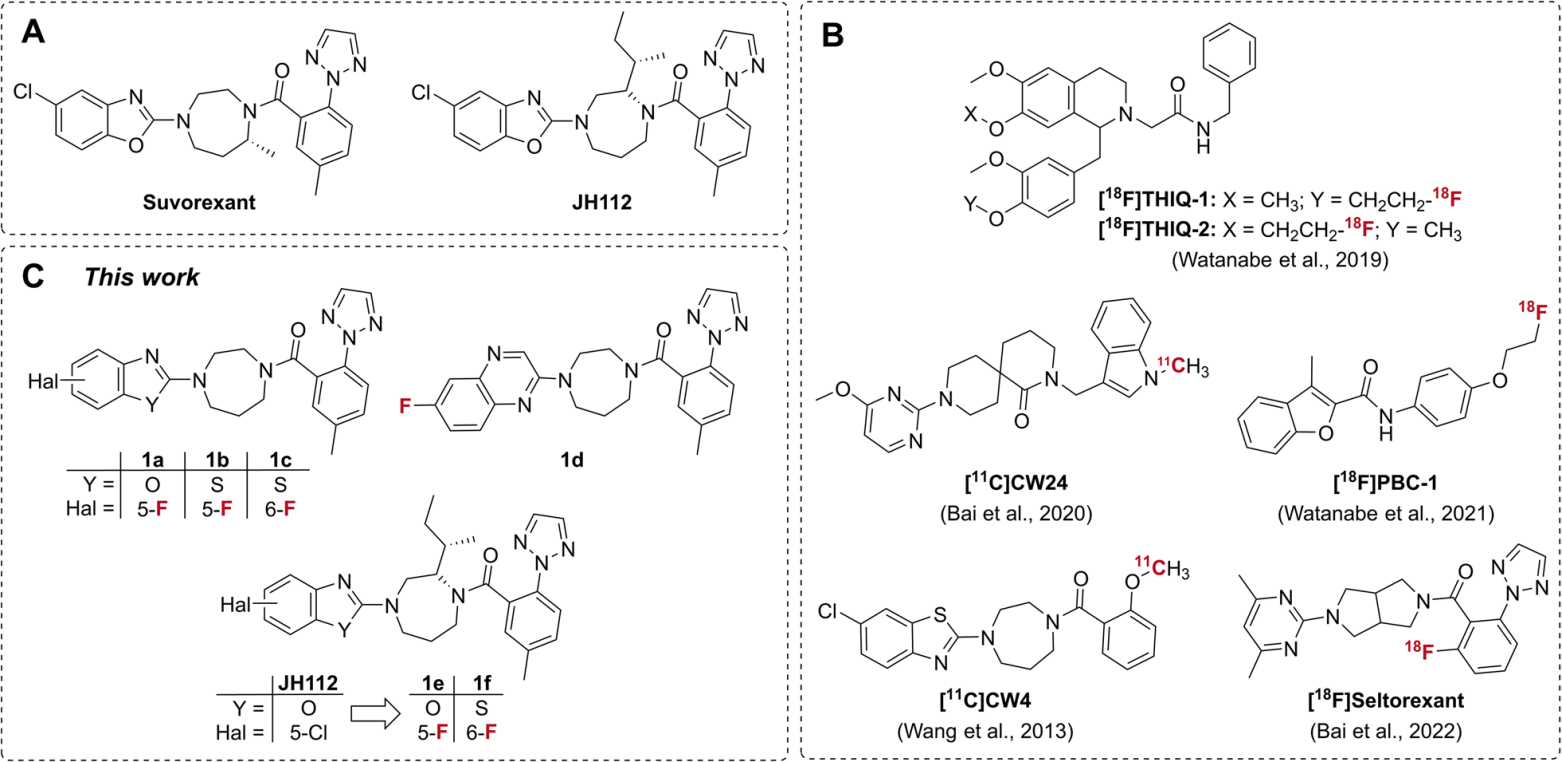
Overview of OXR ligands. **A** Non-subtype-selective OXR ligand suvorexant compared to the OX1R-selective ligand **JH112** [22]. **B** Examples of subtype-selective PET ligand candidates investigated so far [14, 15, 19–21]. **C** Fluorine-substituted derivatives 1a–f studied in this work

Among orexin receptor ligands, the unselective OXR antagonist suvorexant (Fig. 1A) is the only FDA-approved drug used for the treatment of insomnia. However, the exact role of the OXR subtypes in the brain and in tumors is not well understood and requires further elucidation. Molecular imaging with positron emission tomography (PET) applying subtype-selective radioligands could be a very valuable and sensitive tool to achieve this goal. However, previous attempts to develop radioligands suitable for in vivo PET imaging of OX1R or OX2R have been unsuccessful. Most of these efforts addressed OX2R-selective PET ligand candidates (Fig. 1B; [^11^C]CW4 [14], [^18^F]seltorexant [15], and others [16–18]), however, these studies reported low brain uptake, interaction with efflux transporters, or significant lack of specific binding in vivo. The OX1R-selective PET ligand candidates investigated so far faced the same challenges (Fig. 1B), such that [^18^F]THIQ derivatives showed low brain uptake due to poor pharmacokinetics [19], and [^18^F]PBC-1 showed in vivo instability [20]. [^11^C]CW24 with only moderate OX1R affinity showed improved brain uptake but relatively high levels of non-specific binding in the monkey brain at all time points after injection [21].

Starting from the non-subtype-selective lead suvorexant, we successfully developed the OX1R-subtype-selective antagonist **JH112** (Fig. 1A) through the targeted application of docking methods, crystallography, medicinal chemistry, and in vitro pharmacology [22]. **JH112** showed subnanomolar binding affinity to the OX1R (K_i_ = 0.72 nM), while the affinity for the OX2R was 75-fold lower, making **JH112** an excellent lead for the development of OX1R-selective PET ligands.

In the present work, we investigated the fluoro-for-chloro substitution in position 5 of the benzoxazole ring of **JH112** (Fig. 1C; compound **1e**) and the influence of alternative heteroarenes on OX1R affinity and subtype selectivity (Fig. 1C; compound **1f** and heteroarene derivatives **1a–d**), taking into account the accessibility of ^18^F-labeled derivatives by copper-mediated aromatic ^18^F-fluorination. We identified the 6-fluorobenzothiazole derivative **1f** as a ligand with subnanomolar OX1R affinity and retained OX1R selectivity, whose ^18^F-labeled analog was investigated in vitro and in preliminary in vivo experiments.

## Results

### Computational analysis

**Fig. 2:**
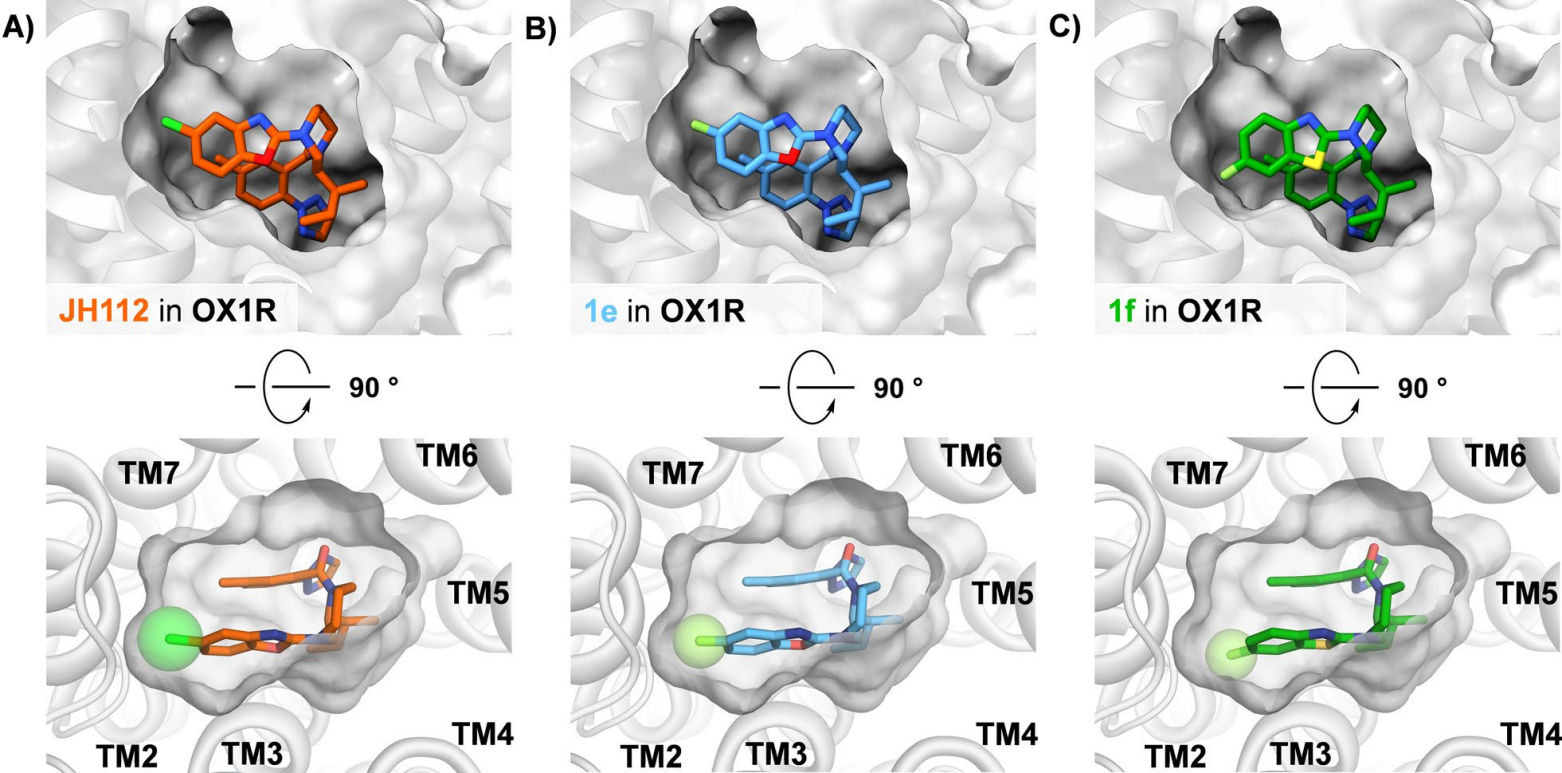
Docking of **JH112** (orange), **1e** (blue), and **1f** (green) into the suvorexant-bound OX1R structure. **A** Docking of **JH112** into the suvorexant-bound OX1R structure (PDB: 6TO7; [24]) yielded a binding mode highly similar to that observed with the **JH112**-bound OX1R structure (PDB: 6V9S; [22]), thereby validating our docking protocol. **B** and **C** illustrate that the 5-fluorobenzoxazole and 6-fluorobenzothiazole moieties of **1e** and **1f** are well accommodated within the binding pocket

To assess the affinity of potential PET ligands for the OX1R, we conducted docking studies and free energy perturbation (FEP) calculations. We first examined compound **1e**, which features a fluorine substituent in place of the original chlorine substituent of **JH112**. Considering the predictable low radiochemical yield (RCY) for Cu-mediated aromatic ^18^F-fluorination of benzoxazoles [23], we also evaluated benzothiazole **1f**. This decision was based on the greater accessibility of benzothiazole BPin ester precursors for ^18^F-fluorination via the same Cu-mediated approach. **JH112**, the 5-fluorobenzoxazole **1e** and the 6-fluorobenzothiazole **1f** were docked into the structure of the OX1R receptor bound with suvorexant (PDB: 6TO7; [24]). Our docking utilized core constraints referencing suvorexant with a tolerance of 2 Å, following our previously established protocol [25]. The results indicated that the respective fluoro substitutions at the 5-position of the benzoxazole and the 6-position of the benzothiazole were well accommodated within the binding pocket (Fig. 2).

Because docking was conducted with core constraints, the 6-fluoro-benzothiazole moiety of **1f** (cf. Fig. 1C) adopted a conformation similar to the 5-chlorobenzoxazole of suvorexant. However, the 6-fluoro-benzothiazole ring of **1f** might be turned by 180° so that the fluoro substituent fills the same pocket as the chloro substituent of suvorexant. Since there are no interactions that favor one distinct conformation, we conducted the FEP calculation with the highest-scored binding pose represented in Fig. 2C. FEP calculations suggested only minor changes in relative binding free energy (ΔΔG) of − 0.25 kcal/mol for **1e** and − 0.39 kcal/mol for **1f**, compared to **JH112**. These promising findings indicated that our structural modifications did not affect binding affinity and led us to pursue the synthesis of these compounds.

### Chemistry

The synthesis strategy for new heteroaryl derivatives of suvorexant and **JH112** was based on our previously published work [22]. Our envisaged series of fluorine-substituted heteroaryl derivatives **1a–f** contained derivatives of suvorexant with alkyl-unsubstituted diazepane moiety and various heteroarenes (Fig. 1C, 5-fluoro-benzoxazole derivative **1a**, 5- and 6-fluorobenzothiazole derivatives **1b** and **1c**, and 6-fluoroquinoxaline derivative **1d**) and derivatives of **JH112** with (*S,S*)-*sec*-butyl-substituted diazepane moiety bearing the heteroaryl scaffolds 5-fluorobenzoxazole and 6-fluorobenzothiazole (Fig. 1C, compounds **1e** and **1f**).

**Scheme 1:**
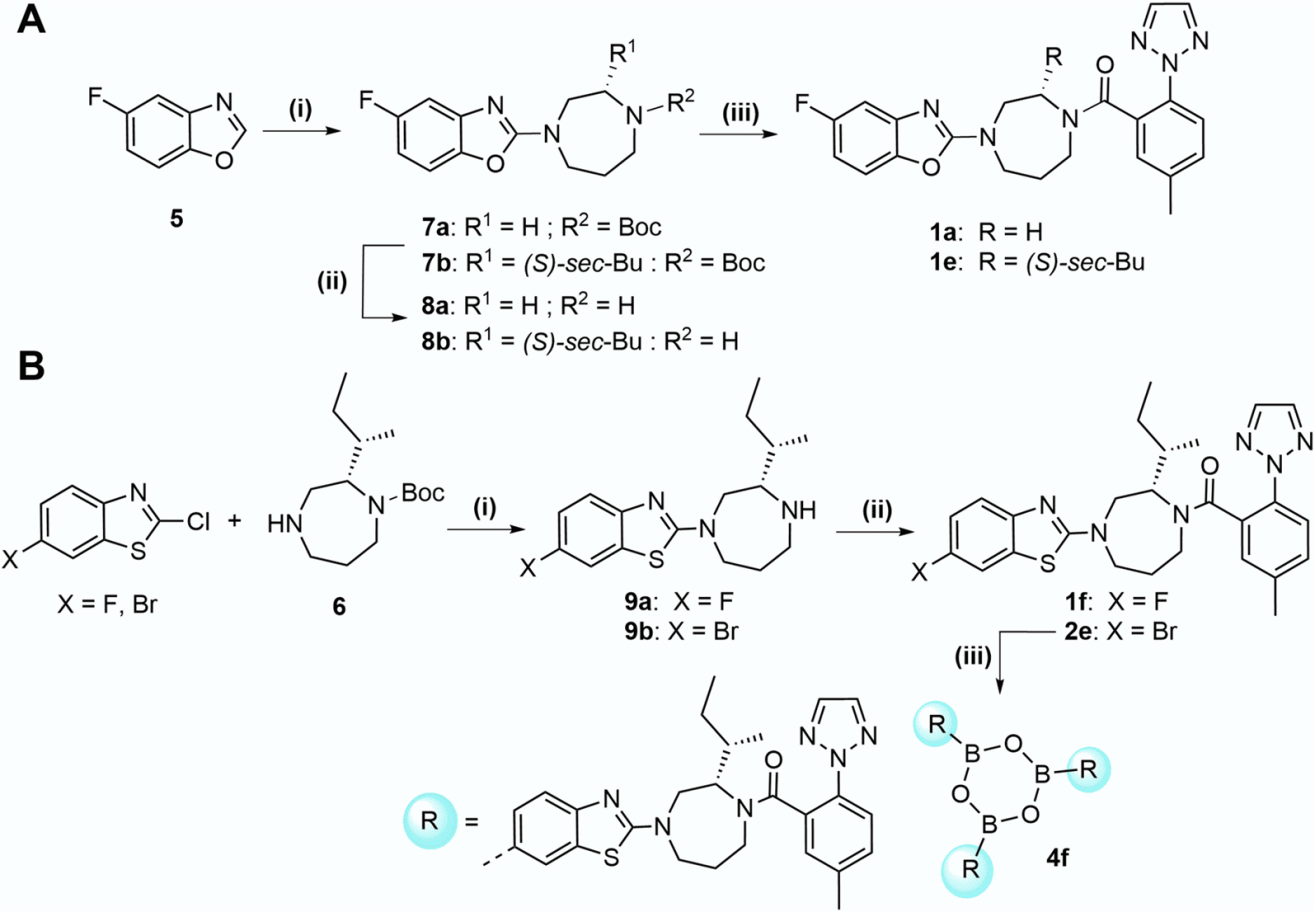
Synthesis of fluorine-substituted OXR ligands **1a**, **1e** and **1f**, and the corresponding boroxine **4f** for ^18^F-labeling. **A** Reagents and conditions: (i) *tert*-butyl 1,4-diazepane-1-carboxylate (R^1^ = H) or **6** (R^1^ = *(S)-sec*-Bu), Cu(OAc)_2_·H_2_O, CH_3_CO_2_H, CH_3_CN, air, 80 °C, 26–46 h, 32–36%; (ii) TFA, DCM, rt, 4–5 h, quant.; (iii) 5-methyl-2-(2*H*-1,2,3-triazol-2-yl)benzoic acid, HATU, DIPEA, DMF, rt, 3–24 h, 30–51%. **B** Reagents and conditions: (i) 1. DMF. K_2_CO_3_, 60 °C, 2–10 d, 2. TFA, DCM, rt, 3 h, **9a**: 49% and **9b**: 30%; (ii) 5-methyl-2-(2*H*-1,2,3-triazol-2-yl)benzoic acid, HATU, DIPEA, DMF, rt, 48–49%; (iii) 1. bis(pinacolato)diboron, KOAc, dioxane, Pd(dppf)Cl_2_, 85 °C, 24 h, 2. MeB(OH)_2_, NaOH (aq.), acetone, rt, o/n, 3. toluene, reflux, 100 °C, 300 mbar, 7 h, 13%

Starting from 5-fluorobenzoxazole [26], copper-catalyzed oxidative coupling with either commercially available *tert*-butyl 1,4-diazepane-1-carboxylate or the (*S,S*)-*sec*-butyl substituted homopiperazine building block **6**, synthesized as previously published [22], provided compounds **7a** and **7b** (Scheme 1A). It should be noted that the synthesis of the chiral homopiperazine **6** was a bottleneck, as a pronounced co-evaporation of **6** with solvents was observed. It is therefore recommended to use stock solutions of crude **6** in DMF for further reactions (see Supplementary Information). Cleavage of the Boc protecting group under acidic conditions gave the secondary amines **8a** and **8b**, which were coupled to commercially available 5-methyl-2-(2*H*-1,2,3-triazol-2-yl)benzoic acid under standard amide coupling conditions. The final compounds **1a** and **1e** were obtained after purification by preparative HPLC (Scheme 1A).

Building block **6** was also subjected to the coupling of commercially available 2-chloro-benzo[*d*]thiazole substituted in position 6 with either bromine or fluorine by nucleophilic aromatic substitution (Scheme 1B). After cleavage of the Boc protecting group, **9a** and **9b**, respectively, were subjected to amide bond formation with 5-methyl-2-(2*H*-1,2,3-triazol-2-yl)benzoic acid using HATU as coupling agent to afford the 6-fluorobenzothiazole **1f** and the 6-bromo derivative **2e**, respectively. The bromo compound **2e** was converted to the boronic acid pinacol (BPin) ester by Miyaura borylation reaction and further transesterification into the 6-boronic acid intermediate by addition of an excess of methylboronic acid [27]. Finally, the complete dehydration-induced condensation to provide boroxine **4f** was performed in toluene by azeotropic Dean-Stark extraction.

**Scheme 2:**
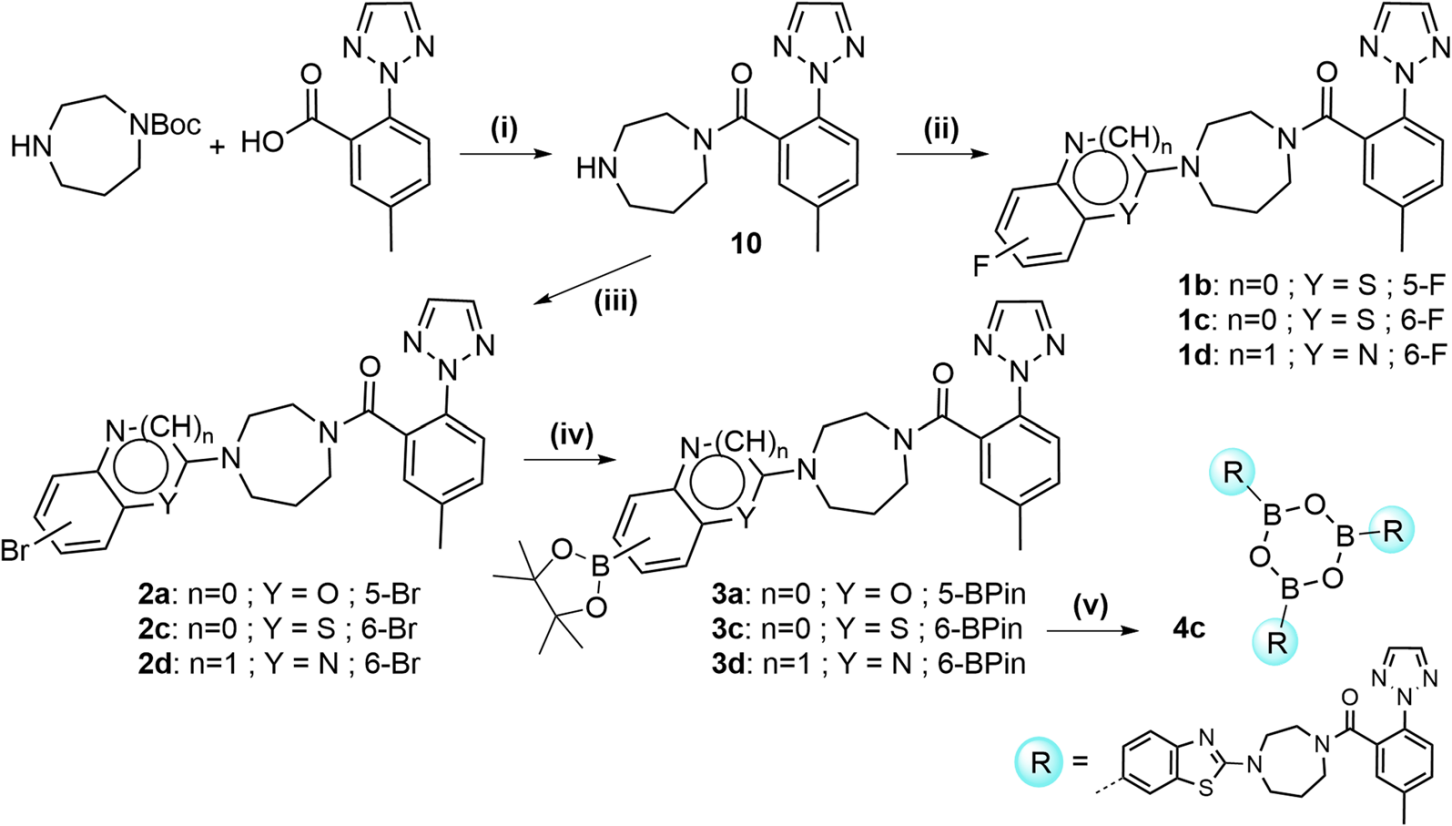
Synthesis of fluorine-substituted OXR ligands **1b**, **1c** and **1d**, and the corresponding BPin and boroxine precursors (**3a**, **3c**, **3d**, and **4f**) for ^18^F-labeling. Reagents and conditions: (i) 1. HATU, DIPEA, DMF, rt, o/n, 2. TFA, DCM, rt, 3 h, 81%; (ii) 2-chloro-5-fluoro-benzo[*d*]thiazole (for **1b**) or 2-chloro-6-fluoro-benzo[*d*]thiazole (for **1c**) or 2-chloro-6-fluoro-quinoxaline (for **1d**), DMF, K_2_CO_3_, 60 °C, 2–10 d, 7–55%; (iii) 5-bromo-benzo[*d*]oxazole, Cu(OAc)_2_·H_2_O, CH_3_CO_2_H, CH_3_CN, 75 °C, 3 d, 67% (for **2a**) or 6-bromo-2-chloro-benzo[*d*]thiazole (for **2c**) or 6-bromo-2-chloro-quinoxaline (for **2d**), DMF, K_2_CO_3_, 60 °C, 2–10 d, 7–70% (for (ii) and (iii)); (iv) bis(pinacolato)diboron, KOAc, dioxane, Pd(dppf)Cl_2_, 85 °C, 1–4 d, 55–99%; (v) 1. MeB(OH)_2_, NaOH (aq.), acetone, rt, o/n, 2. toluene, reflux, 100 °C, 300 mbar, 7 h, 99%

Scheme 2 shows the syntheses of suvorexant analogs **1b–d** bearing the alkyl-unsubstituted diazepane, to complete the series of target compounds **1a–f** (cf. Fig. 1C). Compared to Scheme 1, the order of reaction steps was changed allowing simplified protocols for the straightforward introduction of different heteroaryl scaffolds (Scheme 2). Amide coupling of commercially available *tert*-butyl 1,4-diazepane-1-carboxylate with 5-methyl-2-(2*H*-1,2,3-triazol-2-yl)benzoic acid in the first reaction step was followed by nucleophilic substitution on the respective heteroaryl compounds. The course of the syntheses shown in Scheme 2 used the same reaction types as documented in Scheme 1, providing compounds **1b–d** with the variation of the heteroarenes benzoxazole, benzothiazole and quinoxaline. Thus, the series of new OXR ligands was successfully synthesised in purities of > 98% (HPLC). In addition, the BPin esters **3a**, **3c** and **3d** were successfully obtained as well as the boroxines **4c** and **4f**, which were selected as labeling precursors for Cu-mediated aromatic ^18^F-fluorination after evaluation of OXR binding affinities of the non-radioactive compounds **1a–f**.

### Determination of OXR binding affinities of 1a–f

In comparison with the lead **JH112**, receptor binding affinities of the test compounds **1a–f** have been determined by radioligand displacement studies, as described previously [22], applying membranes of HEK293T cells transiently expressing the human OX1R or OX2R (Table 1).

**Table 1:** Receptor binding affinities of the OXR ligands **1a-f** in comparison with the lead compound **JH112** to the human receptor subtypes OX1R and OX2R


Compound	Y	R	Hal	K_i_ values (nM ± SEM)		OX_1_R selectivity^c^
				hOX1R^a^	hOX2R^b^	K_i_(hOX2R)/K_i_(hOX1R)
**JH112**^d^	O	(*S*)-*sec*-Bu	5-Cl	0.72 ± 0.08	54 ± 7.0	75
**1a**	O	H	5-F	2.5 ± 0.62	2.5 ± 0.36	1.0
**1b**	S	H	5-F	1.9 ± 0.42	2.4 ± 0.49	1.3
**1c**	S	H	6-F	0.99 ± 0.25	1.2 ± 0.20	1.2
**1d**	–	–	–	1.2 ± 0.40	0.83 ± 0.23	0.7
**1e**	O	(*S*)-*sec*-Bu	5-F	1.2 ± 0.28	92 ± 14	77
**1f**	S	(*S*)-*sec*-Bu	6-F	0.69 ± 0.26	53 ± 7.8	77

K_i_ values (nM) are given as mean ± SEM of at least three independent experiments, each done in triplicate

^a^Membranes from HEK293T cells transiently expressing the human OX1R were incubated with the radioligand [^3^H]SB974042. ^b^Homogenates from HEK293T cells which transiently express the human OX2R were incubated with the radioligand [^3^H]EMPA. ^c^Calculated by dividing K_i_ for OX2R by the K_i_ for OX1R. ^d^Data from previous work[22]

The results confirmed that replacing chlorine in **JH112** with fluorine (**1e**) only marginally decreased OX1R affinity by a factor of 1.6. All derivatives lacking the *(S,S)-sec*-butyl substituent at the diazepane ring (**1a–d**) showed one-digit nanomolar affinities for both OXR subtypes in the range from 0.8 nM (K_i_(OX2R), **1d**) to 2.5 nM (K_i_(OX1R), **1a**), confirming that changing the heteroarene scaffold from 5-chlorobenzoxazole in **JH112** to 5-fluorobenzothiazole (**1b**), 6-fluorobenzothiazole (**1c**) or 6-fluoroquinoxaline (**1d**) is well tolerated by both OXR subtypes. Interestingly, introduction of fluorine in 6-position of the benzothiazole (**1c**) demonstrated twofold increased OXR affinities compared to 5-fluorobenzothiazole **1b**. Consequently, the combination of the 6-fluorothiazole scaffold with the (*S,S*)-*sec*-butyl substituent at the diazepane ring led to **1f**, demonstrating excellent OX1R affinity with a K_i_ value of 0.69 nM and 77-fold selectivity for OX1R over OX2R. Compared to **JH112**, the benzothiazole **1f** perfectly mimicked the OXR binding affinities and OX1R selectivity, thus suggesting the ^18^F-labeled **1f** as a promising PET ligand candidate.

### Cu-mediated ^18^F-fluorination

**Scheme 3:**
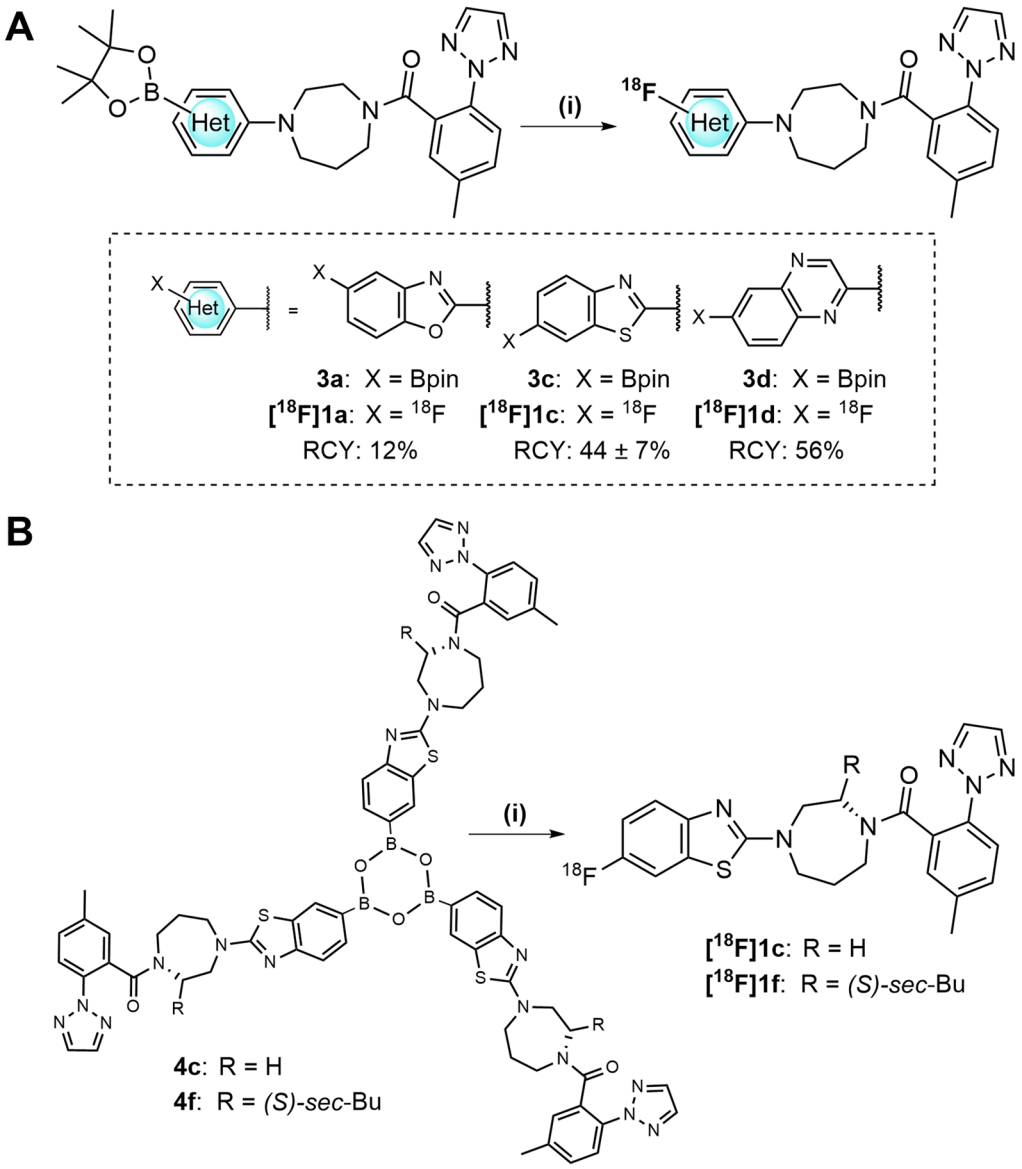
Cu-mediated ^18^F-fluorination of **3a**, **3c** and **3d** to achieve **[**^**18**^**F]1a**, **[**^**18**^**F]1c**, and **[**^**18**^**F]1d**. **A** Reagents and conditions: (i) 1. Elution of [^18^F]fluoride (6.2 mM TEAB in methanol); 2. [Cu(OTf)_2_py_4_] (6.42 µmol) and **3a**, **3c** or **3d** (7.08 µmol) in DMA:n-BuOH (2:1, 200 µL), 110 °C, air; RCY were determined after 10 min (for **[**^**18**^**F]1c** and **[**^**18**^**F]1d**) and after 20 min (for **[**^**18**^**F]1a**). **B** Reaction conditions: (i) see **A**, using **4c** or **4f** (2.36 µmol) and a reaction time of 5 min

Based on the established reaction conditions of Cu-mediated ^18^F-fluorination [28] and our own preliminary work on the heterocyclic aromatic model compounds benzoxazoles, benzothiazoles and quinoxalines [29], we performed comparative ^18^F-labeling experiments on the BPin esters **3a**, **3c** and **3d** to obtain the ^18^F-labeled ligands **[**^**18**^**F]1a**, **[**^**18**^**F]1c**, and **[**^**18**^**F]1d**. The results are shown in Scheme 3A.

As expected, the benzoxazole precursor **3a** yielded only a low RCY of 12%, confirming previously published results on model benzoxazoles [23, 29]. Fortunately, the 6-BPin esters of benzothiazole (**3c**) and quinoxaline (**3d**) were labeled under optimized reaction conditions with RCY of 44% (**[**^**18**^**F]1c**) and 56% (**[**^**18**^**F]1d**) after 10 min using the solvent system n-butanol/DMAA (2:1) and maintaining a precursor-to-Cu catalyst ratio of 1.1 (Scheme 3A).

In addition, the benzothiazole boroxines **4c** and **4f** were successfully used for Cu-mediated ^18^F-fluorination (Scheme 3B). Based on our preliminary studies on ^18^F-fluorination of boroxines [30], the ^18^F-labeling of **4c** and **4f** to yield **[**^**18**^**F]1c** and **[**^**18**^**F]1f**, respectively, was performed with a shortened reaction time of 5 min, since the analysis of samples taken from the reaction mixture in a time-dependent manner showed sufficient RCY of **[**^**18**^**F]1c** at this time point (2.5 min: 31%, 5 min: 45%, and 7 min: 52%). Compared to the BPin ester **3c** (RCY of 44% for **[**^**18**^**F]1c** at 10 min, Scheme 3A), the use of boroxine **4c** (RCY of 45% for **[**^**18**^**F]1c** at 5 min) suggests an accelerated ^18^F-fluorination reaction, however, further experiments with different heteroaromatic boroxines are needed to verify this result. The boroxines reacted as trimeric precursors, so that the precursor-to-Cu catalyst ratio of 0.37 was applied. Initial experiments suggested that the use of boroxine **4c** led to an increase in the apparent molar activity (AMA) obtained at EOS for **[**^**18**^**F]1c** by a factor of about 2, compared to the corresponding benzothiazole 6-BPin precursor **3c** (AMA (**[**^**18**^**F]1c** from **4c**): 6.0–6.7 GBq/µmol versus AMA (**[**^**18**^**F]1c** from **3c**): 3.6 GBq/µmol). **[**^**18**^**F]1c** was obtained with an overall activity yield (AY) of 3–14% (referred to [^18^F]fluoride) after final HPLC purification and formulation within a total radiosynthesis time of 50–60 min. Boroxine precursor **4f** was subjected to Cu-mediated radiofluorination, isolating **[**^**18**^**F]1f** with the AY of 22% (referred to [^18^F]fluoride) and an AMA of 2.2 GBq/µmol. The final HPLC purification of **[**^**18**^**F]1c** and **[**^**18**^**F]1f** turned out to be problematic, as overlapping co-elution of byproducts from protodeboronation and the broad peak in the case of **[**^**18**^**F]1f** due to rotamers made this step challenging, thereby limiting the apparent molar activity. Due to the excellent in vitro OX1R affinity of **1f** (K_i_ = 0.69 nM) and 77-fold selectivity over OX2R, **[**^**18**^**F]1f** together with its non-selective OXR analog **[**^**18**^**F]1c** were studied in further in vitro and initial in vivo studies.

### In vitro evaluation of [^18^F]1c and [^18^F]1f

After purification of **[**^**18**^**F]1c** and **[**^**18**^**F]1f** by semi-preparative radio-HPLC and formulation in 0.9% saline solution, logD_7.4_ values, plasma protein binding (PPB), and stability in plasma and serum were determined for **[**^**18**^**F]1c** and **[**^**18**^**F]1f**. **[**^**18**^**F]1c** showed a logD_7.4_ value of 2.28 ± 0.38 (n = 9), which was slightly higher for **[**^**18**^**F]1f** (2.37 ± 0.11, n = 6), reflecting the influence of the *(S,S)-sec-*butyl substituent. Determination of PPB gave 66% for **[**^**18**^**F]1c** and 77% for **[**^**18**^**F]1f**, indicating that a limited fraction of free **[**^**18**^**F]1c** and **[**^**18**^**F]1f** could be available for penetration of the blood–brain-barrier (BBB). Furthermore, both **[**^**18**^**F]1c** and **[**^**18**^**F]1f** remained stable over 120 min at 37 °C after incubation in rat and human plasma and human serum.

### Initial small animal PET imaging of [^18^F]1c and [^18^F]1f

**Fig. 3:**
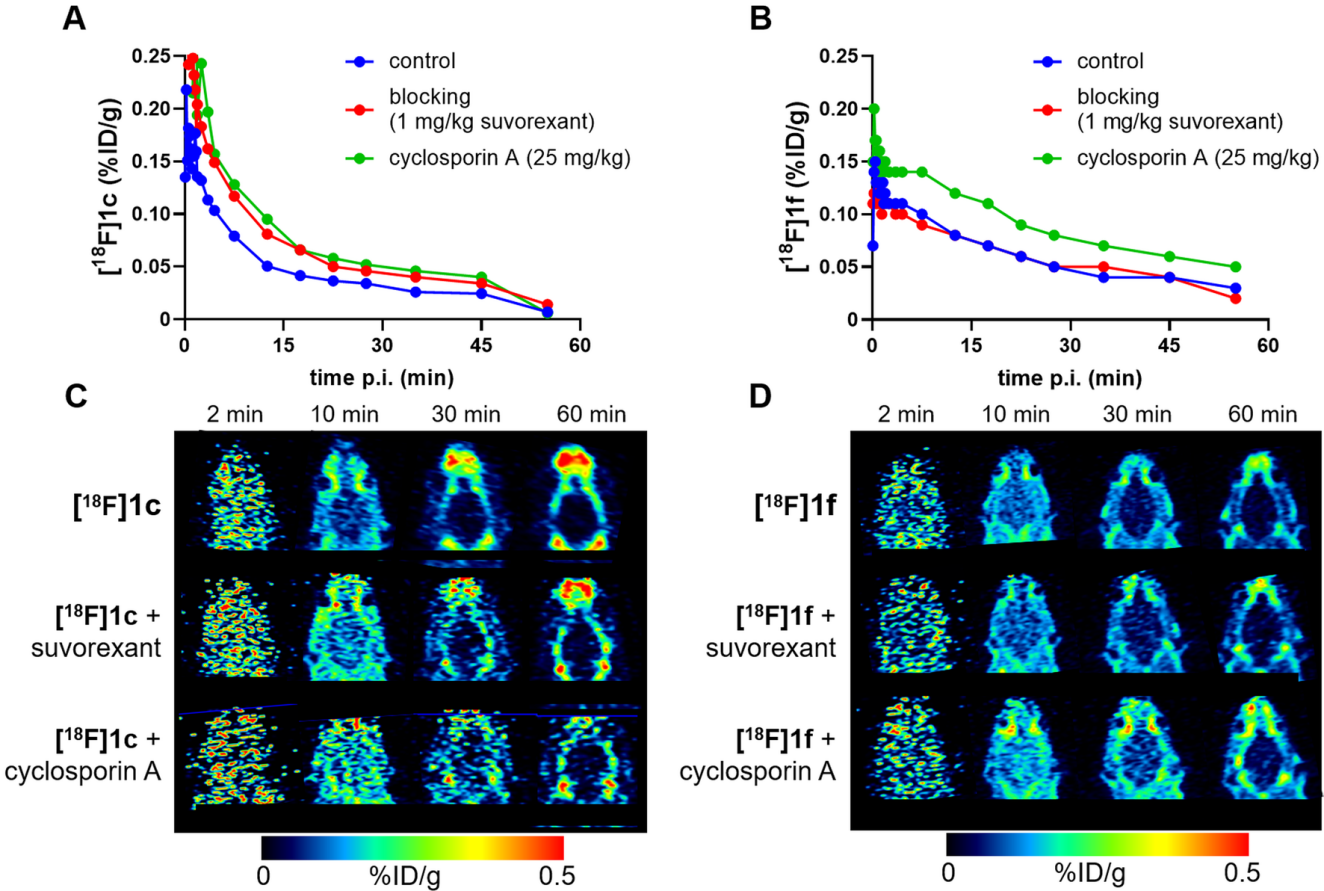
Time-activity curves and horizontal PET slices of the rat brain after injection of **[**^**18**^**F]1c** (**A**, **C**) and **[**^**18**^**F]1f** (**B**, **D**). Single animals were injected with the tracer alone (“control”, blue) compared to animals preinjected with suvorexant (1 mg/kg, 15 min prior to tracer injection, each n = 1; “blocking”, red) or cyclosporine A (25 mg/kg, 30 min prior to tracer injection, each n = 1; green)

Small animal PET experiments in rats were performed to allow for an initial comparison in single animals that were injected with **[**^**18**^**F]1c** or **[**^**18**^**F[1f** alone, after preinjection of suvorexant (1 mg/kg), and after pretreatment with cyclosporine A (25 mg/kg) (Fig. 3). After injection of **[**^**18**^**F]1c** in rats, a maximum brain uptake of 0.17%ID/g was observed at about 40 s p.i., whereas preinjection of suvorexant or cyclosporine A both induced increased brain uptake to 0.25%ID/g. **[**^**18**^**F]1c** showed homogenous distribution in the brain with fast clearance from 2 to 15 min p.i. without indication of retention in any brain region. In comparison, **[**^**18**^**F]1f** demonstrated brain uptake with a maximum of 0.15%ID/g at 24 s p.i., that was not affected by preinjection of suvorexant, but was marginally increased to 0.18%ID/g by pretreatment with cyclosporine A. Preinjection of suvorexant, however, did not significantly block brain uptake of either **[**^**18**^**F]1c** nor **[**^**18**^**F[1f** at any time point after tracer injection. Instead, both radioligands demonstrated clearance from the brain without specific binding to any brain region. The increased brain uptake of **[**^**18**^**F]1c** and **[**^**18**^**F]1f** by pretreatment with cyclosporine A could be attributed to several reasons, such as: a) **[**^**18**^**F]1c** and **[**^**18**^**F]1f** could be potential substrates of the efflux transporter P-gp in the BBB, and inhibition of P-gp by cyclosporine A would therefore increase the brain uptake of **[**^**18**^**F]1c** and **[**^**18**^**F]1f**, b) cyclosporine A binds to plasma lipoproteins [31] and its metabolism may interfere with that of suvorexant derivatives, both of which could result in an increased free fraction of **[**^**18**^**F]1c** and **[**^**18**^**F]1f** in the blood, thereby increasing brain uptake, and c) the known ability of cyclosporine A to increase the permeability of brain endothelial cells in vitro [32]. Therefore, to elucidate the P-gp-mediated transport of the compounds, specific experiments were performed.

### Determination of P-gp-mediated transport

**Fig. 4:**
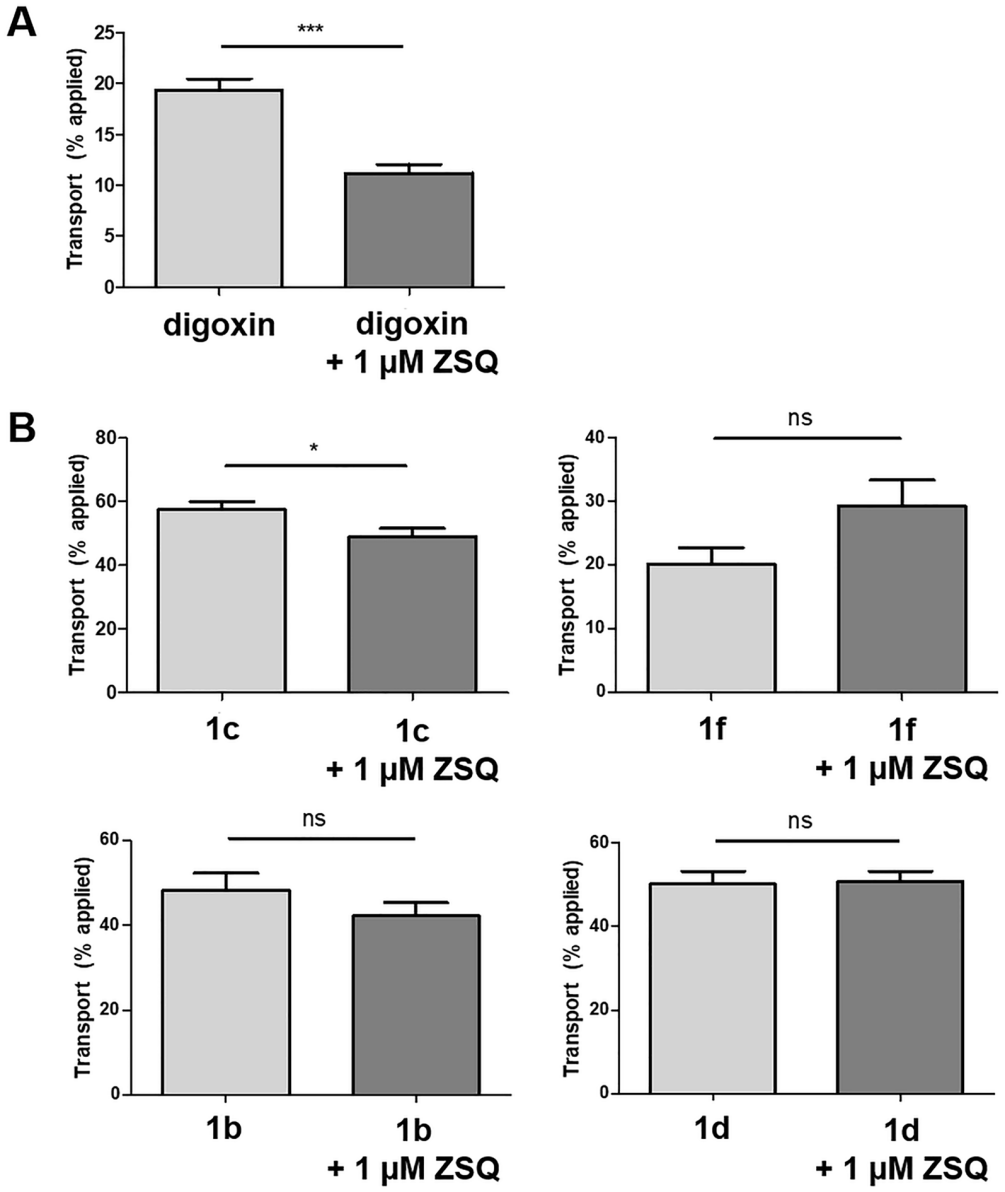
Basolateral-to-apical transport of the P-gp substrate digoxin in P-gp expressing Caco-2 monolayers in the absence and presence of the P-gp inhibitor zosuquidar (ZSQ) (**A**) compared to basolateral-to-apical transport of **1c**, **1f**, **1b** and **1d** in the presence and absence of ZSQ (**B**). Amounts being present after 4 h in the apical compartment are shown in % of the amount administered to the basal side. Digoxin and all test compounds were each used in a concentration of 5 µM (ns: p ≥ 0.05, *p < 0.05, ***p < 0.001, n = 8)

Compounds **1c** and **1f**, together with **1b** and **1d**, were studied in basolateral-to-apical transport experiments applying the Caco-2 cell monolayer model, to determine potential P-gp-mediated cellular efflux by semi-quantitative LC–MS analysis. As expected, basolateral-to-apical transport of the P-gp substrate digoxin was significantly inhibited in the presence of the P-gp inhibitor zosuquidar (ZSQ, LY335979 [33]; Fig. 4A). Interestingly, compounds **1b**, **1c** and **1d** showed relatively high passive permeability that was even greater than that of digoxin, whereas the transcellular passive diffusion of **1f** was similarly high as with digoxin (Fig. 4B). Inhibition of P-gp by ZSQ did not significantly reduce the apical concentration of **1b**, **1d** and **1f** (Fig. 4B) and had only a very modest, yet significant, effect on **1c** compared to the highly significant effect of ZSQ on P-gp-mediated basolateral-to-apical efflux of digoxin (Fig. 4A), suggesting that P-gp does not play a relevant role in limiting brain accumulation of all four molecules in vivo, including **[**^**18**^**F]1c** and **[**^**18**^**F]1f**.

## Discussion

Docking and FEP calculations indicated that the fluoro-substituted compounds **1e** and **1f**, could be accommodated in the orthosteric binding pocket of the OX1R and displayed a binding free energy comparable to **JH112**. Radioligand displacement studies confirmed that the predicted binding affinities were in good agreement with the experimentally determined data.

Supported by computational analysis, we successfully applied medicinal chemisty methods with integrated receptor binding experiments to synthesize and characterize the new series of OXR ligands **1a–f**, identifying the 6-fluoro-benzothiazole **1f** as an excellent analog of **JH112** with retained OX1R affinity (K_i_ = 0.69 nM) and 77-fold selectivity over the OX2R subtype. The multiple-step synthesis of **1f** followed the strategy of the synthesis of **JH112** [22].

Based on the in vitro OXR affinities, the target compounds **[**^**18**^**F]1a**, **[**^**18**^**F]1c** and **[**^**18**^**F]1d** were first investigated in comparative ^18^F-labeling experiments, applying the corresponding BPin esters **3a**, **3c** and **3d**. Cu-mediated aromatic ^18^F-fluorination of BPin esters using n-butanol/DMA as solvent system is well established [28]. In preliminary Cu-mediated ^18^F-fluorination experiments with BPin esters of simple aromatic model compounds benzoxazoles, benzothiazole and quinoxaline [29], we and others have demonstrated that 5-benzoxazole BPin esters provide only a limited RCY of 20–30% after 20 min for Cu-mediated ^18^F-fluorination [23, 29]. Confirmed by our results on **[**^**18**^**F]1a** (RCY of 12%), the benzoxazole scaffold was therefore considered not very suitable for application in Cu-mediated aromatic ^18^F-fluorination. By isosteric exchange of the benzoxazole with benzothiazole this radiochemical problem could be circumvented and by shifting the 5-position of the fluorine substitution to the 6-position a potential PET ligand was found with **[**^**18**^**F]1f**, which was successfully obtained by preparative ^18^F-synthesis in an adequate overall activity yield of up to 22%.

For Cu-mediated aromatic ^18^F-fluorination of BPin esters, it is known that pronounced losses of radioactivity occur during the work-up of the reaction. In addition, non-radioactive by-products, due to protodeboronation and oxidation (formation of the phenol) [34], increase during the ^18^F-labeling reaction. According to our own experience and reports by others [29, 30, 35], the Cu-mediated ^18^F-fluorination of N- or O- and N-containing heteroaromatic compounds takes up to 20 min. In order to avoid the increased formation of by-products, the reaction time of Cu-mediated ^18^F-fluorination should always be kept as short as possible. More stable precursor molecules, such as stannanes [36], could also be beneficial to avoid interfering by-products. In the present work, we proposed boronic acid anhydrides, i.e. boroxines, as alternative labeling precursors instead of BPin esters, and showed that boroxine **4c** in Cu-mediated ^18^F-fluorination gave an increased apparent molar activity of **[**^**18**^**F]1c** by a factor of 2, compared to the BPin ester **3c** after 5 min. To the best of our knowledge, there is only one previous report on the use of a boroxine-pyridine adduct for ^18^F-labeling [37]. Therefore, more detailed comparative studies of boroxines and BPin precursors for Cu-mediated ^18^F-fluorination would be interesting and are currently underway in our laboratory to elucidate potential advantages of boroxines in terms of achievable molar activity.

In initial PET experiments of **[**^**18**^**F]1c** and **[**^**18**^**F]1f** in rats, adequate brain permeability was observed. The brain uptake was consistent with the relatively high passive permeability of **[**^**18**^**F]1c**, which was confirmed in vitro applying the Caco-2 monolayer model. Brain uptake was limited by the high protein-bound fraction of **[**^**18**^**F]1c** and **[**^**18**^**F]1f** in the blood, which was slightly higher for **[**^**18**^**F]1f** (77%) than for **[**^**18**^**F]1c** (66%) in vitro, reflecting the difference in brain uptake. The high proportion of the protein-bound fraction in the blood is also known for suvorexant (> 99% [38]), as is the very rapid clearance and high uptake in the intestine, which correlates with the passive permeability of suvorexant [39, 40].

No specific binding of **[**^**18**^**F]1c** and **[**^**18**^**F]1f** could be detected by PET imaging at any time after tracer injection in the rat brain. On the one hand, this may be due to the relatively low AMA of the radioligands, as the tracers were produced in manual syntheses starting from 1 GBq of [^18^F]fluoride; on the other hand, a higher free fraction of radioligands in the blood is desireable, to increase the initial brain uptake of PET ligand candidates.

In the case of **[**^**18**^**F]1c**, the pre-injection of highly plasma protein-bound suvorexant may have led to an increase in the free fraction of **[**^**18**^**F]1c** in the blood, so that the observed increased initial brain uptake of **[**^**18**^**F]1c** could be explained. This was not the case for **[**^**18**^**F]1f**. **[**^**18**^**F]1f** showed a lower passive permeability in vitro than **[**^**18**^**F]1c**, suggesting that an increased free fraction in blood of **[**^**18**^**F]1f** caused by suvorexant pre-injection could presumably have a lower effect on brain uptake compared to **[**^**18**^**F]1c**.

Pre-injection of cyclosporine A increased the brain uptake of **[**^**18**^**F]1c** and **[**^**18**^**F]1f**. However, cyclosporine A is metabolized by CYP3A4 [41], which is also the most important enzyme for the metabolism of suvorexant [42], thereby most likely influencing the plasma levels of **[**^**18**^**F]1c** and **[**^**18**^**F]1f** to increase apparent brain uptake. In addition, cyclosporine A is known to increase the permeability of brain endothelial cells in vitro and inhibits adrenomedullin-mediated regulation of endothelial barrier function [32, 43]. Such effects are not related to P-gp inhibition and could be the reason for the observed increased brain uptake of **[**^**18**^**F]1c** and **[**^**18**^**F]1f** after cyclosporine A pretreatment with the relatively high dose of 25 mg/kg. Our in vitro experiments in the Caco-2 monolayer model, using the P-gp inhibitor zosuquidar, demonstrated that **1c** and **1f** were not efficient P-gp substrates, thereby suggesting that P-gp-does not limit brain accumulation of **[**^**18**^**F]1c** and **[**^**18**^**F]1f** in vivo.

## Conclusion

The results on **[**^**18**^**F]1f** as an OX1R-selective radioligand and its non-selective analog **[**^**18**^**F]1c** provide valuable information for the development of suitable OXR PET ligands for brain imaging. In particular, future work should consider structural modifications that lead to lower plasma protein binding and the development of radiolabeling protocols that enable preparation of the tracers with improved apparent molar activities.

## Materials and methods

General information on materials, compounds **5** and **6**, the synthesis and analytical data of compounds **1a–f**, **2a**, **2c**, **2d**, **2e**, **3a**, **3c**, **3d**, **4c**, **4f**, **7a**, **7b**, **8a**, **8b**, **9a**, **9b**, and **10**, NMR spectra and chromatograms obtained by HPLC analysis of ^18^F-labeled and respective reference compounds are provided in the Supplementary Information file.

### Computational analysis

#### System setup

The high-resolution crystal structure of the OX1R in complex with suvorexant (PDB: 6TO7 [24]) was employed for docking and FEP calculations. This choice was based on its detailed representation of water molecules within the orthosteric binding pocket and the structural similarity of the ligand to compounds **JH112**, **1e**, and **1f**. Prior research has demonstrated the critical role of these water molecules in achieving accurate FEP results at the OX2R [44]. The receptor preparation involved extracting coordinates for the receptor, water molecules, a sodium ion, and the ligand from chain A. Any introduced mutations were reverted, and the missing amino acid residue at position Q246^5.69^ was modeled using PyMOL [45]. Subsequent preparation steps were carried out using the Protein Preparation Wizard [46] module of the Schrödinger Suite 2021.3. This process included modeling missing side chains, removing water molecules located further than 5 Å from the ligand, optimizing hydrogen bonds, and setting protein protonation states at pH 7.4. Notably, the protonation states of histidines H216^5.39^ and H344^7.39^ were adjusted due to their proximity to aspartate D107^2.65^ and glutamate E204^45.52^, respectively. Previous FEP studies highlighted the influence of histidine protonation on calculation accuracy [44]. The System Builder module was utilized to integrate the receptor into a 1-palmitoyl-2-oleoyl-*sn-*glycero-3-phosphocholine (POPC) lipid membrane surrounded by SPC water molecules in an orthorhombic box, using the OPLS4 force field [47]. The electronic charge of the system was neutralized, and a 0.15 M NaCl concentration was established. Finally, the system underwent equilibration using the default relaxation protocol with Desmond.

#### Docking

Molecular docking was performed using the Glide module [48]. Low-energy conformations of the ligands **JH112**, **1e**, and **1f** were prepared with the LigPrep tool, ensuring the retention of chiral centers. Coordinates for the suvorexant-bound OX1R were extracted from the previously described equilibrated system. A docking grid centered on the centroid of suvorexant was created, measuring 25 × 25 × 25 Å^3^. Docking was executed following a protocol established in earlier studies [25], which included applying core constraints to the maximum common substructure (MCS) of suvorexant with a tolerance of 2 Å. The docking was carried out at standard precision settings.

#### FEP calculations

FEP calculations were conducted using the FEP + module [49], to determine the relative free energy changes (ΔΔG) of ligands **1e** and **1f** compared to **JH112**. These calculations were performed under default settings, which included: (a) 12 *λ*-windows; (b) 5 ns of simulation per *λ*-window, utilizing replica exchange; and (c) the *μ*VT ensemble. Any missing parameters for the ligands were generated using the Force Field Builder module.

Figures illustrating the computational results were generated using PyMOL [45] and ChimeraX [50] software.

### Preparation of [^18^F]fluoride

No-carrier-added [^18^F]fluoride was produced by irradiation of H_2_[^18^O]O on a PETtrace 800 cyclotron (General Electric, Uppsala, Sweden) and purchased from Universitätsklinikum Würzburg, Germany. The aqueous [^18^F]fluoride solution was loaded onto a Sep-Pak® Light (46 mg) Accel™ Plus QMA carbonate cartridge from the male side. The cartridge was washed with dry acetone (2 mL) from the male side and air (10 mL) was passed over the cartridge from the female side. Subsequently, [^18^F]fluoride was eluted directly into a preheated (85 °C) reaction vial applying a methanolic solution of tetraethylammonium bicarbonate (TEAB, 6.2 mM, 500 µL). The solvent was removed in a stream of helium.

### Radiosynthesis of [4-(5-[^18^F]fluorobenzo[*d*]oxazol-2-yl)-1,4-diazepan-1-yl][5-methyl-2-(2*H*-1,2,3-triazol-2-yl)phenyl]methanone ([^18^F]1a)

The reaction vial containing dry [^18^F]fluoride/TEAB (36 MBq) was heated to 110 °C. Afterwards, a solution of the corresponding BPin precursor **3a** (7.1 µmol) and [Cu(OTf)_2_py_4_] (6.4 µmol) in DMA and *n*-BuOH (2:1, 200 µL) was added to the reaction vial and the reaction was allowed to stir under air. Radioligand **[**^**18**^**F]1a** was obtained after 20 min in a RCY of 12% (n = 1). R_f_ (**[**^**18**^**F]1a**) = 0.75 (silica TLC, ethyl acetate); HPLC analysis (Chromolith Performance RP-18e, 100 × 4.6 mm, 4 mL/min, 10–100% CH_3_CN in H_2_O (0.1% TFA) in 5 min): co-injection of **[**^**18**^**F]1a** (t_R_ = 2.93 min) and **1a** (t_R_ = 2.88 min).

### Radiosynthesis of [4-(6-[^18^F]fluorobenzo[*d*]thiazol-2-yl)-1,4-diazepan-1-yl][5-methyl-2-(2*H*-1,2,3-triazol-2-yl)phenyl]methanone ([^18^F]1c)

a) Starting from boroxine precursor **4c**: The reaction vial containing dry [^18^F]fluoride/TEAB (1235–1245 MBq) was heated to 110 °C. Afterwards, a solution of the corresponding boroxine precursor **4c** (2.4 µmol) and [Cu(OTf)_2_py_4_] (6.4 µmol) in DMA and *n*-BuOH (2:1, 200 µL) was added into the reaction vial and the reaction was stirred under air. After 5 min the reaction mixture was quenched in a solution of CH_3_CN in H_2_O (20%, 7 mL) and passed over a preconditioned HLB prime cartridge (100 mg). The cartridge was washed with H_2_O (10 mL) and the trapped radioligand was eluted with methanol (2 mL) into a v-vial. The solvent was removed under a stream of helium at 85 °C. CH_3_CN in H_2_O (80% + 0.1% TFA, 400 µL) was added and the solution was injected into the semi-preparative HPLC system (Luna PFP(2), 250 × 10 mm, 5 µm, 4 mL/min, CH_3_CN in H_2_O + 0.1% TFA: 42% for 5 min, 42–58% in 5–17 min). The fraction containing the ^18^F-labeled product was collected, diluted to a total volume of 23 mL with Milli-Q water and passed over a HLB prime cartridge (35 mg). The product **[**^**18**^**F]1c** was eluted into a pointed flask using methanol (2 mL). After evaporation of the solvent **[**^**18**^**F]1c** was formulated for further experiments in 0.9% saline solution. Radiochemical purity = 99% (n = 1); R_f_ (**[**^**18**^**F]1c**) = 0.5 (silica TLC, ethyl acetate); HPLC analysis (Chromolith Performance RP-18e, 100 × 4.6 mm, 4 mL/min, 10–100% CH_3_CN in H_2_O (0.1% TFA) in 5 min): co-injection of **[**^**18**^**F]1c** (t_R_ = 2.66 min) and **1c** (t_R_ = 2.59 min); A_m_ = 6–7 MBq/nmol, AY = 3–7% (referred to [^18^F]fluoride, total synthesis time: 52–57 min, n = 2).

b) Starting from BPin precursor **3c**: The reaction vial containing dry [^18^F]fluoride/TEAB (940 MBq) was heated to 110 °C. Subsequently, BPin precursor **3c** (7.1 µmol) and [Cu(OTf)_2_py_4_] (6.4 µmol) in DMA and *n*-BuOH (2:1, 200 µL) was added to the reaction vial and the reaction was stirred under air for 5 min. Product **[**^**18**^**F]1c** was purified as described above and was obtained in a radiochemical purity of 99%; A_m_ = 4 MBq/nmol, AY = 14% (referred to [^18^F]fluoride, total synthesis time: 60 min, n = 1).

### Radiosynthesis of [4-(6-[^18^F]fluoroquinoxalin-2-yl)-1,4-diazepan-1-yl][5-methyl-2-(2*H*-1,2,3-triazol-2-yl)phenyl]methanone ([^18^F]1d)

The reaction vial containing dry [^18^F]fluoride/TEAB (940 MBq) was heated to 110 °C. Subsequently, BPin precursor **3d** (7.1 µmol) and [Cu(OTf)_2_py_4_] (6.4 µmol) in DMA and *n*-BuOH (2:1, 200 µL) was added to the reaction vial and the reaction was stirred under air. Radioligand **[**^**18**^**F]1d** was obtained after 10 min with a RCY of 56% (n = 1). R_f_ (**[**^**18**^**F]1d**) = 0.4 (silica TLC, ethyl acetate); HPLC analysis (Chromolith Performance RP-18e, 100 × 4.6 mm, 4 mL/min, 10–100% CH_3_CN in H_2_O (0.1% TFA) in 5 min): Co-injection of **[**^**18**^**F]1d** (t_R_ = 2.99 min) and **1d** (t_R_ = 2.91 min).

### Radiosynthesis of {(*S*)-2-[(*S*)-*sec*-butyl]-4-(6-[^18^F]fluorobenzo[*d*]thiazol-2-yl)-1,4-diazepan-1-yl}[5-methyl-2-(2*H*-1,2,3-triazol-2-yl)phenyl]methanone ([^18^F]1f)

The reaction vial containing dry [^18^F]fluoride/TEAB (1133 MBq) was heated to 110 °C and a solution of **4f** (2.4 µmol) and [Cu(OTf)_2_py_4_] (6.4 µmol) in DMA and *n*-BuOH (2:1, 200 µL) was added and the reaction was stirred under air. After 5 min the reaction mixture was quenched in a solution of CH_3_CN in H_2_O (20%, 7 mL) and passed over a preconditioned HLB prime cartridge (100 mg). The cartridge was washed with H_2_O (10 mL) and the trapped radioligand was eluted with methanol (2 mL) into a v-vial. The solvent was removed under a stream of helium at 85 °C. CH_3_CN in H_2_O (80% + 0.1% TFA, 400 µL) was added and the solution was injected into the semi-preparative HPLC (Kromasil C8, 5 µm, 125 × 10 mm, 4 mL/min, CH_3_CN in H_2_O + 0.1% TFA: 55% for 3 min, 55–65% in 3–11.5 min, 65–85% in 11.5–17 min). The fraction containing the ^18^F-labeled product was collected, diluted to a total volume of 23 mL with Milli-Q water and passed over a HLB prime cartridge (35 mg). Product **[**^**18**^**F]1f** was eluted using methanol (2 mL). After evaporation of the solvent, a second HPLC purification (Luna PFP(2), 5 µm, 250 × 10 mm, 4 mL/min, 65% CH_3_CN in H_2_O + 0.1% TFA for 15 min) with subsequent solid phase extraction of **[**^**18**^**F]1f** as described above was applied yielding **[**^**18**^**F]1f** in a radiochemical purity of 99% (n = 1). R_f_ (**[**^**18**^**F]1f**) = 0.9 (silica TLC, ethyl acetate); HPLC analysis (Chromolith Performance RP-18e, 100 × 4.6 mm, 4 mL/min, 10–100% CH_3_CN in H_2_O (0.1% TFA) in 5 min): Co-injection of **[**^**18**^**F]1f** (t_R_ = 3.30 and 3.39 min) and **1f** (t_R_ = 3.16 and 3.27 min); A_m_ = 2.11 MBq/nmol, AY = 22% (referred to [^18^F]fluoride, total synthesis time: 76 min).

### Radioligand binding experiments

Binding affinities to the human orexin receptor subtypes OX1R and OX2R were determined as reported [22]. Membranes were prepared from HEK293T cells transiently transfected with the cDNA for the receptor (OX1R: human HCRTR1 from cDNA Resource Center, Bloomsburg University, Bloomsberg, PA; OX2R: human HCRTR2 from Genscript, Piscataway, NJ). For OX1R binding homogenates with receptor densities of B_max_ = 4200 ± 810 fmol/mg protein and binding affinities of *K*_*D*_ = 0.84 ± 0.09 nM were used. Determination of OX2R affinity was measured with membranes showing a B_max_ = 4900 ± 1100 fmol/mg protein and a *K*_*D*_ of 1.2 ± 0.12 nM. Competition binding experiments were conducted in binding buffer (50 mM Tris, 5 mM MgCl_2_, 0.1 mM EDTA, 5 µg/mL bacitracin and 5 µg/mL soybean trypsin inhibitor at pH 7.4) at final protein concentrations of 4 µg/well (OX1R) and 3 µg/well (OX2R) when incubating the OX1R specific radioligand [^3^H]SB674042 (specific activity: 43 Ci/mmol) or the OX2R radioligand [^3^H]EMPA (specific activity: 84 Ci/mmol) both at a final concentration of 0.7 nM (both purchased from Novandi, Södertälje, Sweden) and varying concentrations of the competing test compounds. Radioactivity was separated by filtration on GF/B glass fiber mats and counted with a scintillation counter (Microbeta from PerkinElmer, Rodgau, Germany). Non-specific binding was determined with 10 µM of SB674042 or EMPA. The protein concentration was determined applying the method of Lowry [51]. Competition curves were analyzed by nonlinear regression using the algorithms implemented in PRISM 10.2 (GraphPad Software, San Diego, CA) to get IC_50_ values, which were subsequently transformed into the K_i_ values employing the equation of Cheng and Prusoff [52].

### Determination of the partition coefficient (logD_7.4_)

Phosphate-buffered saline (PBS, 480 µL, pH 7.4) and *n*-octanol (500 µL) were placed in an Eppendorf tube. The radioligand (30–50 kBq, **[**^**18**^**F]1c** or **[**^**18**^**F]1f**) in PBS (20 µL) was added to the tube, vortexed for 1 min and centrifuged (20,000*g*, 1 min). Three samples (100 µL) from each layer were collected and measured by a γ-counter (Wallac Wizard 1470, PerkinElmer, USA). After the average cpm values from the triplicate measurements of each layer were calculated, the logD_7.4_ was determined as the logarithm of the ratio of *n*-octanol (mean cpm) to PBS (mean cpm). The mean logD_7.4_ value ± standard deviation was determined by at least three independent experiments each performed in triplicates.

### Determination of radioligand stability in human plasma, human serum or rat plasma

The radioligand (1–2 MBq, **[**^**18**^**F]1c** or **[**^**18**^**F]1f**) in PBS (20 µL) was incubated with human serum, human plasma or rat plasma (400 µL) at 37 °C. Samples (50 µL) were taken at 5, 10, 15, 30, 60 and 120 min, mixed with aqueous TFA (10%, 50 µL) and centrifuged (20,000*g*, 2 min). The supernatant (20 µL) was analyzed by radio-HPLC.

### Determination of plasma protein binding (PPB)

The radiotracer (100–200 kBq, **[**^**18**^**F]1c** or **[**^**18**^**F]1f**) in PBS (2 µL) was incubated with human plasma (100 µL) at 37 °C for 10 min. After preconditioning of the microcolumns according to the instructions of the manufacturer (illustra MicroSpin G-50 columns from GE Healthcare), a sample (40 µL) of was applied to the resin. The microspin tubes were centrifuged (2000*g*, 2 min) and both, eluate and resin were measured by a γ-counter (Wallac Wizard 1470, PerkinElmer, USA). The PPB was calculated as percentage of bound ligand (cpm of resin) from the total amount of radioactivity (sum of cpm of resin and eluate). Samples from incubation of the radiotracer in saline instead of plasma served as negative control. The mean PPB value was determined from at least two independent experiments each performed in triplicate.

### In vivo small animal PET imaging

Ten- to 13-week-old female CD Sprague–Dawley rats (250–300 g, Charles River) were housed with free access to standard rat chow (Purina) and water at all times. Dynamic small animal PET scans were performed on an Inveon™ microPET scanner (Siemens Healthineers AG, Erlangen, Germany). All rats were continuously anesthetized using isoflurane (2–3% in oxygen, 0.8 L/min) and placed on a heating pad (37 °C) during the PET scan. Rats were injected intravenously in the tail vein with the radioligand in saline (5–15 MBq in 200–300 µL, **[**^**18**^**F]1c** or **[**^**18**^**F]1f**). Pretreatment with suvorexant (1 mg/kg, 15 min prior to tracer injection) or cyclosporine A (25 mg/kg, 20 min prior to tracer injection) was done intravenously. Dynamic PET scans were acquired from 0 to 60 min p.i. and consisted of 23 frames with increasing duration (12 × 10 s, 3 × 1 min, 5 × 5 min and 3 × 10 min). Subsequently, an attenuation scan with a rotating Co-57 point source was performed. The obtained dynamic emission images were subjected to an attenuation and decay correction. After the iterative maximum a posteriori (MAP) estimation for the image reconstruction, regions of interest (ROIs) were drawn for the whole brain. Computational processing of the PET scans was done with the software PMOD (PMOD Technologies LLC). The data obtained were finally converted into values expressed as percent of injected dose per gram (%ID/g).

### Transport studies

In order to characterize compounds as substrates of P-gp, monolayers of Caco-2 cells were used in line with previous studies [53]. In brief, transport from the basolateral to apical compartment was determined after 4 h in the absence or presence of the P-gp inhibitor zosuquidar (Biomol GmbH, Hamburg, Germany). All putative substrates and digoxin were administered in concentrations of 5 µM to the basolateral compartment. Zosuquidar was administered in a concentration of 1 µM to the basal and apical compartments. Results are expressed in % of the amount administered to the basolateral compartment at the beginning of the experiment. The P-gp substrate digoxin (obtained in tritium-labeled form from ARC, St. Louis, USA and in unlabeled form from Merck KGaA, Darmstadt, Germany) was used as positive control. Monolayer integrity was checked routinely using tritium-labeled inulin (obtained from ARC, St. Louis, USA). The analyte-to-internal-standard-ratios of **1c**, **1f**, **1b** and **1d** were determined by LC–MS. Digoxin and inulin concentrations were determined by liquid scintillation counting (Tricarb 2800, Perkin Elmer Life Science GmbH, Germany). LC–MS analyses were performed using an ultra-high-performance liquid chromatography (UPLC) (Ultimate 3000, Thermo Fisher Scientific) coupled to a high-resolution mass spectrometer (QExactive Orbitrap Focus, Thermo Fisher Scientific). The analytical column was an Acquity UPLC BEH C18, 1.7 µm, 2.1 × 100 mm column (Waters GmbH) and 80% methanol with 0.1% formic acid was used as mobile phase during an isocratic elution for 5 min at a flow rate of 0.35 mL/min. For sample preparation, 200 µL cell culture supernatant were mixed with 600 µL methanol including 50 ng/mL clopidogrel-d3 as an internal standard. Samples were centrifuged, the solvent of 600 µL supernatant was evaporated to dryness and 100 µL mobile phase was used for reconstitution. The injection volume was 5 µL. All experiments were performed with eight biological replicates. Unpaired t-test was used for analyzing differences with* p*-values < 0.05 considered significant.

## Supplementary Information

Additional file 1.

## Data Availability

All data generated or analysed during this study are included in this published article and its supplementary information file.

## Abbreviations

AMA: Apparent molar activity
AY: Activity yield
BPin: Boronic acid pinacol
DMA: *N,N*-Dimethylacetamide
DCM: Dichloromethane
DIPEA: *N,N-*Diisopropylethylamine
FEP: Free energy perturbation
HATU: Hexafluorophosphate azabenzotriazole tetramethyl uronium
ID: Injected dose
MTBE: Methyl *tert*-butyl ether
OXR: Orexin receptor
PBS: Phosphate buffered saline
PET: Positron emission tomography
P-gp: P-glycoprotein
p.i.: Post injection
PPB: Plasma protein binding
RCY: Radiochemical yield
rt: Room temperature
TEAB: Tetraethylammonium bicarbonate
TFA: Trifluoroacetic acid
