# Supplementary material for: Heteroaryl derivatives of suvorexant as OX1R selective PET ligand candidates: Cu-mediated 18F-fluorination of boroxines, in vitro and initial in vivo evaluation
Source: EJNMMI Res. 2024 Sep 4;14:80. doi: 10.1186/s13550-024-01141-2 (PMC11374953; doi:10.1186/s13550-024-01141-2)
Supplement: Supplementary file 1 — Additional file 1. [file 13550_2024_1141_MOESM1_ESM.pdf]

Supplementary Information

Heteroaryl derivatives of suvorexant as OX1R selective PET ligand candidates: Cu-mediated ^{18}F-fluorination of boroxines, in vitro and initial in vivo evaluation

Kim-Viktoria Bolik[1], Jan Hellmann[2], Simone Maschauer[1], Eduard Neu[2,5], Jürgen Einsiedel[2], Patrick Riss[3], Nora Vogg[4], Jörg König[4,5], Martin F. Fromm[4,5], Harald Hübner[2], Peter Gmeiner[2,5], Olaf Prante[1,5]*

[1] *Department of Nuclear Medicine, Molecular Imaging and Radiochemistry, Friedrich-Alexander-Universität Erlangen-Nürnberg, Kussmaulallee 10/12, 91054 Erlangen, Germany*

[2] *Department of Chemistry and Pharmacy, Medicinal Chemistry, Friedrich-Alexander-Universität Erlangen-Nürnberg, Nikolaus-Fiebiger-Str. 10, 91058 Erlangen, Germany*

[3] *Department of Chemistry, Johannes Gutenberg-University, Fritz Strassmann Weg 2, 55128 Mainz, Germany*

[4] *Institute of Experimental and Clinical Pharmacology and Toxicology, Friedrich-Alexander-Universität Erlangen-Nürnberg, Erlangen, Germany*

[5] *FAU NeW – Research Center New Bioactive Compounds, Friedrich-Alexander-Universität Erlangen-Nürnberg, Nikolaus-Fiebiger-Str. 10, 91058 Erlangen, Germany*

***Correspondence:**
Prof. Dr. Olaf Prante
olaf.prante@uk-erlangen.de

Materials and methods

General. Human serum (Biochrom), human plasma (Hölzel Diagnostika Handels GmbH, from CosmoBio) and rat plasma (Dunn Labortechnik GmbH, from Innovative Research, Inc.) were purchased from commercial sources. All chemicals and solvents were purchased from the following companies: ABCR, ACROS Organics, Alfa Aesar, Carl Roth, Chem Bridge, Fluorochem, Life Chemicals and Sigma Aldrich. Materials were used as obtained without further purification unless noted otherwise. NMR spectra were obtained on a Bruker Avance 400 (^{1}H at 400 MHz, ^{13}C (DEPTQ) at 101 MHz, ^{19}F at 377 MHz) or a Bruker Avance 600 (^{1}H at 600 MHz, ^{13}C (DEPTQ) at 151 MHz, ^{19}F at 565 MHz) spectrometer at 298 K. Chemical shifts are reported relative to TMS (for ^{1}H and ^{13}C) or CCl_3F (for ^{19}F NMR) or the residual solvent peak. Specific optical rotation measurement was performed in a JASCO P-2000 polarimeter in methanolic solutions (path length: 100 mm, volume: 1.0 mL). Linearly polarized light was generated by a filtered sodium lamp (589 nm). The data were analyzed using the Jasco Spectra Manager software. Analytical LC-MS was performed on a Thermo Scientific Dionex Ultimate 3000 HPLC system using DAD detection (230 nm; 254 nm) equipped with either a Kinetex 2.6u mesh C8 100A (2.1 × 75 mm, 2.6 µm) HPLC column or a Zorbax Eclipse XDB-C8 (4.6 × 150 mm, 5 µm) HPLC column, using mass detection on a amaZon SL mass spectrometer (Bruker, Bellerica, MA, USA) using ESI or APCI ionization source. ESI-TOF high mass accuracy and resolution experiments were performed on an AB Sciex Triple TOF660 Sciex, on a Bruker maXis MS or a Bruker timsTOF Pro. Analytical HPLC analysis was performed on Agilent 1100 Analytical Series equipped with a VWD detector using a Zorbax Eclipse XDB-C8 column (4.6 × 150 mm, 5 µm, flow rate: 0.5 mL/min, gradient: 5% for 3 min, 5% to 95% in 15 min, 95% for 6 min, 95% to 5% in 3 min, 5% for 3 min; eluent system 1: methanol/0.1% aq. formic acid, eluent system 2: CH_3CN/0.1% aq. formic acid, eluent system 3: methanol/0.1% aq. TFA, eluent system 4: CH_3CN/0.1% aq. TFA) unless noted otherwise.

Purification by preparative RP-HPLC was performed on Agilent 1260 Preparative Series equipped with a VWD detector (230 nm; 254 nm) using a Zorbax Eclipse XDB-C8 column (21.2 × 150 mm, 5 µm, flow rate: 10 mL/min). 5-Fluorobenzoxazole (**5**) [1] and t*ert*-butyl (*S*)-2-[(*S*)-*sec*-butyl]-1,4-diazepane-1-carboxylate (**6**) [2] were prepared as described in the literature. See page 17 for the procedure to afford **6** in a solution of DMF.

Chemistry

General procedure A. A solution of the corresponding bromoheteroarene (1 eq), bis(pinacolato)-diboron (1.2 eq) and KOAc (3 eq) in dioxane (dry, 17 mM, referred to the bromoheteroarene) was prepared under argon atmosphere. [1,1′-Bis(diphenyl-phosphino)ferrocene]dichloro-palladium(II) (0.05 eq) was added and the reaction was heated to 85 °C under stirring for 1-4 days, until monitoring by LC-MS indicated full consumption of starting material. After cooling to room temperature, ethyl acetate (15 mL) was added and the solvent was removed under reduced pressure. The residue was resuspended in DCM (10 mL), washed with $NaHCO_3$ (10 mL) and brine (10 mL). Subsequently, the organic layer was dried over Na_2SO_4, filtered and the solvent was removed under reduced pressure.

General procedure B. A round neck flask connected to a Dean-Stark apparatus was charged with the corresponding boronic acid (1 eq) under argon atmosphere. Dry toluene (50 mL) was added and the reaction was heated to 105 °C under continuous stirring. The reaction was refluxed for 7 h under reduced pressure (300 mbar). Subsequently, the solvent was removed and the corresponding boroxine was obtained and stored at room temperature under an argon atmosphere.

General procedure C. The corresponding diazepane (1 eq) was dissolved in DMF with a final concentration of 50 mM. Subsequently, the corresponding 2-chloro-heteroarene (1.2 eq) and K_2CO_3 (2.5 eq) were added under continuous stirring. The reaction was stirred for 2-10 days at 60 °C, until monitoring by TLC indicated total consumption of diazepane. Subsequently, the

solvent was removed under reduced pressure, the residue was dissolved in DCM (15 mL) and washed with saturated $NaHCO_3$ solution (aq., 10 mL) and brine (10 mL), respectively. The organic layer was dried over Na_2SO_4, filtered and the solvent was removed under reduced pressure.

General procedure D. The corresponding Boc-protected amine (1 eq) was dissolved in DCM with a final concentration of 50 mM. Subsequently, TFA (10 eq) was added and the reaction was stirred at room temperature for 1-3 hours, until full deprotection was indicated by TLC. The pH was adjusted to 11 with aqueous NaOH solution and the organic layer was separated. Afterwards, the organic layer was washed with saturated $NaHCO_3$ solution (10 mL) and the aqueous layer was extracted with DCM (10 mL, 3×). The combined organic layers were dried over Na_2SO_4, filtered and the solvent was removed under reduced pressure.

Synthesis of [4-(5-fluorobenzoxazol-2-yl)-1,4-diazepan-1-yl][5-methyl-2-(2*H*-1,2,3-triazol-2-yl)phenyl]methanone (1a). 2-(1,4-Diazepan-1-yl)-5-fluorobenzoxazole (**8a**) (34.5 mg, 0.15 mmol), 5-methyl-2-(2*H*-1,2,3-triazol-2-yl)benzoic acid (35.8 mg, 0.18 mmol) and HATU (66.9 mg, 0.18 mmol) were dissolved in dimethylformamide (dry, 10 mL). DIPEA (0.13 mL, 0.73 mmol) was added and the reaction mixture was stirred at ambient temperature for 3 h. The reaction mixture was diluted with ethyl acetate and washed with saturated sodium bicarbonate solution (3×) and brine (1×). The organic layer was dried over sodium sulfate, filtered and concentrated under reduced pressure. The crude product was purified using a preparative HPLC (gradient of 5% to 95% methanol in water with 0.1% formic acid). Compound **1a** was obtained as white powder after lyophilization of the product containing fractions (31.5 mg, 0.75 µmol, 51%). ESI-MS: *m/z* 421.2 $[M+H]^+$; HR-ESI-MS: *m/z* $[M+H]^+$ calcd. 421.1783 for $C_{22}H_{21}FN_6O_2$, found 421.1778; HPLC (254 nm): eluent system 2: t_R = 17.6 and 17.9 min, rotamers, purity: 99.4%, eluent system 3: t_R = 20.6 and 20.8 min, rotamers, purity:

99.0%, eluent system 4: t_R = 17.3 and 17.6 min, rotamers, purity: 99.5%; ^{1}H NMR (400 MHz, $CDCl_3$, two sets of signals were observed, rotamers) δ 7.86 (2× d, *J* =8.3 Hz, 1H), 7.64 (s, 1H), 7.58 (s, 1H), 7.37 – 7.27 (m, 1H), 7.18 (dd, *J* = 8.7, 4.3 Hz, 0.5H), 7.15 – 7.03 (m, 1.5H), 7.04 – 6.96 (m, 1H), 6.72 (ddd, *J* = 9.5, 8.7, 2.6 Hz, 1H), 4.16 – 4.04 (m, 0.5H), 4.03 – 3.65 (m, 5.5H), 3.63 – 3.48 (m, 2H), 3.48 – 3.21 (m, 2H), 2.39 (s, 1.5H), 2.31 (s, 1.5H), 2.17 (m, 1H), 1.82 – 1.53 (m, 1H). ^{13}C NMR (DEPTQ, 151 MHz, $CDCl_3$, double set of signals due to rotamers, each set given in brackets and separated by semicolons) δ (169.4, 169.3; Cq); (160.3 (d, *J* (C,F) = 239.4 Hz), 160.2 (d, *J* (C,F) = 239.4 Hz); CF); (138.5, 138.4; Cq); (135.5, 135.5; CH); (133.6, 133.5; Cq); (130.7, 130.7; CH); (128.7, 128.6; Cq); (128.4, 128.3; CH); (122.4, 122.3; CH); (108.9, 108.9; CH); (108.8, 108.7; CH); (107.5, 107.3; CH); (103.3, 103.2; CH); (49.2, 48.8, 48.6, 48.0, 46.7, 45.6, 44.0; CH_2); (27.9, 26.5; CH_2); (20.9, 20.9; CH_3). ^{19}F NMR (377 MHz, $CDCl_3$) δ -119.30 (d, *J* = 34.9 Hz).

Synthesis of [4-(5-fluorobenzo[*d*]thiazol-2-yl)-1,4-diazepan-1-yl][5-methyl-2-(2*H*-1,2,3-triazol-2-yl)phenyl]methanone (1b). According to general procedure C, **10** (40.0 mg, 140 µmol, 1 eq) was reacted with 2-chloro-5-fluoro-benzo[*d*]thiazole (27.7 mg, 148 µmol, 1.05 eq) in DMF. The product was obtained after purification by flash column chromatography [silica, DCM:methanol (60:1)] and ensuing purification *via* semi-preparative HPLC (Kromasil C8, 125 × 10 mm, 5 µm, 4 mL/min, gradient system: CH_3CN in H_2O (0.1% TFA), 40% for 0-3 min, 40-45% in 3-11.5 min, 45-100% in 11.5-15 min) as pale green solid (4.4 mg, 10.0 µmol, 7%). Silica TLC (DCM:methanol (20:1)) R_f = 0.75; HPLC (Chromolith Performance RP-18e, 100 × 4.6 mm, 4 mL/min, 10-100% CH_3CN in H_2O (0.1% TFA) in 5 min) t_R = 2.73 min, purity: >99%; ESI-MS *m/z* 437.22 $[M+H]^+$; HRMS *m/z* $[M+H]^+$ calcd. 437.1554 for $C_{22}H_{22}FN_6OS$, found 437.1554; ^{1}H NMR (600 MHz, $CDCl_3$, double signal set observed, rotamers) *δ* 7.88 (2× d, *J* = 8.3 Hz, 1H), 7.69 (s, 1H), 7.56 – 7.53 (m, 1.5H), 7.51 – 7.45 (m, 1.1H), 7.35 – 7.32 (m, 1.3H), 7.09 (s, 0.5H), 6.98 – 6.89 (m, 1.6H), 4.23 – 4.18 (m,

0.4H), 4.05 – 3.87 (m, 4.5H), 3.72 – 3.59 (m, 1.8H), 3.57 – 3.53 (m, 0.4H), 3.47 – 3.41 (m, 0.5H), 3.34 – 3.27 (m, 0.4H), 2.38 (s, 1.5H), 2.28 (s, 1.5H), 2.27 – 2.23 (m, 1H), 1.88 – 1.70 (m, 1H). ^{13}C NMR (DEPTQ, 151 MHz, $CDCl_3$, double set of signals due to rotamers, each set given in brackets and separated by semicolons) δ (169.8, 169.6; Cq); (169.3, 168.5; Cq); (162.5 (d, *J* (C,F) = 244.9 Hz), 162.4 (d, *J* (C,F) = 244.3 Hz); CF), (138.6, 138.4; Cq); (135.5, 135.5; CH); (134.0, 133.9; Cq); (131.0, 130.9; CH); (128.2, 128.2; CH); (127.9, 127.7; Cq); (122.4, 122.4; CH); (121.8, 121.8; CH), (121.6, 121.6; CH); (111.1, 110.9; CH), (110.7, 110.5; CH); (105.3, 105.2, 105.0; CH); (52.3, 52.2, 51.9, 49.5, 49.1, 48.4, 44.9, 44.1; CH_2); (29.7, 27.3, 25.0; CH_2); (20.9, 20.7; CH_3).

Synthesis of [4-(6-Fluorobenzo[*d*]thiazol-2-yl)-1,4-diazepan-1-yl][5-methyl-2-(2*H*-1,2,3-triazol-2-yl)phenyl]methanone (1c). According to general procedure C, **10** (50 mg, 175 µmol, 1 eq) was reacted with 2-chloro-6-fluoro-benzo[*d*]thiazole (34.5 mg, 184 µmol, 1.05 eq). The product **1c** was obtained after purification by flash column chromatography [silica, DCM:methanol (30:1 (v/v), containing 1% (v/v) of 25% (w/w) ammonia solution)] as blue, green solid (44.2 mg, 101 µmol, 55%). Silica TLC (DCM:methanol (20:1 (v/v), containing 1% (v/v) of 25% (w/w) ammonia solution)), R_f = 0.48; HPLC (Chromolith Performance RP-18e, 100 × 4.6 mm, 4 mL/min, 10-100% CH_3CN in H_2O (0.1% TFA) in 5 min) t_R = 2.47 min, purity: 98.6%; ESI-MS *m/z* 437.1 $[M+H]^+$; HRMS *m/z* $[M+H]^+$ calcd. 437.1554 for $C_{22}H_{22}FN_6OS$, found 437.1554; ^{1}H NMR (600 MHz, $CDCl_3$, double signal set observed, rotamers) δ 7.86 (2× d, *J* = 8.8 Hz, 1H), 7.69 (s, 1.1H), 7.58 (s, 0.9H), 7.55 – 7.46 (m, 0.5H), 7.44 – 7.36 (m, 0.5H), 7.34 – 7.27 (m, 0.2H), 7.10 (s, 0.5H), 7.08 – 6.98 (m, 1.0H), 6.93 (s, 0.5H), 4.16 – 4.09 (m, 0.4H), 3.93 – 3.66 (m, 5H), 3.66 – 3.47 (m, 1.2H), 3.40 – 3.23 (m, 1.4H), 2.37 (s, 1.3H), 2.23 (s, 1.7H), 1.81 – 1.59 (m, 2H). ^{13}C NMR (DEPTQ, 151 MHz, $CDCl_3$, double set of signals due to rotamers, each set given in brackets and separated by semicolons) δ (169.6, 169.4; Cq); (167.2, 166.4; Cq); 158.2 (d, J (C,F) = 239.9 Hz, CF); (138.6, 138.5; Cq); (135.7, 135.6; CH);

(134.0; Cq), (130.8, 130.7; CH); (128.8, 128.6, 128.5; CH), (122.4, 122.3; CH); (119.4, 119.3; CH); (114.1, 114.0; CH); (113.9, 113.8; CH); (107.8, 107.5; m, CH); (51.5, 51.3; CH_2); (51.0, 49.1; CH_2); (48.8, 48.3: CH_2); (45.4, 44.2; CH_2); (29.8, 27.5, 25.8; CH_2); (21.1, 21.0; CH_3).

Synthesis of [4-(6-fluoroquinoxalin-2-yl)-1,4-diazepan-1-yl][5-methyl-2-(2*H*-1,2,3-triazol-2-yl)phenyl]methanone (1d). According to general procedure C, **10** (40.0 mg, 140 µmol, 1 eq) was reacted with 2-chloro-6-fluoro-quinoxaline (26.9 mg, 147 µmol, 1.05 eq). Compound **1d** was obtained after purification by flash column chromatography [silica, DCM:methanol (40:1 (v/v), containing 1% (v/v) of 25% (w/w) ammonia solution)], resuspension in aqueous methanolic solution (40%, 3 mL) and lyophilization as white yellowish powder (22.6 mg, 46.4 mmol, 33%). Silica TLC (DCM:methanol (20:1)) R_f = 0.63; HPLC (Chromolith Performance RP-18e, 100 × 4.6 mm, 4 mL/min, 10-100% CH_3CN in H_2O (0.1% TFA) in 5 min) t_R = 2.91 min, purity: 98.7%; ESI-MS *m/z* 432.64 $[M+H]^+$; HRMS *m/z* $[M+H]^+$ calcd. 432.1943 for $C_{23}H_{23}FN_7O$, found 432.1943; ^1H NMR (600 MHz, $CDCl_3$, double signal set observed, rotamers) δ 8.54 – 8.44 (m, 1H), 7.86 (d, *J* = 8.3 Hz, 0.4H), 7.82 (d, *J* = 8.2 Hz, 0.6H), 7.79 – 7.72 (m, 0.4H), 7.69 – 7.64 (m, 1.2H), 7.61 – 7.50 (m, 0.2H), 7.41 – 7.30 (m, 1.4H), 7.27 (s, 1H), 7.12 – 7.07 (m, 0.4H), 6.77 – 6.71 (m, 0.6H), 4.16 – 4.04 (m, 2.2H), 4.00 – 3.88 (m, 1.2H), 3.85 – 3.73 (m, 1.3H), 3.68 – 3.56 (m, 1.9H), 3.35 – 3.19 (m, 1.4H), 2.38 (s, 1H), 2.38 (s, 1H), 2.25 – 2.19 (m, 0.6H), 2.14 (s, 2H), 1.79 – 1.71 (m, 0.6H), 1.67 – 1.57 (m, 0.8H). ^{13}C NMR (DEPTQ, 151 MHz, $CDCl_3$, double set of signals due to rotamers, each set given in brackets and separated by semicolons) δ (169.5, 169.5; Cq); 159.7 (d, *J* (C,F) = 245.2 Hz; CF); (150.5, 150.4; Cq); 149.94, 149.1; Cq); (138.6, 138.5; Cq); (136.6, 136.5; Cq); (136.0, 135.7; CH); (135.6, 135.6; CH); (134.1, 134.1; Cq), (130.8, 130.7; CH), (128.9, 128.8; Cq); (128.6, 128.5; CH); (127.7, 127.7; CH); (127.5, 127.4; CH); (122.5, 122.4; CH), (120.2 (m), CH); (112.8, 112.7; CH); (48.9 - 44.2; CH_2); (45.7, 44.2; CH_2); (27.4, 26.1; CH_2); (21.1, 20.9; CH_3).

Synthesis of {(*S*)-2-[(*S*)-*sec*-butyl]-4-(5-fluorobenzoxazol-2-yl)-1,4-diazepan-1-yl} [5-methyl-2-(2*H*-1,2,3-triazol-2-yl)phenyl]methanone (1e). 2-{(*S*)-3-[(*S*)-*sec*-butyl]-1,4-diazepan-1-yl}-5-fluorobenzoxazole (**8b**) (13.3 mg, 45.7 µmol), 5-methyl-2-(2*H*-1,2,3-triazol-2-yl)benzoic acid (11.1 mg, 54.8 µmol) and HATU (20.8 mg, 54.8 µmol) were dissolved in dimethylformamide (dry, 2 mL). DIPEA (39.5 µL, 0.23 mmol) was added and the reaction mixture was stirred at ambient temperature for 24 h. The reaction mixture was diluted with ethyl acetate and washed with saturated aqueous sodium bicarbonate solution (3×) and brine (1×). The organic layer was dried over sodium sulfate, filtered and concentrated under reduced pressure. The crude product was purified using a preparative HPLC (gradient of 5% to 95% methanol in water with 0.1% formic acid). Compound **1e** was obtained as white powder after lyophilization (6.5 mg, 13.6 µmol, 30%). ESI-MS: *m/z* 477.2 $[M+H]^+$; HR-ESI-MS: *m/z* [M+H]+ calcd. 477.2409 for $C_{26}H_{29}FN_6O_2$, found 477.2410; HPLC (254 nm): eluent system 1: t_R = 21.7 and 21.9 min, rotamers, purity: 98.7%, eluent system 2: t_R = 20.0 and 20.1 min, rotamers, purity: 98.1%, eluent system 3: t_R = 22.3 and 22.5 min, rotamers, purity: 98.7%, eluent system 4: t_R = 21.7 and 21.9 min, rotamers, purity: 98.9%; $[\alpha]_D$: + 160.6° (c = 0.15 in methanol); ^{1}H NMR (600 MHz, $CDCl_3$, four sets of signals were observed, rotamers) δ 7.90, 7.88 and 7.81 (3× d, J_1 = 8.8 Hz, J_2 = 8.0 Hz, J_3 = 8.3 Hz, 1H), 7.75 and 7.74 (2× s, 1H), 7.44 (s, 0.5H), 7.30 and 7.28 (2× dd, each *J* = 7.9, 1.2 Hz, 1H), 7.24 (s, 0.5H), 7.21 – 7.12 (m, 1.2H), 7.08 – 7.00 (m, 1.2H), 6.77 – 6.70 (m, 0.7H), 6.67 (ddd, *J* = 9.4, 8.8, 2.6 Hz, 0.3H), 6.35 (s, 0.1H), 4.97 – 4.80 (m, 0.5H), 4.77 (ddd, *J* = 14.0, 3.5, 3.5 Hz, 0.2H), 4.63 (ddd, *J* = 13.9, 3.2, 3.2 Hz, 0.2H), 4.40 (dd, *J* = 14.5, 5.1 Hz, 0.2H), 4.29 – 4.18 (m, 0.9H), 4.18 – 4.08 (m, 0.3H), 4.04 (dd, *J* = 15.0, 4.4 Hz, 0.2H), 3.98 (ddd, *J* = 14.1, 4.8, 4.8 Hz, 0.3H), 3.90 (dd, *J* = 15.0, 8.3 Hz, 0.3H), 3.85 (ddd, *J* = 15.4, 4.2, 4.2 Hz, 0.2H), 3.72 – 3.60 (m, 0.5H), 3.60 – 3.34 (m, 1.4H), 3.69 (dd, *J* = 14.5, 4.3 Hz, 0.2H), 3.67 – 3.60 (m, 0.2H), 3.60 – 3.34 (m, 1.4H), 3.32 – 3.15 (m, 1.2H), 3.12 – 2.99 (m, 0.4H), 2.41, 2.40, 2.36 and 1.80 (4× s, 3H), 2.38 – 2.28 (m, 0.5H), 2.09 – 1.89

(m, 1.2H), 1.88 – 1.80 (m, 0.9H), 1.76 – 1.60 (m, 0.8H), 1.36 – 1.19 (m, 1.6H), 1.10, 1.02, 0.98, and 0.78 (4× d, $J_1 = J_2$ = 6.8 Hz, $J_3 = J_4$ = 6.6 Hz, 3H), 1.02, 1.01, 0.67, and 0.55 (4× t, $J_1 = J_2 = J_3$ = 7.4 Hz, J_4 = 7.1 Hz, 3H).

^{13}C NMR (DEPTQ, 151 MHz, $CDCl_3$, quadruple set of signals due to rotamers, each set separated by semicolons) δ 170.4, 170.2, 170.0 and 169.8 (Cq, N**C**=O); 163.1, 162.9, 162.8 and 162.5 (C-2 benzoxazole); 160.3 (d, J = 237 Hz), 160.2 and 160.1 (2× d, for each J = 238 Hz, C-5 benzoxazole); 145.3, 145.2, 145.1, 144.9, 144.7, 144.64, 144.55 and 144.37 (C-3a and C-7a benzoxazole); 138.4, 138.1, 138.0 and 137.4 (**C**CH_3 benzamide); 135.5 and 135.4 (**C**H triazole); 135.3 and 135.1 (**C**H triazole); 134.1, 133.6, 133.4 and 133.3 (O=C-**C** benzamide); 130.4, 130.34, 130.30 and 130.2 (CH_3-C-**C**H-CH benzamide); 129.9, 129.1 and 128.18 (**C**-N benzamide); 129.7, 128.9; 128.3 and 128.22 (**C**H-C-CH_3 benzamide); 122.6, 122.2, 121.9 and 121.5 (N-C-**C**H benzamide); 108.8, 108.66, 108.65 (3× d, for each J = 10 Hz) and 107.0 (d, J = 11 Hz, C-7 benzoxazole); 107.0, 106.8 and 106.6 (3× d, for each J = 26 Hz, C-6 benzoxazole); 103.6, 103.31, 103.25 and 103.17 (4× d, for each J = 27 Hz, C-4 benzoxazole); 59.7, 58.3, 56.6, 56.5 (**C**H of diazepane); 50.7, 50.0, 49.5, 49.3, 49.1, 49.0, 48.5 and 48.1 (2× N-**C**H_2 of diazepane); 45.3, 44.4, 42.2 and 41.3 (O=C-N-**C**H_2 of diazepane); 37.4, 37.4, 35.4 and 34.8 (**C**H *sec*-butyl); 28.4, 27.8, 27.4, 26.9, 26.7, 26.4, 26.2 and 25.9 (CH_2-**C**H_2-CH_2 of diazepane and **C**H_2 *sec*-butyl); 21.01, 20.99, 20.9 and 20.3 (**C**H_3 benzamide); 15.1, 14.7, 14.6 and 14.1 (CH-**C**H_3); 11.74, 11.67, 11.4 and 11.3 (CH_2-**C**H_3). ^{19}F NMR (565 MHz, $CDCl_3$) δ -119.39 - -119.69 (m).

Synthesis of {(*S*)-2-[(*S*)-*sec*-butyl]-4-(6-fluorobenzo[*d*]thiazol-2-yl)-1,4-diazepan-1-yl}[5-methyl-2-(2*H*-1,2,3-triazol-2-yl)phenyl]methanone (1f). A solution of **9a** (26.0 mg, 84.6 µmol, 1 eq) in DMF (28 mM) together with commercially available 5-methyl-2-(2*H*-1,2,3-triazol-2-yl)benzoic acid (1.2 eq) and HATU (1.2 eq) was prepared. Under continuous stirring, DIPEA (5 eq) added dropwise. The reaction was stirred overnight and the solvent was removed

under reduced pressure. Subsequently, the residue was dissolved in DCM (15 mL) and washed with saturated $NaHCO_3$ solution (10 mL) and brine (10 mL). The organic layer was dried over Na_2SO_4, filtered and the solvent was removed under reduced pressure. Compound **1f** was obtained after purification by semi-preparative HPLC (Kromasil C8, 125 × 10 mm, 5 µm, 4 mL/min, gradient system: CH_3CN in H_2O (0.1% TFA), 40% in 0-3 min, 40-45% in 3-11.5 min, 45-70% in 11.5-17 min) as brown yellowish oil (30.1 mg, 97.9 µmol, 49%). Silica TLC (DCM:methanol (50:1 (v/v), containing 1% (v/v) of 25% (w/w) ammonia solution)) R_f = 0.44; HPLC (Chromolith Performance RP-18e, 100 × 4.6 mm, 4 mL/min, 10-100% CH_3CN in H_2O (0.1% TFA) in 5 min) t_R = 3.16 and 3.27 min (mixture of rotamers), purity: 98.8%; $[\alpha]^{24}_{D}$: +54.8 (3.32 mM in methanol); ESI-MS *m/z* 493.3 $[M+H]^+$; HRMS *m/z* $[M+H]^+$ calcd. 493.2180 for $C_{26}H_{29}FN_6OS$, found 493.2180; ^1H NMR (600 MHz, $CDCl_3$, four sets of signals (plus fluoro couplings) were observed, rotamers) δ 7.91 – 7.89 (m, 0.3H), 7.88 (s, 0.2H), 7.87 (s, 0.1H), 7.81 (s, 0.2H), 7.80 (s, 0.2H), 7.74 (s, 0.5H), 7.72 (s, 0.6H), 7.59 – 7.48 (m, 0.5H), 7.43 (s, 0.5H), 7.40 (dd, J = 8.7, 4.7 Hz, 0.4H), 7.37 – 7.25 (m, 2.2H), 7.23 (dd, J = 8.1, 2.5 Hz, 0.2H), 7.18 – 7.11 (m, 0.4H), 7.08 – 6.99 (m, 1.2H), 6.88 (s, 0.3H), 6.27 (s, 0.2H), 5.04 – 4.87 (m, 0.4H), 4.81 – 4.75 (m, 0.2H), 4.66 – 4.61 (m, 0.2H), 4.33 – 4.27 (m, 0.4H), 4.24 – 4.17 (m, 0.3H), 4.14 – 4.00 (m, 0.6H), 3.97 (d, J = 6.6 Hz, 0.4H), 3.86 – 3.76 (m, 0.4H), 3.67 – 3.62 (m, 0.2H), 3.59 – 3.34 (m, 0.8H), 3.37 – 3.30 (m, 0.5H), 3.29 – 3.17 (m, 0.8H), 3.12 (t, J = 12.7 Hz, 0.2H), 3.04 (t, J = 13.1, 12.6 Hz, 0.2H), 2.43 – 2.28 (m, 2.8H), 2.07 – 2.01 (m, 0.6H), 2.00 – 1.94 (m, 0.4H), 1.92 – 1.79 (m, 1H), 1.77 – 1.71 (m, 0.5H), 1.69 – 1.56 (m, 1.7H), 1.54 – 1.48 (m, 0.3H), 1.34 – 1.20 (m, 1.8H), 1.10 (d, J = 6.7 Hz, 0.6H), 1.02 (t, J = 7.3 Hz, 2H), 0.99 (d, J = 6.8 Hz, 1H), 0.90 – 0.81 (m, 0.7H), 0.79 (d, J = 6.7 Hz, 0.7H), 0.67 (t, J = 7.4 Hz, 0.6H), 0.56 (t, J = 7.2 Hz, 0.7H). ^{13}C NMR (DEPTQ, 151 MHz, $CDCl_3$, quadruple set of signals due to rotamers, each set separated by semicolons): δ (ppm) 169.9, 169.8, 169.7 and 167.3; 166.5, 166.4, 165.9 and 165.2; 157.7 and 157.5 (2× d, for each J = 239 Hz); 149.1; 138.0, 137.7,

137.5 and 137.0; 135.1 and 135.0; 134.9 and 134.8; 133.8, 133.1, 133.0 and 132.8; 130.9 and 130.8; 129.99, 129.97, 129.87, 129.6, 129.35, 128.7, 128.1 and 127.9; 127.73 and 127.68; 122.2, 121.7, 121.2 and 121.1; 119.1 (d, J = 8 Hz), 118.84 (d, J = 9 Hz), 118.75 (d, J = 9 Hz) and 118.6 (d, J = 8 Hz); 113.3 (d, J = 24 Hz); 107.24 (d, J = 28 Hz), 107.17 (d, J = 28 Hz); 107.13 (d, J = 27 Hz) and 106.0 (d, J = 27 Hz); 58.8, 56.4 and 54.7; 53.4, 52.0, 51.7 and 50.4; 45.3, 44.2, 42.0 and 40.9; 37.1, 37.0, 35.4 and 34.5; 27.5, 27.1, 26.7 and 26.3; 26.2, 26.1, 25.9 and 25.5; 20.6, 20.5 and 19.8; 14.9, 14.42, 14.37 and 13.74; 11.4, 11.3, 11.0 and 10.9.

Synthesis of [4-(5-bromobenzo[*d*]oxazol-2-yl)-1,4-diazepan-1-yl][5-methyl-2-(2*H*-1,2,3-triazol-2-yl)phenyl]methanone (2a). Compound **10** (193 mg, 677 µmol, 1 eq), 5-bromobenzoxazole (148 mg, 745 µmol, 1.1 eq) and copper(II) acetate hydrate (122 mg, 609 µmol, 0.9 eq) were dissolved in CH_3CN (18 mL). Under continuous stirring the dropwise addition of concentrated acetic acid (77 µL, 1.35 mmol, 2 eq) followed and the reaction was refluxed at 75 °C over 3 days. After cooling to room temperature the solvent was removed under reduced pressure and the residue was resuspended in ethyl acetate (30 mL). The organic phase was washed with saturated $NaHCO_3$ solution (10 mL, 3×). The organic layer was dried over Na_2SO_4, filtered and the solvent was removed under reduced pressure. Compound **2a** was obtained after purification by flash column chromatography [silica, DCM:methanol (20:1 (v/v), containing 1% (v/v) of 25% (w/w) ammonia solution)] as brown oil (217 mg, 451 µmol, 67%). Silica TLC (DCM:methanol (10:1)) R_f = 0.43; ESI-MS *m/z* 483.2 $[M+H]^+$; ^{1}H NMR (600 MHz, $CDCl_3$, double signal set observed - rotamers) δ 7.86 (dd, J = 14.4, 8.3 Hz, 1H), 7.64 (s, 1.1H), 7.61 – 7.55 (m, 1.3H), 7.52 (s, 0.5H), 7.34 – 7.29 (m, 1.1H), 7.24 – 7.21 (m, 0.5H), 7.20 – 7.17 (m, 0.9H), 7.14 – 7.07 (m, 1H), 7.04 – 6.96 (m, 0.6H), 4.16 – 3.24 (m, 8H), 2.39 (s, 1.4H), 2.33 (s, 1.6H), 2.25 – 2.12 (m, 1H), 1.84 – 1.68 (m, 1H). ^{13}C NMR (151 MHz, $CDCl_3$, double signal set observed - rotamers) δ 169.4, 146.7, 146.7, 138.6, 138.5, 138.5, 138.4, 135.5, 135.5, 134.1, 134.0, 130.9, 130.8, 128.6, 128.2, 124.8, 124.7, 122.5, 122.5, 122.4, 118.6, 117.9, 117.8, 110.5,

110.3, 49.8, 49.2, 48.8, 48.8, 48.4, 47.2, 45.3, 44.0, 29.7, 28.0, 26.2, 21.0, 20.9.

Synthesis of [4-(6-bromobenzo[*d*]thiazol-2-yl)-1,4-diazepan-1-yl][5-methyl-2-(2*H*-1,2,3-triazol-2-yl)phenyl]methanone (2c). According to general procedure C, **10** (500 mg, 1.75 mmol, 1 eq) was reacted with 6-bromo-2-chloro-benzo[*d*]thiazole (457 mg, 1.84 mmol, 1.05 eq). Compound **2c** was obtained after purification by flash column chromatography [silica, DCM:methanol (30:1 to 10:1 (v/v), containing 1% (v/v) of 25% (w/w) ammonia solution)] as yellowish brown solid (612 mg, 1.23 mmol, 70%). Silica TLC (DCM:methanol (20:1)) R_f = 0.56; ESI-MS *m/z* 499.0 $[M+H]^+$; ^{1}H NMR (600 MHz, $CDCl_3$, double signal set observed - rotamers) δ 7.86 (t, J = 17.5, 8.5 Hz, 1H), 7.72 (d, J = 2.0 Hz, 0.5H), 7.69 – 7.67 (m, 1.6H), 7.56 (s, 1H), 7.50 – 7.34 (m, 1.9H), 7.32 – 7.28 (m, 1.1H), 7.10 (d, J = 1.1 Hz, 0.5H), 6.94 (d, J = 1.0 Hz, 0.5H), 4.19 – 3.22 (m, 8H), 2.37 (s, 1.3H), 2.25 (s, 1.7H), 2.23 – 2.21 (m, 0.3H), 2.20 – 2.12 (m, 0.7H), 1.79 – 1.73 (m, 0.2H), 1.68 – 1.61 (m, 0.4H). ^{13}C NMR (151 MHz, $CDCl_3$, double signal set observed - rotamers) δ 169.5, 169.4, 167.8, 167.0, 162.7, 138.6, 138.5, 135.7, 135.6, 134.1, 130.8, 130.8, 129.7, 129.6, 128.8, 128.6, 128.5, 123.4, 123.4, 122.4, 122.4, 120.0, 113.9, 77.4, 77.2, 77.0, 51.6, 51.2, 49.1, 49.0, 48.4, 45.3, 44.2, 38.8, 36.6, 31.6, 27.6, 25.7, 21.1, 21.0.

Synthesis of [4-(6-bromoquinoxalin-2-yl)-1,4-diazepan-1-yl][5-methyl-2-(2*H*-1,2,3-triazol-2-yl)phenyl]methanone (2d). According to general procedure C, **10** (360 mg, 1.26 mmol, 1 eq) was reacted with 6-bromo-2-chloro-quinoxaline (322 mg, 1.32 mmol, 1.05 eq). Compound **2d** was obtained after purification by flash column chromatography [silica, DCM:methanol (60:1 to 20:1 (v/v), containing 1% (v/v) of 25% (w/w) ammonia solution)] as bright yellow solid (406 mg, 74.0 mmol, 59%). Silica TLC (DCM:methanol (20:1)) R_f = 0.65; ESI-MS *m/z* 494.12 $[M+H]^+$; ^{1}H NMR (600 MHz, $CDCl_3$, double signal set observed - rotamers) δ 8.50 (s, 0.4H), 8.45 (s, 0.6H), 8.08 – 8.03 (m, 1H), 7.86 (d, J = 8.4 Hz, 0.6H), 7.81 (d, J = 8.4 Hz, 0.7H), 7.69 – 7.65 (m, 1.9H), 7.62 (dd, J = 8.9, 2.2 Hz, 0.5H), 7.56 – 7.50 (m,

0.5H), 7.50 (s, 0.8H), 7.38 – 7.30 (m, 0.6H), 7.29 – 7.25 (m, 0.5H), 7.12 – 7.08 (m, 0.3H), 6.75 (s, 0.6H), 4.17 – 4.05 (m, 2H), 4.04 – 3.79 (m, 1.9H), 3.76 – 3.56 (m, 2.7H), 3.36 – 3.19 (m, 1.4H), 2.37 (s, 1.1H), 2.25 – 2.16 (m, 0.5H), 2.15 (s, 1.9H), 1.78 – 1.56 (m, 1H). ^{13}C NMR (151 MHz, $CDCl_3$, double signal set observed - rotamers) δ 169.5, 138.6, 138.5, 137.1, 135.9, 135.6, 134.1, 134.1, 133.8, 133.8, 131.1, 130.8, 130.7, 128.9, 128.7, 128.5, 128.46, 127.5, 127.3, 122.5, 122.4, 117.5, 117.5, 49.0, 48.7, 48.4, 48.1, 47.9, 46.0, 45.7, 44.2, 27.4, 26.0, 21.1, 20.9.

Synthesis of {(*S*)-2-[(*S*)-*sec*-butyl]-4-(6-bromobenzo[*d*]thiazol-2-yl)-1,4-diazepan-1-yl}[5-methyl-2-(2*H*-1,2,3-triazol-2-yl)phenyl]methanone (2e). Compound **9b** (402 mg, 1.09 mmol, 1 eq) was coupled with 5-methyl-2-(2*H*-1,2,3-triazol-2-yl)benzoic acid according to the procedure for **1f**, to give **2e**. Compound **2e** was obtained after purification by flash column chromatography [RP-C18, 10-100% CH_3CN in H_2O, 18 mL/min] as yellow brown solid (291 mg, 526 µmol, 48%). Silica TLC (DCM:methanol (199:1 (v/v), containing 1% (v/v) of 25% (w/w) ammonia solution)) R_f = 0.16; ESI-MS *m/z* 555.1 $[M+H]^+$; ^{1}H NMR (600 MHz, $CDCl_3$, four sets of signals were observed, rotamers) δ 8.23 (d, *J* = 8.9 Hz, 0.6H), 7.94 – 7.86 (m, 1.0H), 7.82 (d, *J* = 8.3 Hz, 0.2H), 7.75 – 7.71 (m, 1.0H), 7.63 – 7.61 (m, 0.1H), 7.57 (s, 1.0H), 7.52 (d, *J* = 2.1 Hz, 0.3H), 7.51 (d, *J* = 2.1 Hz, 0.3H), 7.49 (d, *J* = 2.0 Hz, 0.6H), 7.46 – 7.45 (m, 0.3H), 7.45 – 7.43 (m, 0.8H), 7.42 – 7.37 (m, 0.3H), 7.36 – 7.35 (m, 0.6H), 7.32 – 7.29 (m, 0.2H), 7.29 – 7.26 (m, 0.3H), 7.17 – 7.16 (m, 0.2H), 7.03 (s, 0.1H), 6.88 (s, 0.1H), 5.03 – 4.85 (m, 0.2H), 4.83 – 4.75 (m, 0.1H), 4.67 – 4.62 (m, 0.1H), 4.36 – 4.16 (m, 0.5H), 4.16 – 4.09 (m, 0.3H), 4.08 – 4.01 (m, 0.3H), 4.00 – 3.93 (m, 0.3H), 3.92 – 3.83 (m, 0.2H), 3.82 – 3.75 (m, 0.1H), 3.70 – 3.65 (m, 0.2H), 3.63 – 3.58 (m, 0.2H), 3.58 – 3.40 (m, 0.9H), 3.40 – 3.18 (m, 1.1H), 3.17 – 3.09 (m, 0.3H), 3.08 – 3.00 (m, 0.2H), 2.99 – 2.97 (m, 0.1H), 2.90 (s, 0.1H), 2.46 (s, 1.9H), 2.42 – 2.28 (m, 2.2H), 2.12 – 1.94 (m, 0.5H), 1.92 – 1.80 (m, 0.2H), 1.77 – 1.71 (m, 0.2H), 1.68 – 1.47 (m, 1.5H), 1.46 – 1.39 (m, 0.8H), 1.37 (t, *J* = 7.3 Hz, 0.3H), 1.34 – 1.13 (m, 2.3H), 1.12 – 1.05 (m, 0.9H), 1.05 – 0.94 (m, 3H), 0.92 – 0.81 (m, 0.9H), 0.79 (d, *J* = 6.7 Hz,

0.4H), 0.78 – 0.71 (m, 0.2H), 0.67 (t, J = 7.4 Hz, 0.4H), 0.56 (t, *J* = 7.2 Hz, 0.5H).

Synthesis of [5-methyl-2-(2*H*-1,2,3-triazol-2-yl)phenyl]{4-[5-(4,4,5,5-tetramethyl-1,3,2-dioxaborolan-2-yl)benzo[*d*]oxazol-2-yl]-1,4-diazepan-1-yl}methanone (3a). According to general procedure A, **2a** (208 mg, 434 µmol, 1 eq) was converted into the corresponding boronic acid pinacol ester **3a**. Compound **3a** was obtained after purification by flash column chromatography [silica, *n*-hexane:ethyl acetate (4:1)] as red brown solid (200 mg, 416 µmol, 95%). Silica TLC (*n*-hexane:ethyl acetate (2:1)) R_f = 0.78; ESI-MS *m/z* 529.3 $[M+H]^+$; ^{1}H NMR (600 MHz, $CDCl_3$, double signal set observed - rotamers) δ 7.89 – 7.81 (m, 1.5H), 7.77 (s, 0.5H), 7.67 – 7.51 (m, 3H), 7.36 – 7.19 (m, 2H), 7.16 – 6.97 (m, 1H), 4.22 – 3.24 (m, 8H), 3.05 – 3.00 (m, 0.4H), 2.39 (s, 1.4H), 2.31 (s, 1.6H), 2.25 – 2.14 (m, 1H), 1.79 – 1.60 (m, 1H), 1.38 – 1.18 (m, 9.3H). ^{13}C NMR (151 MHz, $CDCl_3$, double signal set observed - rotamers) δ 169.7, 169.6, 161.3, 161.1, 150.7, 150.6, 141.2, 140.9, 138.7, 138.7, 138.6, 135.7, 135.6, 134.2, 134.1, 130.9, 128.8, 128.6, 128.5, 128.5, 128.4, 128.3, 122.5, 122.5, 122.4, 122.3, 120.8, 120.7, 108.8, 108.6, 84.0, 84.0, 49.3, 49.0, 48.8, 48.2, 47.0, 46.8, 45.7, 45.4, 44.2, 44.2, 28.0, 26.7, 26.5, 25.0, 25.0, 21.1, 21.0.

Synthesis of [5-methyl-2-(2*H*-1,2,3-triazol-2-yl)phenyl]{4-[6-(4,4,5,5-tetramethyl-1,3,2-dioxaborolan-2-yl)benzo[*d*]thiazol-2-yl]-1,4-diazepan-1-yl}methanone (3c). According to general procedure A, **2c** (601 mg, 1.21 mmol, 1 eq) was converted into the corresponding boronic acid pinacol ester **3c**. After purification by flash column chromatography [silica, DCM:methanol (80:1)], **3c** was obtained as pale yellow solid (650 mg, 1.20 mmol, 99%). Silica TLC (DCM:methanol (20:1 (v/v), containing 1% (v/v) of 25% (w/w) ammonia solution)) R_f = 0.56; HPLC (Chromolith Performance RP-18e, 100 × 4.6 mm, 4 mL/min, 10-100% CH_3CN in H_2O (0.1% TFA) in 5 min) t_R = 3.15 min; ESI-MS *m/z* 545.2 $[M+H]^+$; ^{1}H NMR (600 MHz, $CDCl_3$, double signal set observed - rotamers) δ 8.08 (s, 0.5H), 8.04 (s, 0.5H), 7.88 – 7.83 (m, 1H), 7.89 – 7.83 (m, 0.5H), 7.75 (d, *J* = 6.9 Hz, 0.5H), 7.68 (s, 1H), 7.67 – 7.56 (m, 0.7H), 7.53

(s, 1H), 7.51 – 7.42 (m, 0.3H), 7.30 (t, J = 9.1 Hz, 1H), 7.12 (s, 0.5H), 6.97 (s, 0.5H), 4.39 – 4.11 (m, 0.9H), 4.08 – 3.76 (m, 3.7H), 3.74 – 3.54 (m, 1.8H), 3.53 – 3.36 (m, 1H), 3.36 – 3.15 (m, 0.5H), 2.38 (s, 1.3H), 2.25 (s, 1.7H), 1.94 (s, 1H), 1.80 – 1.79 (m, 0.1H), 1.70 – 1.62 (m, 1H), 1.36 (s, 4.9H), 1.35 (s, 6.5H). Ratio of 31 to 31.1 (corresponding boronic acid) was 95:5 as calculated from measured ^{1}H NMR. ^{13}C NMR (151 MHz, $CDCl_3$, double signal set observed - rotamers) δ 169.6, 169.5, 168.8, 168.1, 138.7, 138.5, 135.8, 135.8, 134.1, 130.9, 130.8, 128.8, 128.6, 128.6, 128.5, 127.9, 127.8, 127.7, 122.5, 84.1, 84.0, 49.4, 49.4, 44.4, 32.1, 31.9, 29.8, 29.8, 25.0, 25.0, 25.0, 21.2, 21.1.

Synthesis of [5-methyl-2-(2*H*-1,2,3-triazol-2-yl)phenyl]{4-[6-(4,4,5,5-tetramethyl-1,3,2-dioxaborolan-2-yl)quinoxalin-2-yl]-1,4-diazepan-1-yl}methanone (3d). According to general procedure A, **2d** (245 mg, 805 µmol, 1 eq) was converted into the corresponding boronic acid pinacol ester **3d**. Compound **3d** was obtained after purification by flash column chromatography [silica, DCM:methanol (60:1 to 20:1 (v/v), containing 1% (v/v) of 25% (w/w) ammonia solution)] as light beige solid (262 mg, 439 µmol, 55%). Silica TLC (DCM:methanol (50:1 (v/v), containing 1% (v/v) of 25% (w/w) ammonia solution)) R_f = 0.57; ESI-MS *m/z* 540.36 $[M+H]^+$; ^{1}H NMR (600 MHz, $CDCl_3$, double signal set observed - rotamers) δ 8.63 – 8.46 (m, 0.8H), 8.40 (s, 0.5H), 8.36 (s, 0.6H), 7.97 (s, 0.5H), 7.92 (s, 0.7H), 7.85 (d, J = 7.1 Hz, 0.8H), 7.81 (d, J = 6.5 Hz, 0.9H), 7.65 (s, 1.3H), 7.61 (d, J = 7.8 Hz, 0.4H), 7.54 (s, 0.2H), 7.47 (s, 0.8H), 7.37 (d, J = 7.8 Hz, 0.1H), 7.31 (d, J = 7.1 Hz, 0.9H), 7.21 – 7.06 (m, 0.2H), 6.80 (s, 0.3H), 4.60 – 4.35 (m, 0.6H), 4.33 – 3.87 (m, 3.4H), 3.84 – 3.55 (m, 2.8H), 3.46 – 3.24 (m, 1.2H), 2.39 (s, 1H), 2.26 – 2.19 (m, 0.9H), 2.15 (s, 2H), 1.99 – 1.94 (m, 1.1H), 1.38 (s, 12H). ^{13}C NMR (151 MHz, $CDCl_3$, double signal set observed - rotamers) δ 169.6, 150.9, 138.7, 138.5, 136.7, 136.6, 136.1, 134.1, 134.0, 131.9, 131.1, 131.0, 130.4, 130.3, 128.8, 128.7, 128.6, 128.5, 128.5, 124.9, 122.6, 122.5, 84.1, 84.0, 58.5, 53.2, 51.6, 51.6, 50.6, 50.6, 42.7, 42.7, 31.9, 31.9, 29.7, 29.7, 25.0, 25.0, 21.4, 21.2.

Synthesis of {[(1,3,5,2,4,6-trioxatriborinane-2,4,6-triyl)tris(benzo[*d*]thiazole-6,2-diyl)]-tris(1,4-diazepane-4,1-diyl)}tris{[5-methyl-2-(2*H*-1,2,3-triazol-2-yl)phenyl]methanone} (4c). First, pinacol ester **3c** was converted into the corresponding boronic acid, (2-{4-[5-methyl-2-(2*H*-1,2,3-triazol-2-yl)benzoyl]-1,4-diazepan-1-yl}benzo[*d*]thiazol-6-yl)boronic acid. Compound **3c** (51.4 mg, 94.4 µmol, 1 eq) was dissolved in acetone (1 mL) and methylboronic acid (10 eq) and aqueous NaOH solution (0.1 M, 1 mL) were added under continuous stirring. The reaction was stirred at room temperature overnight and neutralized to pH 7 by the addition of aqueous HCl (0.1 M). Subsequently, all volatiles were coevaporated with H_2O (5 mL, 3×) at 40 °C under reduced pressure. The residue was resuspended in acetone (10 mL), filtered and the solvent was removed under reduced pressure to obtain the desired boronic acid as white yellowish solid (40.6 mg, 87.8 µmol) in 93% yield, which was further converted into boroxine **4c** following the general procedure B. Compound **4c** was obtained as light brown solid (0.39 mg, 29.3 µmol) in 99% yield. Silica TLC (DCM:methanol (20:1 (v/v), containing 1% (v/v) of 25% (w/w) ammonia solution)) R_f = 0.56; ESI-MS (boronic acid intermediate) *m/z* 463.1 $[M+H]^+$; ^{1}H NMR boronic acid intermediate: (600 MHz, $CDCl_3$, double signal set observed - rotamers) 8.55 – 8.47 (m, 0.1H), 8.25 – 8.19 (m, 0.1H), 8.08 – 8.01 (m, 0.2H), 7.90 – 7.82 (m, 0.9H), 7.73 – 7.67 (m, 1.6H), 7.63 (dd, *J* = 16.6, 8.0 Hz, 0.3H), 7.60 – 7.50 (m, 1.7H), 7.49 – 7.40 (m, 0.4H), 7.36 – 7.28 (m, 1.4H), 7.16 – 7.05 (m, 0.8H), 6.97 – 6.90 (m, 0.4H), 6.84 – 6.75 (m, 0.1H), 4.26 – 4.17 (m, 1.4H), 4.16 – 4.09 (m, 0.2H), 4.02 – 3.58 (m, 4.9H), 3.58 – 3.45 (m, 0.4H), 3.43 – 3.20 (m, 1.1H), 2.37 (s, 1.3H), 2.21 (s, 1.7H), 2.17 – 1.97 (m, 0.8H), 1.82 – 1.71 (m, 1.2H). ^{13}C NMR boronic acid intermediate: (151 MHz, $CDCl_3$, double signal set observed - rotamers) δ 169.7, 169.5, 167.9, 167.7, 138.7, 138.7, 138.5, 135.8, 135.7, 134.1, 134.0, 132.6, 131.0, 130.8, 130.7, 129.0, 128.9, 128.9, 128.7, 128.5, 126.8, 126.8, 126.3, 126.2, 122.4, 122.3, 120.9, 120.9, 51.0, 50.9, 49.2, 49.1, 45.6, 45.5, 44.3, 44.2, 38.9, 23.9, 23.1, 21.1, 20.9. MALDI-TOF **4c**: *m/z* $[M+H]^+$ calcd. 1333.4692 for $C_{66}H_{64}B_3N_{18}O_6S_3$,

found 1333.3610.

Synthesis of {(2*S*,2'*S*,2''*S*)-[(1,3,5,2,4,6-trioxatriborinane-2,4,6-triyl)tris(benzo[*d*]-thiazole-6,2-diyl)]tris{2-[(S)-*sec*-butyl]-1,4-diazepane-4,1-diyl)}tris{[5-methyl-2-(2*H*-1,2,3-triazol-2-yl)phenyl]methanone} (4f). Starting from bromo derivative **2e** (291 mg, 526 µmol, 1 eq), the corresponding BPin ester was synthesized according to general procedure A, dissolved in acetone (1.5 mL) and methylboronic acid (315 mg, 5.26 mmol, 10 eq) and aqueous NaOH solution (0.1 M, 1.5 mL) were added under continuous stirring. The reaction was stirred at room temperature overnight and neutralized to pH 7 by the addition of aqueous HCl (0.1 M). After coevaporation by addition of H_2O (5 mL, 3×) at 40 °C under reduced pressure, the intermediate boronic acid was purified by flash column chromatography [RP-C18, 30-40% CH_3CN + 0.1% TFA] and obtained as red brown solid after lyophilization (34.4 mg, 66.4 µmol, 13%; TLC (DCM:methanol (80:1 (v/v), containing 1% (v/v) of 25% (w/w) ammonia solution)) R_f = 0.24; ESI-MS *m/z* 519.4 $[M+H]^+$; HRMS *m/z* $[M+H]^+$ calcd. 519.2344 for $C_{26}H_{31}BN_6O_3S$, found 519.2347). According to general procedure B, the boronic acid intermediate (34.4 mg, 66.4 µmol) was quantitatively converted into the boroxine. Boroxine **4f** was obtained as beige solid (33.2 mg, 22.1 µmol, 13% referred to **2e**) and stored in a desiccator over phosphorus pentoxide. MALDI-TOF *m/z* $[M+H]^+$ calcd. 1501.6570 for $C_{78}H_{88}B_3N_{18}O_6S_3$, found 1501.7652.

Synthesis of *tert*-butyl (*S*)-2-[(*S*)-*sec*-butyl]-1,4-diazepane-1-carboxylate (6) (solution in DMF). Starting from commercially available L-isoleucine, compound **6** was synthesized as described previously [2]. In the final synthesis step, the lactam (*tert*-butyl (*S*)-2-[(*S*)-sec-butyl]-3-oxo-1,4-diazepane-1-carboxylate (1.61 g, 5.94 mmol) was dissolved in dry THF (30 mL) under argon atmosphere and a solution of BH_3-THF complex (1 M, 30 mL, 5 eq) was added dropwise under continuous stirring. The solution was then heated overnight at 75 °C under reflux and then cooled on an ice bath for slow dilution with aqueous HCl (0.1 M, 47 mL). The

pH was adjusted to 10 with aqueous NaOH (0.1 M) and NaCl (2 g) was added. The organic and aqueous layer were separated and the aqueous layer was repeatedly extracted with methyl-*tert*-butyl ether (MTBE, 40 mL, 5×). The solvent was carefully removed under reduced pressure (max. 420 mbar at 40 °C) until compound **6** started to co-evaporate, which was monitored by TLC analysis of the distillate (silica TLC, n-hexane:EtOAc (3:1), R_f-(**6**) = 0.35, ninhydrin staining). MTBE was repeatedly added and evaporated to minimize the remaining solvent. The solution of crude **6** was diluted with DMF to a total volume of 10.8 mL, to reach a final concentration of 182 mM (referred to the lactam educt) and used for further reactions without further purification, since severe losses of **6** were observed after flash column chromatography (silica, iso-hexane:EtOAc (5:1 to 3:1), obtaining **6** as white yellowish solid (51.6 mg, 201 µmol, 4%)). ESI-MS: m/z 513.4 $[M+H]^+$; all analytical data were as previously reported [2].

Synthesis of *tert*-butyl 4-(5-fluorobenzoxazol-2-yl)-1,4-diazepane-1-carboxylate (7a). 5-Fluorobenzoxazole **5** (88.5 mg, 0.65 mmol) and *tert*-butyl 1,4-diazepane-1-carboxylate (0.14 g, 0.70 mmol) and copper(II) acetate hydrate (0.13 g, 0.66 mmol) were dissolved in CH_3CN (5 mL). Concentrated acetic acid (73.9 µL, 1.3 mmol) was added and the reaction mixture was stirred under air at 80 °C for 26 h. The reaction mixture was cooled to ambient temperature, diluted with ethyl acetate (30 mL) and washed with saturated sodium bicarbonate solution (3×20 mL). The organic layer was dried over sodium sulfate, filtered and concentrated under reduced pressure. The crude product was purified by flash column chromatography applying a gradient of 30% to 50% ethyl acetate in isohexane. Compound **7a** was obtained as brown oil (70.2 mg, 0.21 mmol, 32%). ESI-MS: *m/z* 336.2 $[M+H]^+$; ^1H NMR (400 MHz, $CDCl_3$) δ 7.15 (dd, J = 8.7, 4.3 Hz, 1H), 7.05 (dd, J = 8.9, 2.5 Hz, 1H), 6.71 (ddd, J = 9.6, 8.7, 2.6 Hz, 1H), 3.85 – 3.77 (m, 2H), 3.73 (t, J = 6.1 Hz, 2H), 3.63 (t, J = 5.3 Hz, 2H), 3.43 (dt, J = 29.1, 6.0 Hz, 2H), 2.07 – 1.98 (m, 2H), 1.43, 1.42 (s, 9H). ^{13}C NMR (101 MHz, $CDCl_3$, two sets of signals were observed, rotamers) δ 160.1 (d, J = 238.4 Hz), 154.9 (d, J = 32.6 Hz), 144.9, 108.6,

107.1 (d, *J* = 25.8 Hz), 103.2 (d, *J* = 26.7 Hz), 80.0 (d, *J* = 7.6 Hz), 49.6 (d, *J* = 22.6 Hz), 47.8, 47.6 (d, *J* = 10.3 Hz), 46.2 (d, *J* = 44.6 Hz), 28.3, 26.5 (d, *J* = 28.5 Hz).

Synthesis of *tert*-butyl (*S*)-2-[(*S*)-*sec*-butyl]-4-(5-fluorobenzoxazol-2-yl)-1,4-diazepane-1-carboxylate (7b). 5-Fluorobenzoxazole **5** (19.5 mg, 0.14 mmol) and *tert*-butyl (*S*)-2-[(*S*)-*sec*-butyl]-1,4-diazepane-1-carboxylate (**6**) (38.1 mg, 0.15 mmol) and copper(II) acetate hydrate (29.4 mg, 0.14 mmol) were dissolved in CH_3CN (10 mL). Concentrated acetic acid (16.3 µL, 0.28 mmol) was added and the reaction mixture was stirred under air at 80 °C for 46 h. The reaction mixture was cooled to ambient temperature, diluted with ethyl acetate (30 mL) and washed with saturated aqueous sodium bicarbonate solution (3×20 mL). The organic layer was dried over sodium sulfate, filtered and concentrated under reduced pressure. The crude product was purified by flash column chromatography applying a gradient of 20% to 50% ethyl acetate in isohexane. Compound **7b** was obtained as brown oil (20.3 mg, 51.9 µmol, 36%). ESI-MS: *m/z* 392.2 $[M+H]^+$.

Synthesis of 2-(1,4-diazepan-1-yl)-5-fluorobenzoxazole (8a). *Tert*-butyl 4-(5-fluorobenzoxazol-2-yl)-1,4-diazepane-1-carboxylate (**7a**) (59.4 mg, 0.18 mmol) was dissolved in dichloromethane (3 mL). Trifluoroacetic acid (0.3 mL) was added and the reaction mixture was stirred at ambient temperature for 5 h. The reaction mixture was basified to pH 11 using 1 M aqueous NaOH solution. The product was extracted with dichloromethane (3×). The combined organic layers were dried over sodium sulfate, filtered and concentrated under reduced pressure. Compound **8a** was obtained as yellow oil (41.5 mg, 0.18 mmol, quant.) and used for the next step without purification. ESI-MS: *m/z* 236.0 $[M+H]^+$; ^1H NMR (400 MHz, $CDCl_3$) δ 7.13 (dd, *J* = 8.7, 4.4 Hz, 1H), 7.03 (dd, *J* = 9.0, 2.6 Hz, 1H), 6.69 (ddd, *J* = 9.6, 8.7, 2.6 Hz, 1H), 3.87 – 3.72 (m, 4H), 3.17 – 3.04 (m, 2H), 3.01 – 2.90 (m, 2H), 2.00 (d, *J* = 5.0 Hz, 2H). ^{13}C NMR (101 MHz, $CDCl_3$) δ 163.3, 160.1 (d, *J* = 237.9 Hz), 145.1 (d, *J* = 1.3 Hz), 144.5 (d, *J* = 13.5 Hz), 108.5 (d, *J* = 10.4 Hz), 106.7 (d, *J* = 25.9 Hz), 103.2 (d, *J* = 26.6 Hz), 49.6,

49.6, 49.2, 48.6, 48.5, 47.9, 47.3, 47.0, 29.4 (d, J = 67.0 Hz).

Synthesis of 2-{(*S*)-3-[(*S*)-*sec*-butyl]-1,4-diazepan-1-yl}-5-fluorobenzoxazole (8b). *Tert*-butyl (*S*)-2-[(*S*)-*sec*-butyl]-4-(5-fluorobenzoxazol-2-yl)-1,4-diazepane-1-carboxylate (**7b**) (17.9 mg, 45.7 µmol) was dissolved in dichloromethane (2 mL). Trifluoroacetic acid (0.2 mL) was added and the reaction mixture was stirred at ambient temperature for 4 h. The reaction mixture was basified to pH 11 using 1 M aqueous NaOH solution. The product was extracted with dichloromethane (3×). The combined organic layers were dried over sodium sulfate, filtered and concentrated under reduced pressure. Compound **8b** was obtained as yellow oil (13.3 mg, 45.7 µmol, quant.) and used for subsequent reactions without purification. ESI-MS: *m/z* 292.1 $[M+H]^+$.

Synthesis of 2-{(*S*)-3-[(*S*)-*sec*-butyl]-1,4-diazepan-1-yl}-6-fluorobenzo[*d*]thiazole (9a). According to general procedure C, **6** (51.6 mg, 201 µmol, 1 eq) reacted with 2-chloro-6-fluoro-benzo[*d*]thiazole. The intermediate *tert*-butyl (*S*)-2-[(*S*)-*sec*-butyl]-4-(6-fluorobenzo[*d*]thiazol-2-yl)-1,4-diazepane-1-carboxylate was purified by flash column chromatography [silica, DCM:methanol (50:1 (v/v), containing 1% (v/v) of 25% (w/w) ammonia solution)] and directly converted into **9a** following the general procedure D. Compound **9a** was obtained after purification by flash column chromatography [silica, DCM:methanol (80:1 to 50:1 (v/v), containing 1% (v/v) of 25% (w/w) ammonia solution)] as brown, yellow oil (30.1 mg, 97.9 µmol, 49%). Silica TLC (DCM:methanol (80:1 (v/v), containing 1% (v/v) of 25% (w/w) ammonia solution)) R_f (Boc-intermediate) = 0.76, R_f (**9a**) = 0.24; ESI-MS (Boc-intermediate): *m/z* 408.3 $[M+H]^+$, **9a**: *m/z* 308.1 $[M+H]^+$; ^1H NMR: (600 MHz, $CDCl_3$) δ 7.44 (dd, J = 8.8, 4.7 Hz, 1H), 7.30 (dd, J = 8.1, 2.6 Hz, 1H), 7.00 (td, J = 9.0, 2.6 Hz, 1H), 4.12 – 3.99 (m, 1H), 3.90 – 3.80 (m, 1H), 3.74 – 3.58 (m, 2H), 3.44 – 3.36 (m, 2H), 3.05 – 2.98 (m, 1H), 2.79 – 2.73 (m, 1H), 2.18 (s, 1H), 1.79 – 1.73 (m, 1H), 1.68 – 1.58 (m, 1H), 1.43 – 1.32 (m, 1H), 1.27 – 1.24 (m, 1H), 1.07 (d, J = 6.9 Hz, 3H), 1.00 (t, J = 7.4 Hz, 3H).

Synthesis of 2-{(*S*)-3-[(*S*)-*sec*-butyl]-1,4-diazepan-1-yl}-6-bromobenzo[*d*]thiazole (9b). According to general procedure C, a solution of **6** in MTBE (182 mM, 19.2 mL, 3.50 mmol, 1 eq) was reacted with 6-bromo-2-chloro-benzo[*d*]thiazole. The crude intermediate *tert*-butyl (*S*)-4-(6-bromobenzo[*d*]thiazol-2-yl)-2-[(*S*)-*sec*-butyl]-1,4-diazepane-1-carboxylate was purified by flash column chromatography [silica, DCM:methanol (80:1 (v/v), containing 1% (v/v) of 25% (w/w) ammonia solution)] and subsequently converted into **9b** following the general procedure D. Compound **9b** was obtained after purification by flash column chromatography [silica, DCM:methanol (40:1 to 10:1 (v/v), containing 1% (v/v) of 25% (w/w) ammonia solution)], as yellow oil (402 mg, 1.09 mmol, 30%). Silica TLC (DCM:methanol (20:1 (v/v), containing 1% (v/v) of 25% (w/w) ammonia solution)) R_f (intermediate) = 0.77, (DCM:methanol (10:1 (v/v), containing 1% (v/v) of 25% (w/w) ammonia solution)) R_f (**9b**) = 0.51; ESI-MS intermediate: *m/z* 470.1 $[M+H]^+$, **9b**: *m/z* 370.1 $[M+H]^+$.

Synthesis of (1,4-diazepan-1-yl)[5-methyl-2-(2*H*-1,2,3-triazol-2-yl)phenyl]methanone (10). A solution of the commercially available *tert*-butyl 1,4-diazepane-1-carboxylate (2.00 g, 10.0 mmol, 1 eq) in DMF (28 mM) together with commercially available 5-methyl-2-(2*H*-1,2,3-triazol-2-yl)benzoic acid (1.2 eq) and HATU (1.2 eq) was prepared. Under continuous stirring, DIPEA (5 eq) was added dropwise. The reaction was stirred overnight and the solvent was removed under reduced pressure. Subsequently, the residue was dissolved in DCM (15 mL) and washed with saturated $NaHCO_3$ solution (10 mL) and brine (10 mL), respectively. Afterwards, the organic layer was dried over Na_2SO_4, filtered and the solvent was removed under reduced pressure to obtain intermediate *tert*-butyl-4-(5-methyl-2-(2*H*-1,2,3-triazol-2-yl)benzoyl)-1,4-diazepane-1-carboxylate, which was subsequently converted to **10** following the general procedure D. Compound **10** was obtained after purification by flash column chromatography [silica, DCM:methanol (20:1 (v/v), containing 1% (v/v) of 25% (w/w) ammonia solution)] as beige solid (2.31 g, 8.10 mmol, 81%). Silica TLC (DCM:methanol

(10:1 (v/v), containing 1% (v/v) of 25% (w/w) ammonia solution)) R_f (Boc-intermediate) = 0.86; R_f (**10**) = 0.53; ESI-MS (Boc-intermediate): *m/z* 386.2 $[M+H]^+$, **10**: *m/z* 286.0 $[M+H]^+$; ^1H NMR (600 MHz, $CDCl_3$, doublets observed due to rotamers) δ 7.85 (dd, *J* = 14.6, 8.3 Hz, 1H), 7.78 (d, *J* = 4.4 Hz, 2H), 7.34 – 7.28 (m, 1H), 7.19 (s, 1H), 3.89 – 3.65 (m, 2H), 3.34 – 3.18 (m, 2H), 3.10 – 2.82 (m, 3H), 2.79 (s, 1H), 2.74 (t, *J* = 5.6 Hz, 1H), 2.40 (d, *J* = 2.3 Hz, 3H), 1.92 – 1.85 (m, 1H), 1.67 – 1.51 (m, 1H).

Determination of the molar activity of [^{18}F]1c and [^{18}F]1f. To determine the apparent molar activity (AMA, MBq/nmol) of **[^{18}F]1c** and **[^{18}F]1f**, respectively, the corresponding ^{19}F-substituted reference compound **1c** or **1f** were injected into the analytical HPLC system (sample loop: 20µL) in different amounts (1.25, 2.50, 5.00, 10.0, 20.0 and 50.0 nmol). The respective peak (UV-channel, 214 nm) was integrated and a calibration curve was prepared, plotting amount (nmol) against integral area (mAU) (Figure S1). The finally formulated radioligand **[^{18}F]1c** or **[^{18}F]1f**, respectively, was injected to the same HPLC system and the amount of non-radioactive substance was determined as mean value (nmol) from three independent injections by integration of the UV peak. Considering the total volume of the finally formulated radiotracer solution, the total amount of non-radioactive substance was calculated and the AMA was calculated as total amount of radioactivity (MBq) obtained at the end of synthesis divided by the total amount of non-radioactive substance (nmol).

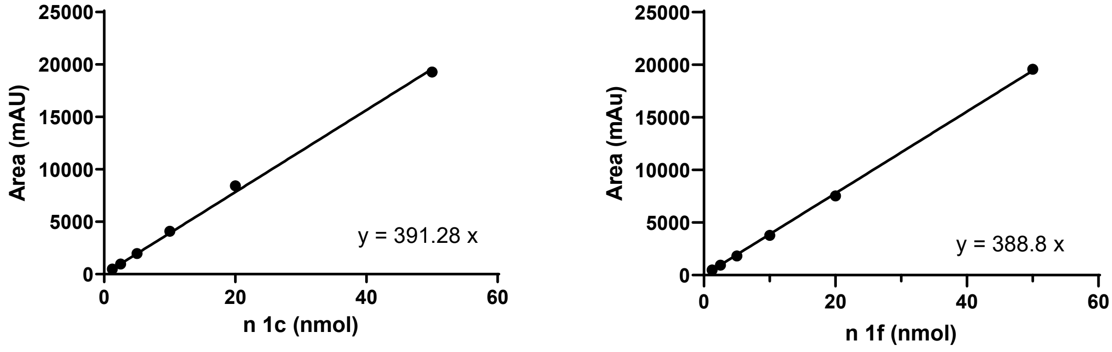

Figure S1. Calibration curves for the determination of the molar activity of **[^{18}F]1c** and **[^{18}F]1f**.

High temperature NMR experiments on 1f to study coalescence of the resonances

We would like to address the observation of four rotamers in the NMR-spectra of the compounds **1e**, **1f** and **2e**. We assumed that, besides of the tertiary amide bond (Figure S2, left arrow), also that one connecting the benzene ring with the amide group (Figure S2, right arrow) could lead to *cis*- and *trans*-conformers due to hindered rotation, presumably due to the introduction of the (*S*)-*sec*-butyl residue into the diazepane ring.

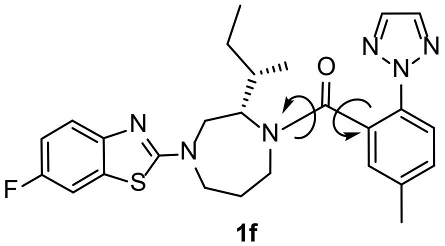

1f

Figure S2. Two rotatable single bonds presumably leading to *cis*- and *trans*-conformations of compound **1f**.

Therefore, we have recorded ^{13}C and ^{1}H-NMR spectra of compound **1f** in $C_2D_2Cl_4$ at 373 K and we were able to observe coalescence of the resonance signals for both nuclei.

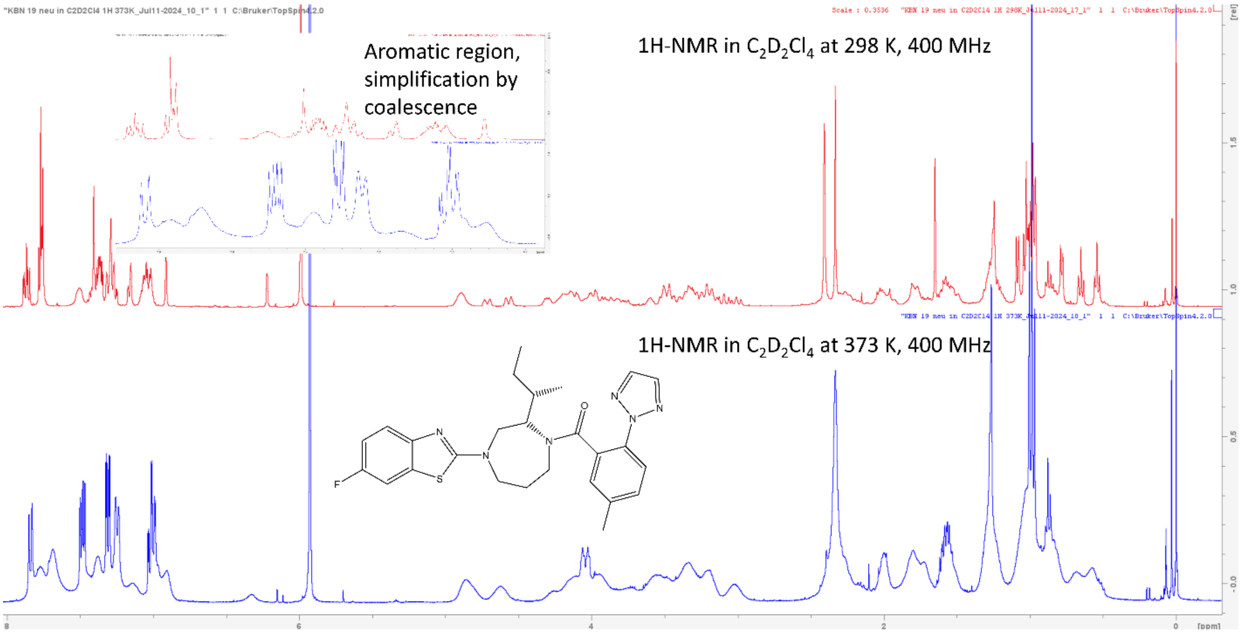

Figure S3. Comparison of the ^{1}H-NMR-spectra of compound **1f** at rt (298 K) and elevated temperature (373 K).

In Figure S3, a comparison of the ^{1}H-NMR-spectra at 298 K and 373 K is provided, in particular recognizing a significant simplification of the aromatic resonance area at 373 K, thus allowing to clearly observe systems of first order.

In Figure S4, a significant section of the DEPT-Q spectrum of compound **1f** in $CDCl_3$ at 298 K was compared to a normal ^{13}C-spectrum (which gave better results at 373 K due to simpler pulsing) in $C_2D_2Cl_4$ at 373 K. We were able to observe a clear simplification of four doublets (due to the $^{3}J(^{13}C-^{19}F)$-coupling) representing C-4 of the benzothiazole scaffold to a single doublet. Thus, we have clearly demonstrated the presence of four rotamers for **1f** at rt.

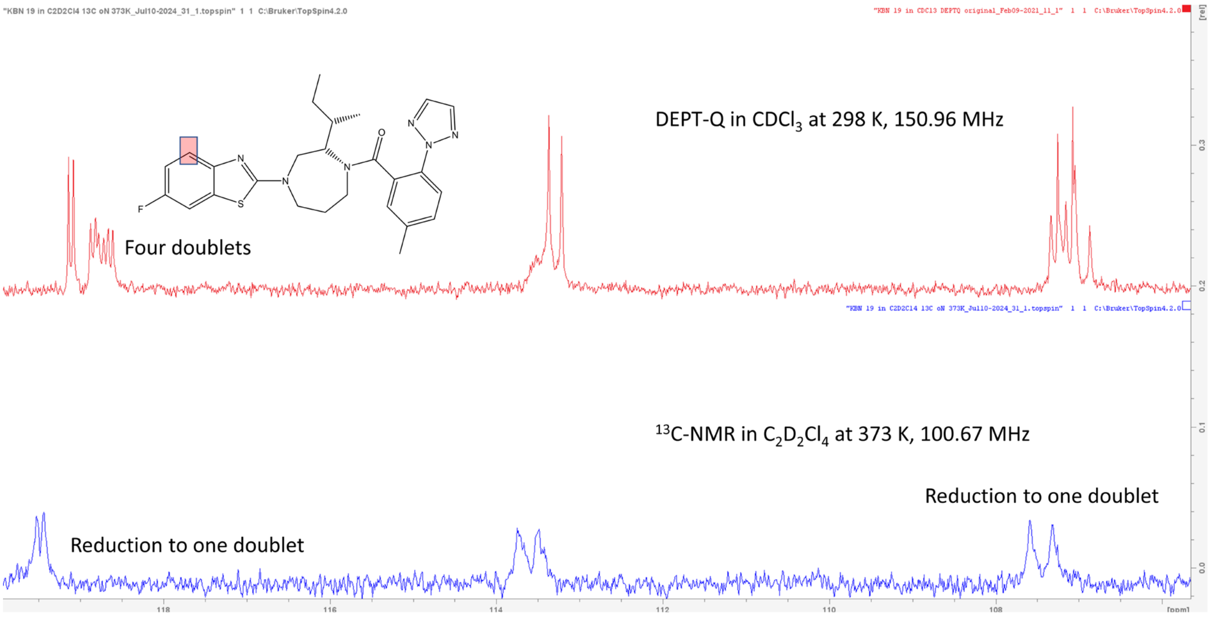

Figure S4. Comparison of a significant section of the ^{13}C-NMR-spectra of compound **1f** at rt (298 K) and elevated temperature (373 K).

NMR Spectra

^{1}H-NMR of **1a**

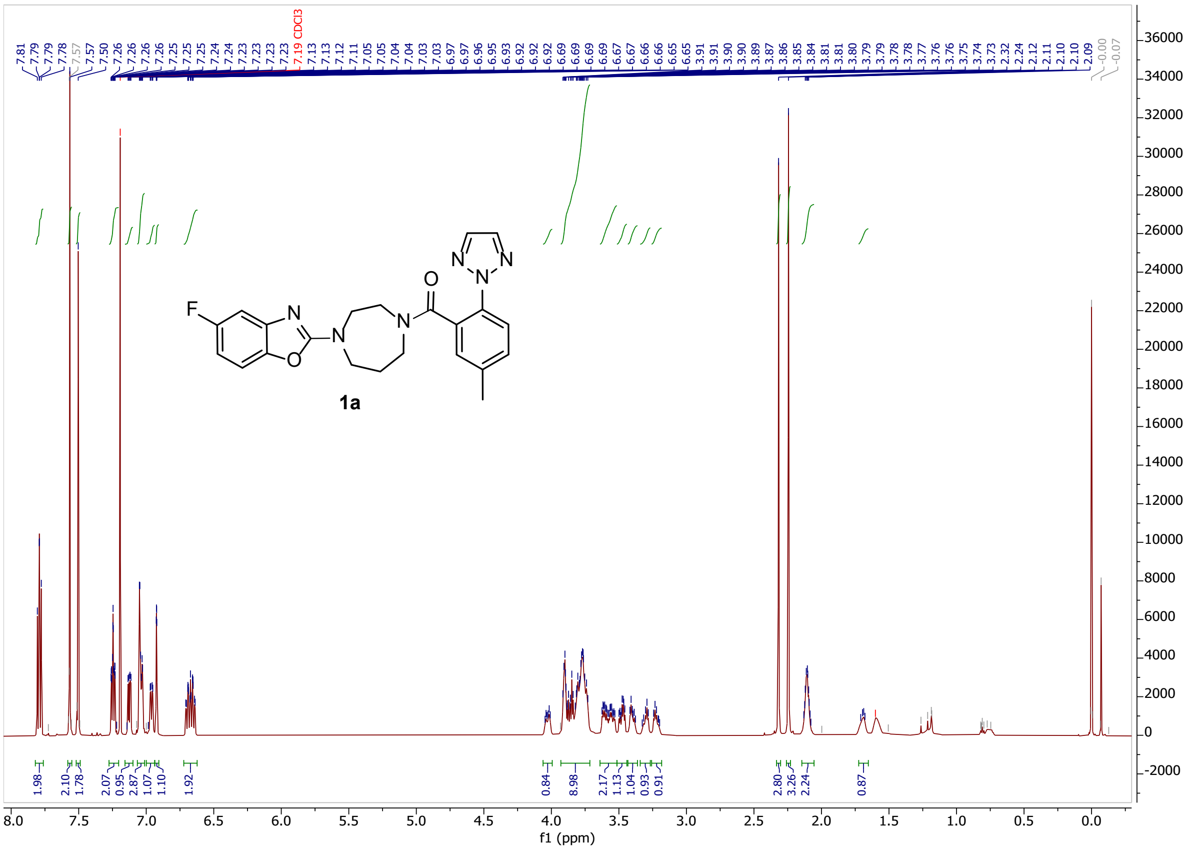

DEPTQ-NMR of **1a**

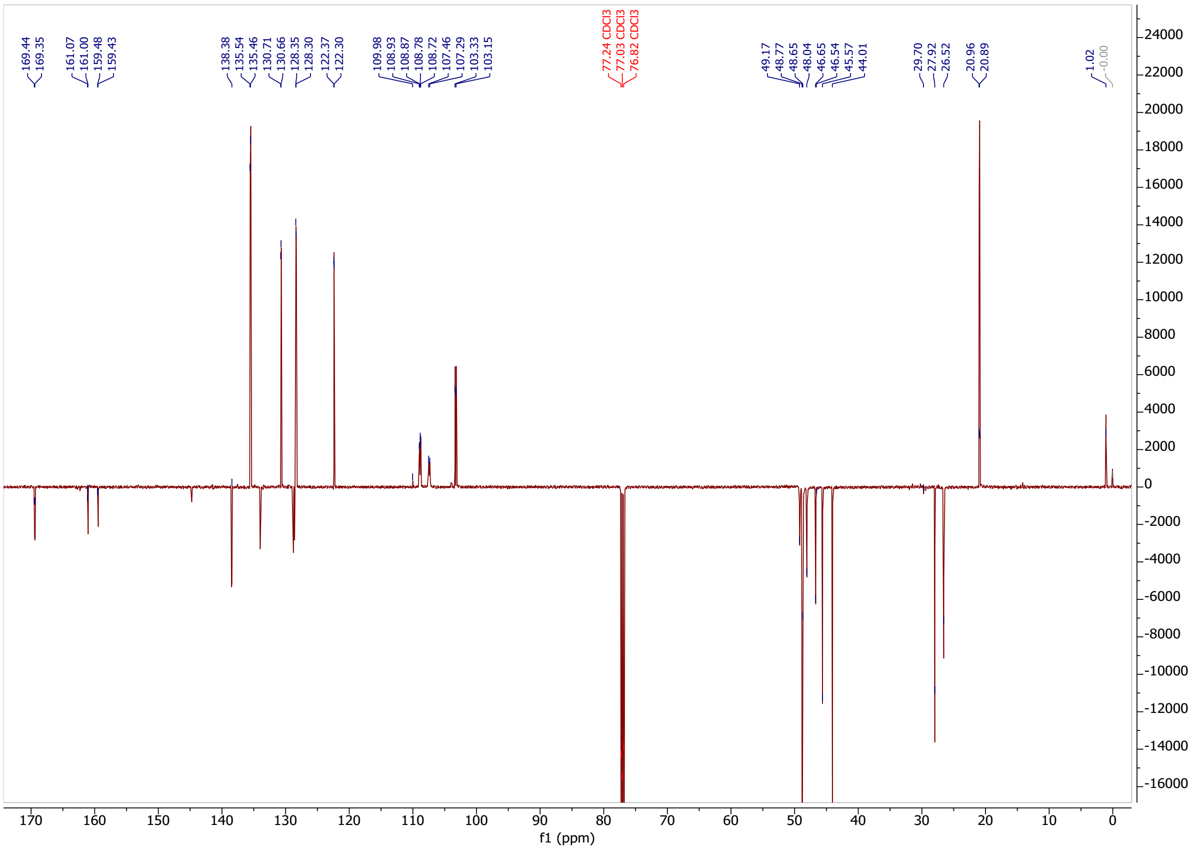

HSQC-NMR of **1a**

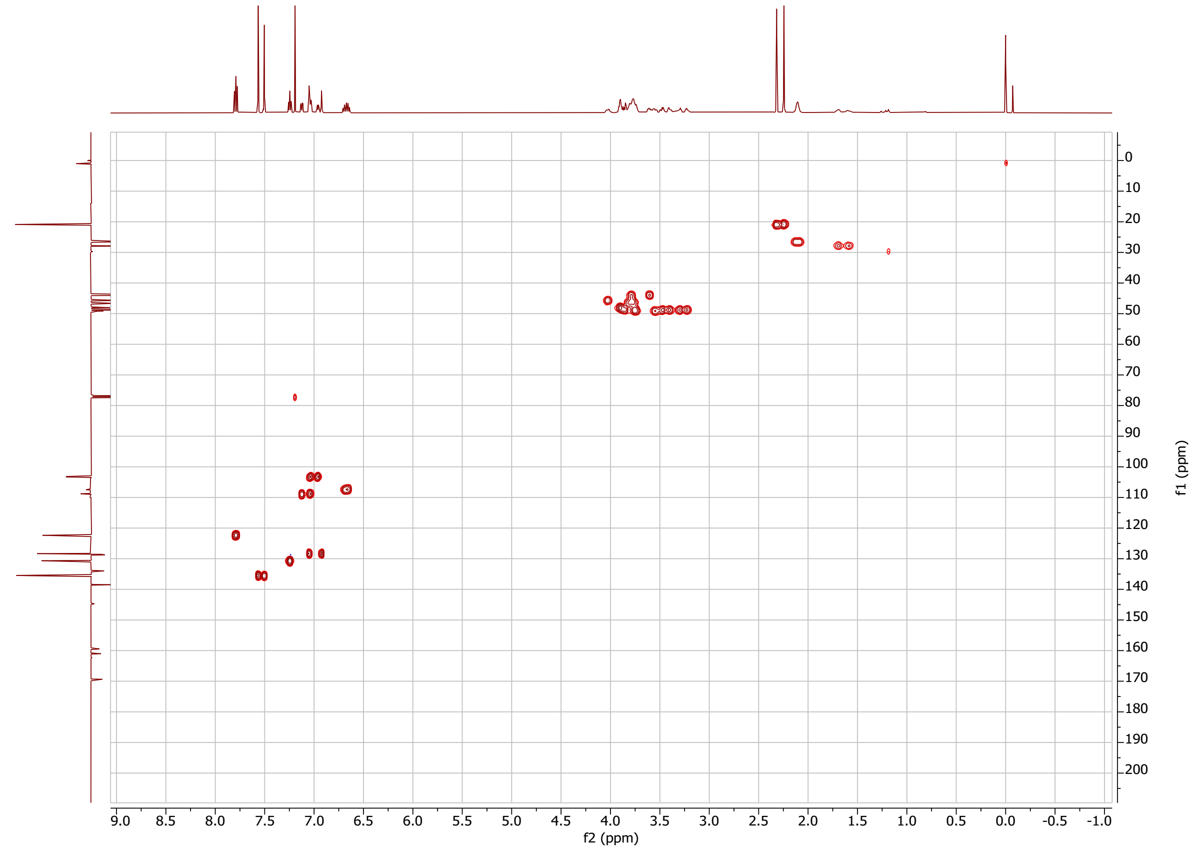

HMBC-NMR of **1a**

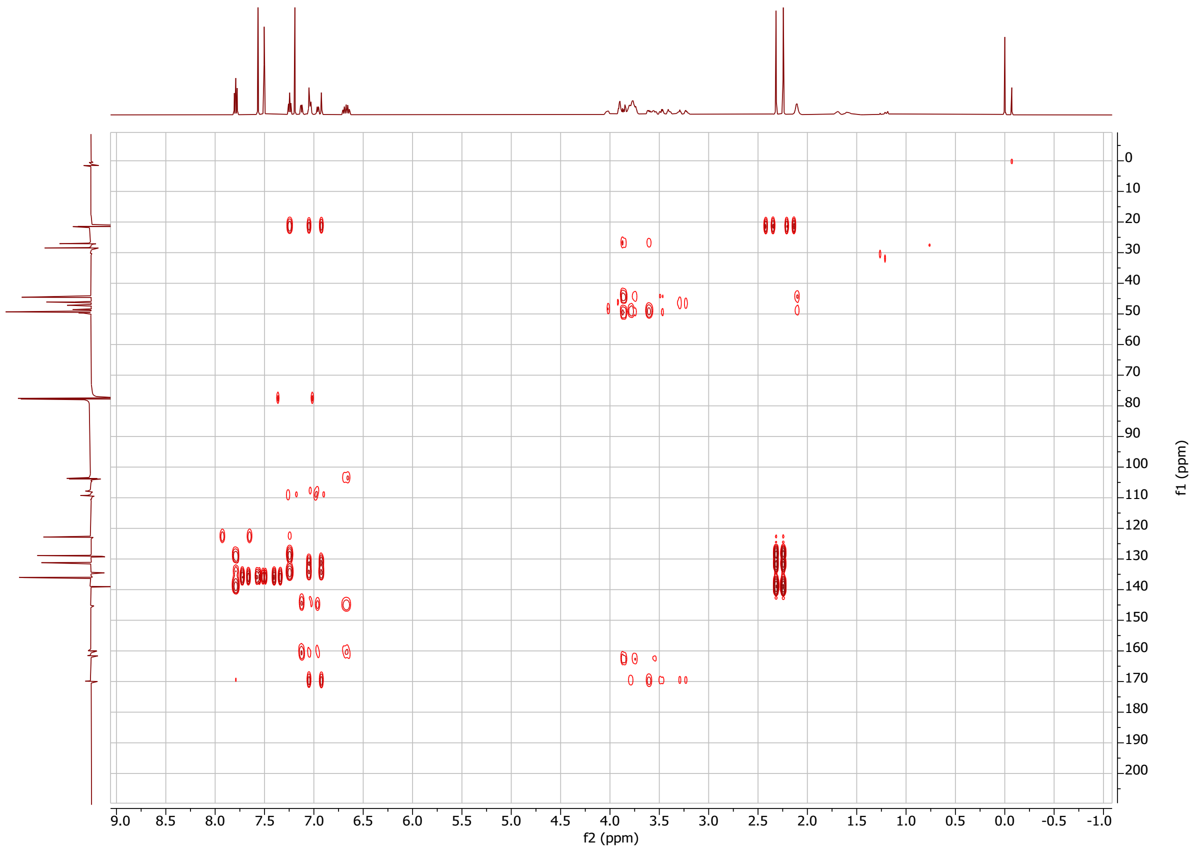

COSY-NMR of **1a**

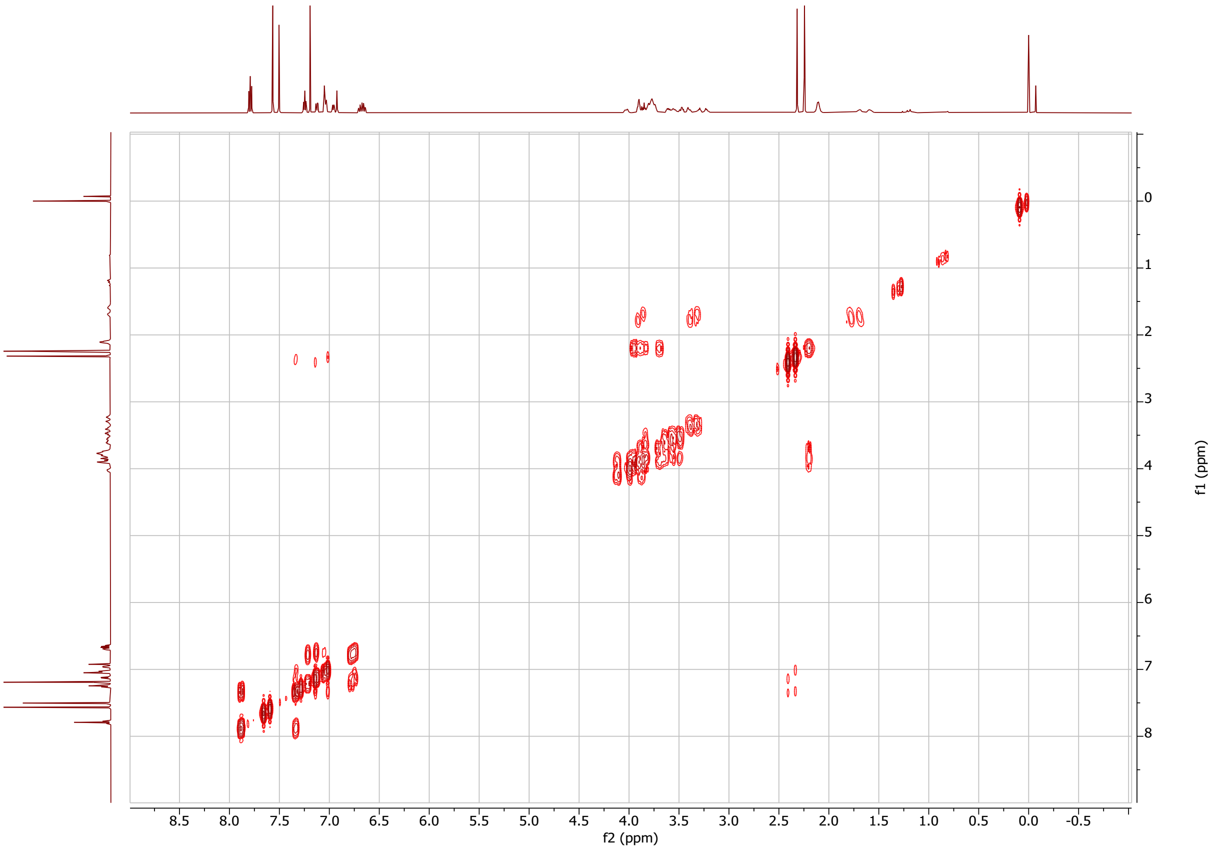

^{19}F-NMR of **1a**

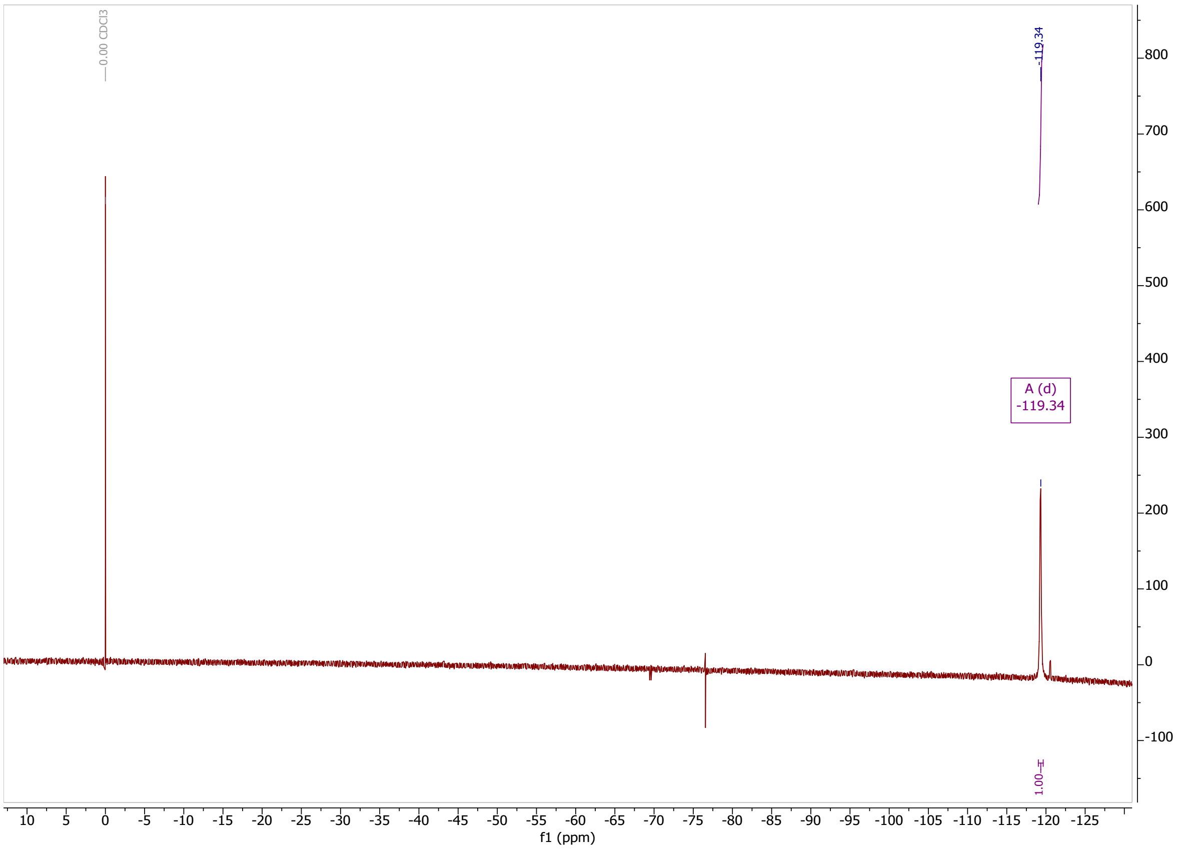

^{1}H-NMR of **1b**

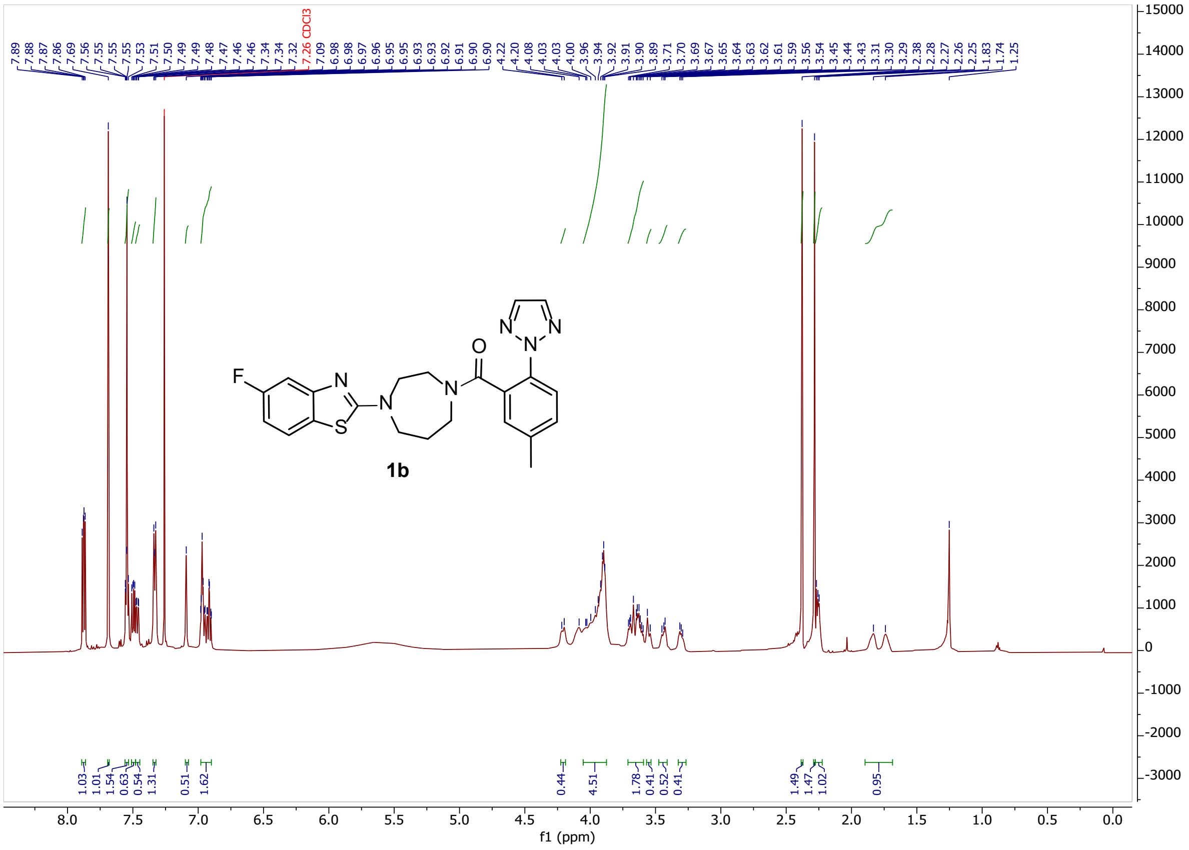

DEPTQ-NMR of **1b**

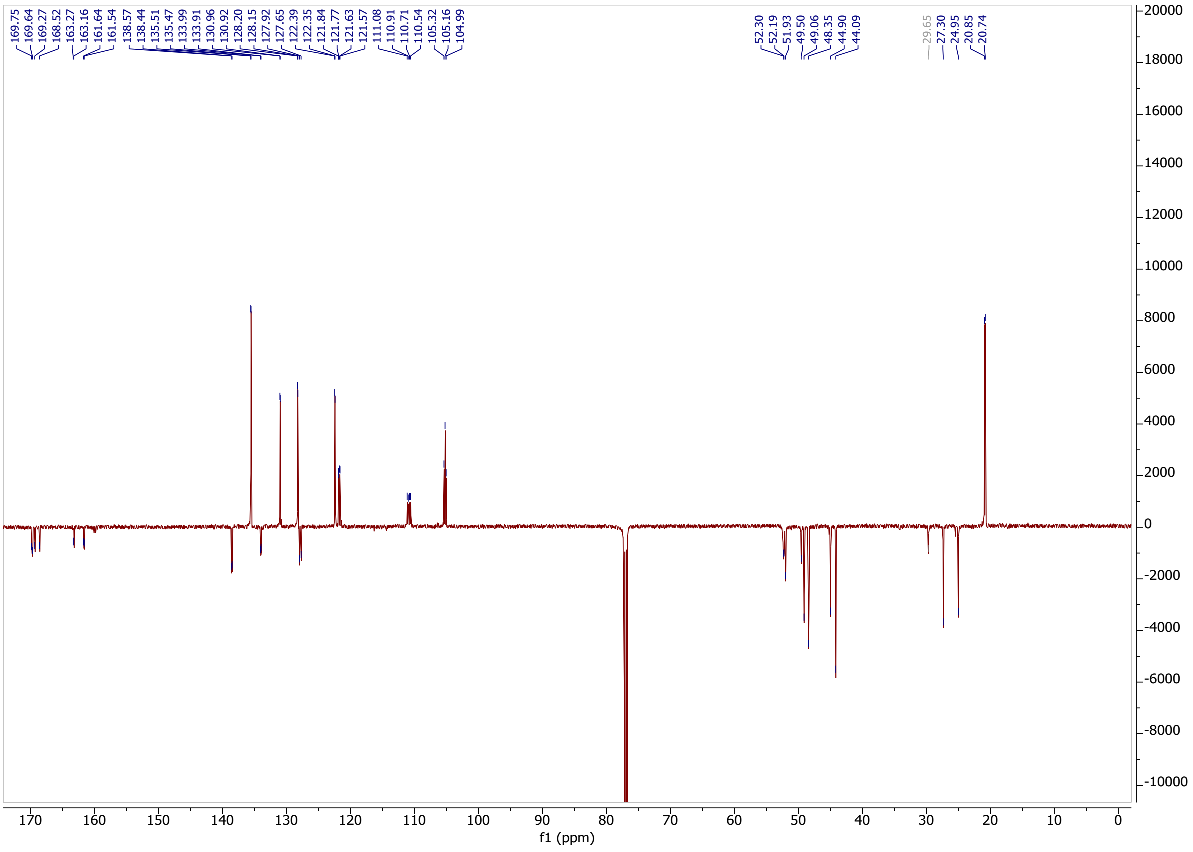

^{1}H-NMR of **1c**

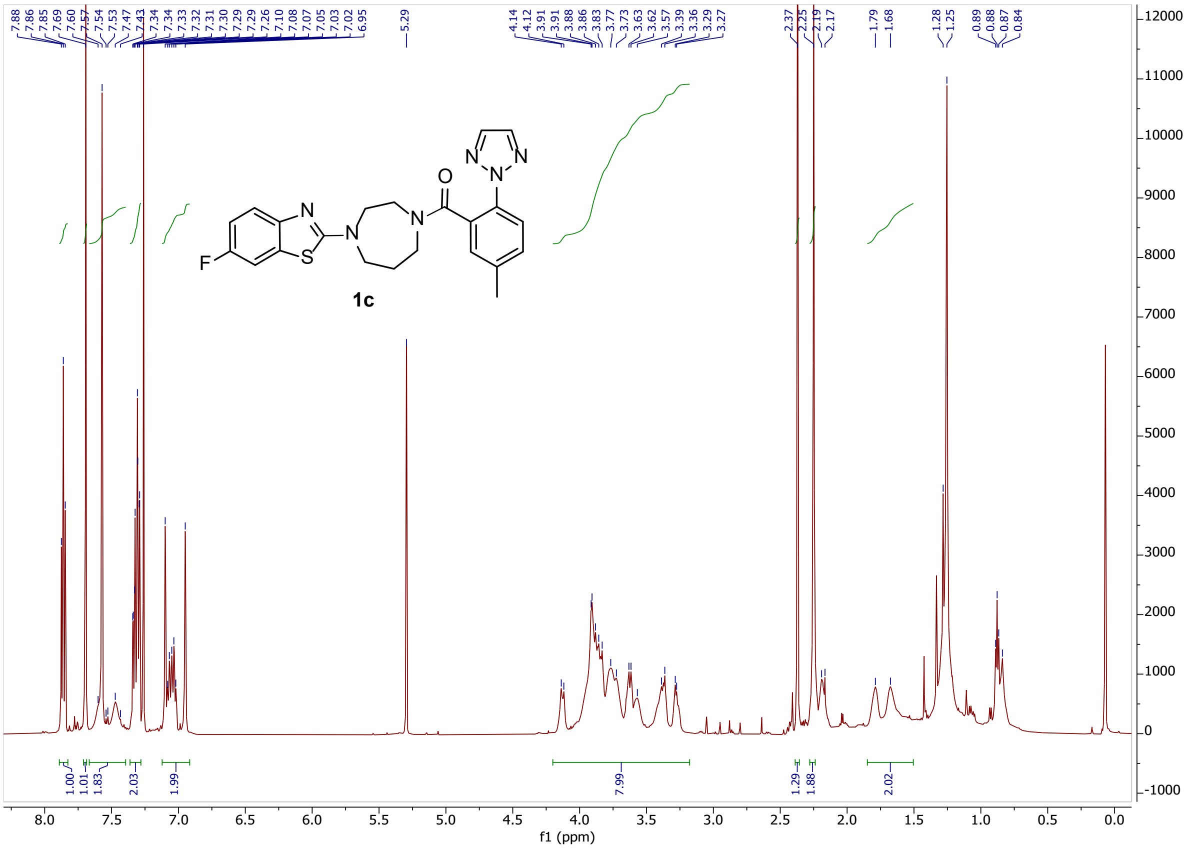

DEPTQ-NMR of **1c**

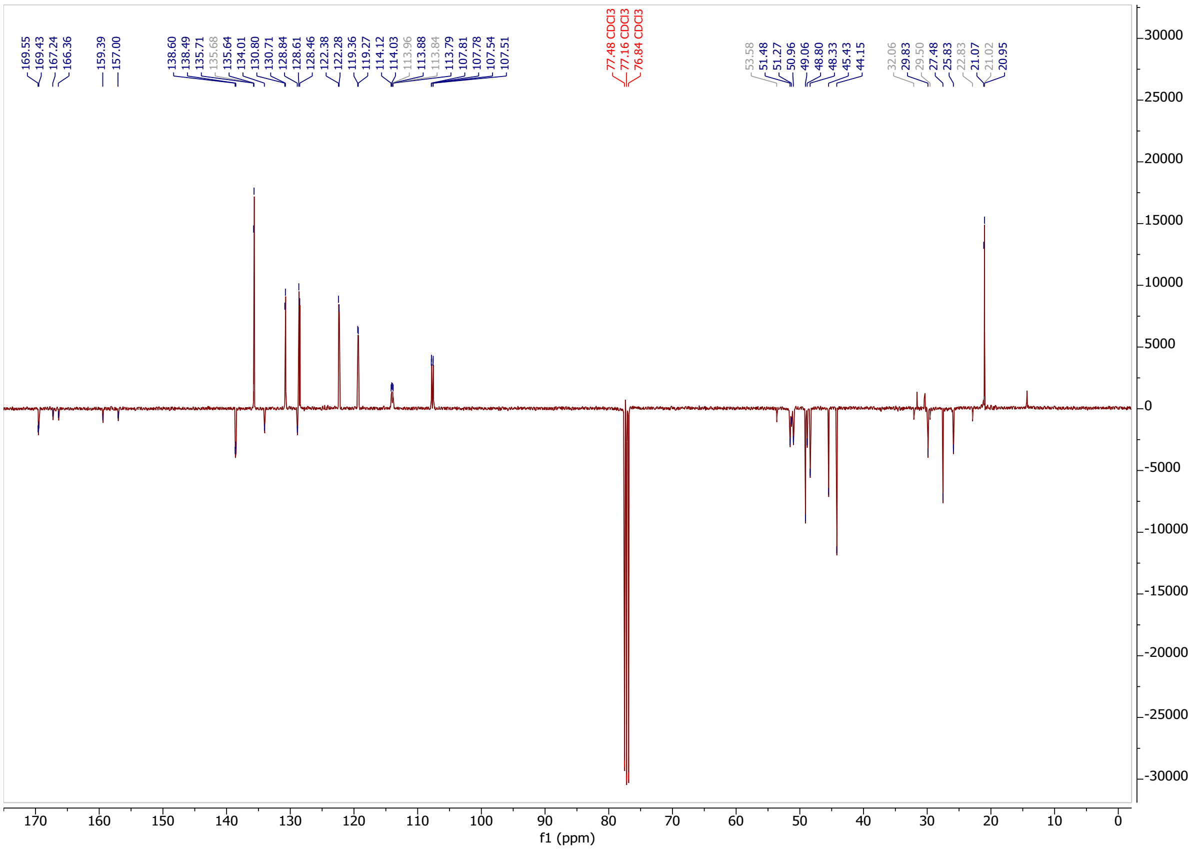

^{1}H-NMR of **1d**

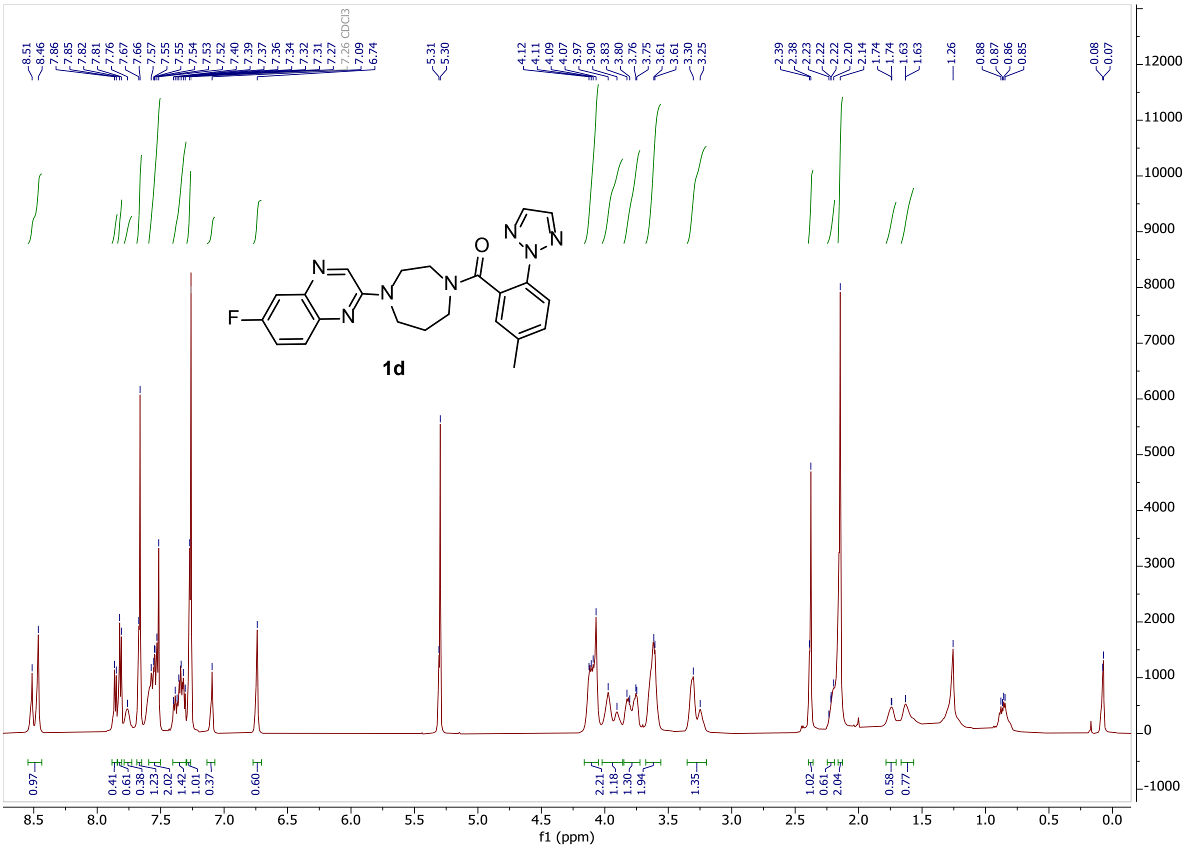

DEPTQ-NMR of **1d**

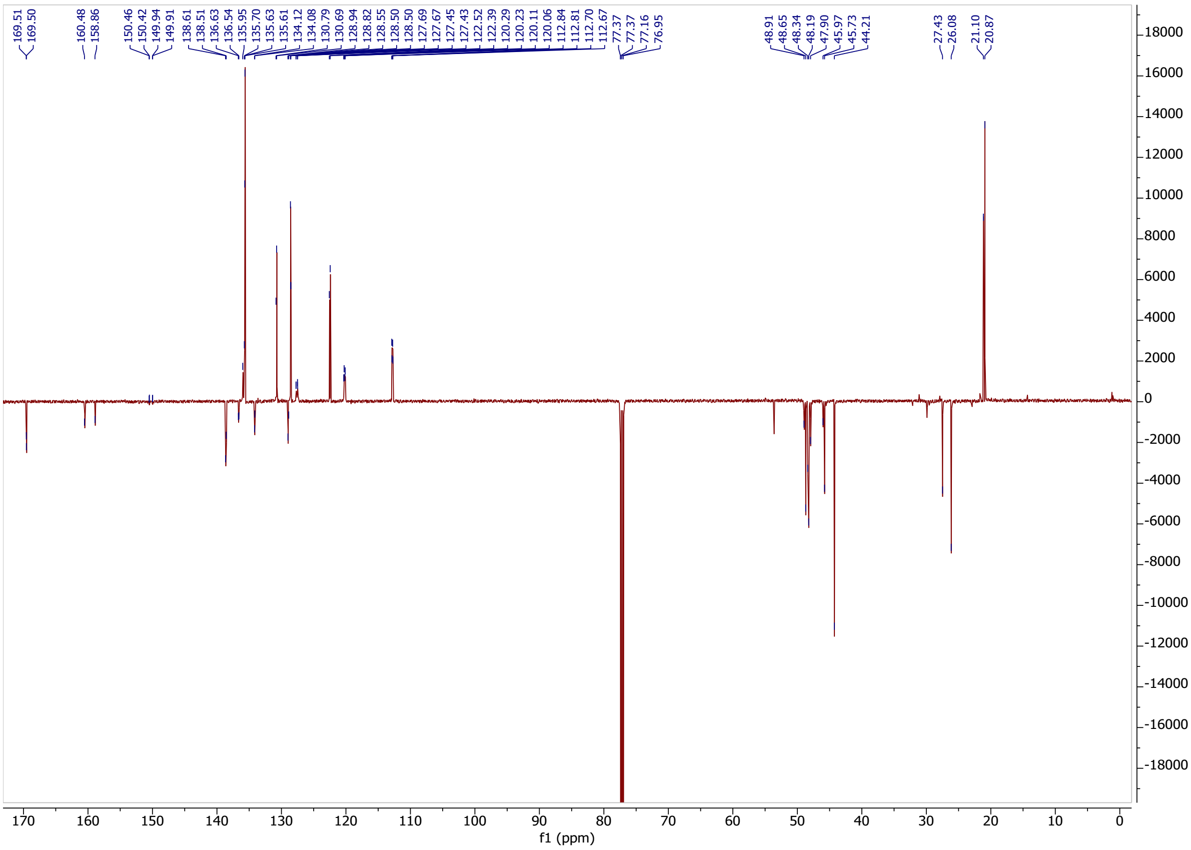

^{1}H-NMR of **1e**

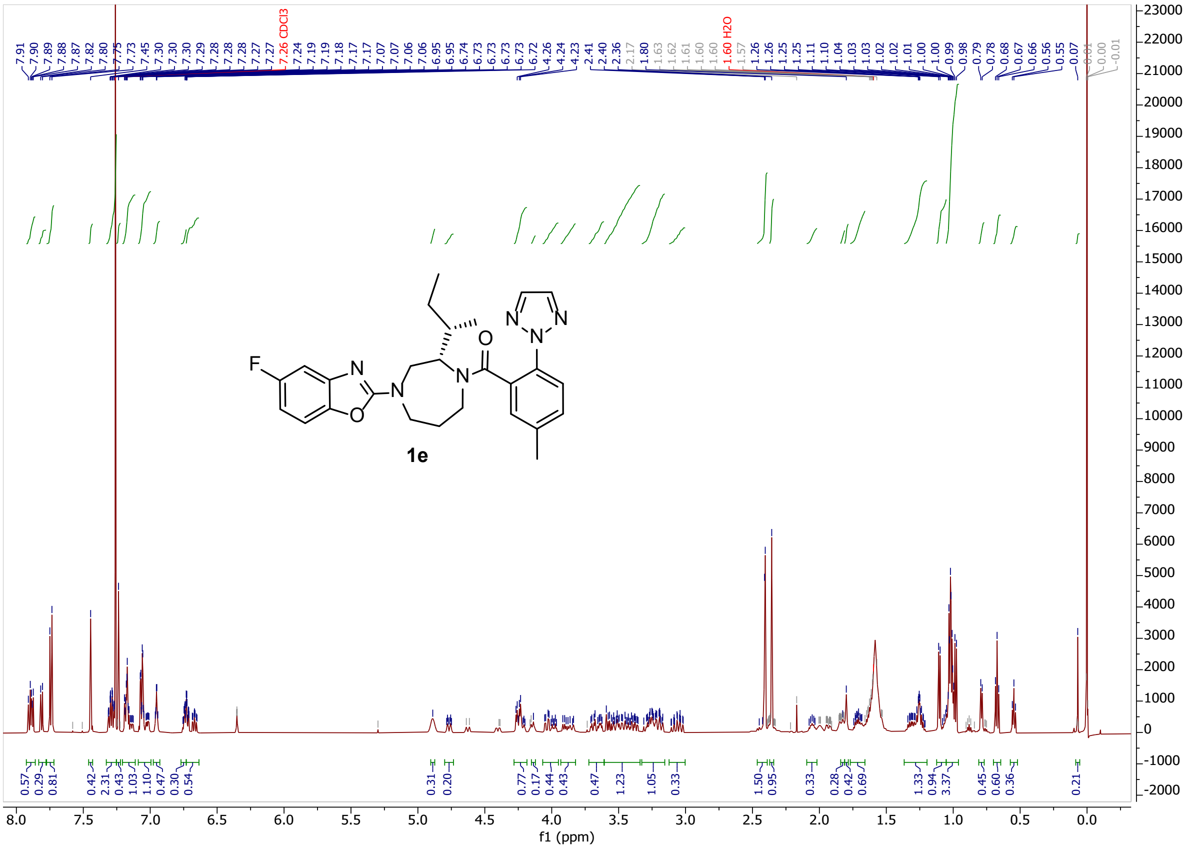

DEPTQ-NMR of **1e**

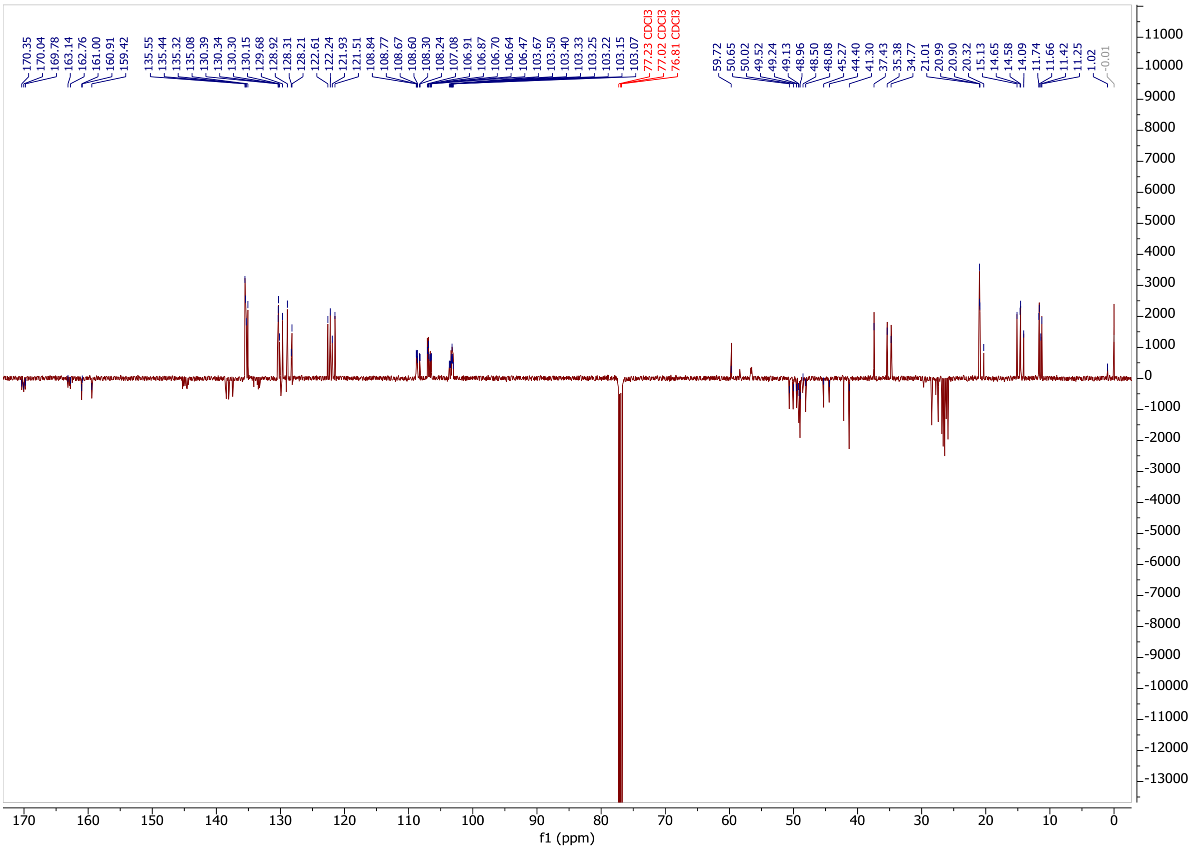

COSY-NMR of **1e**

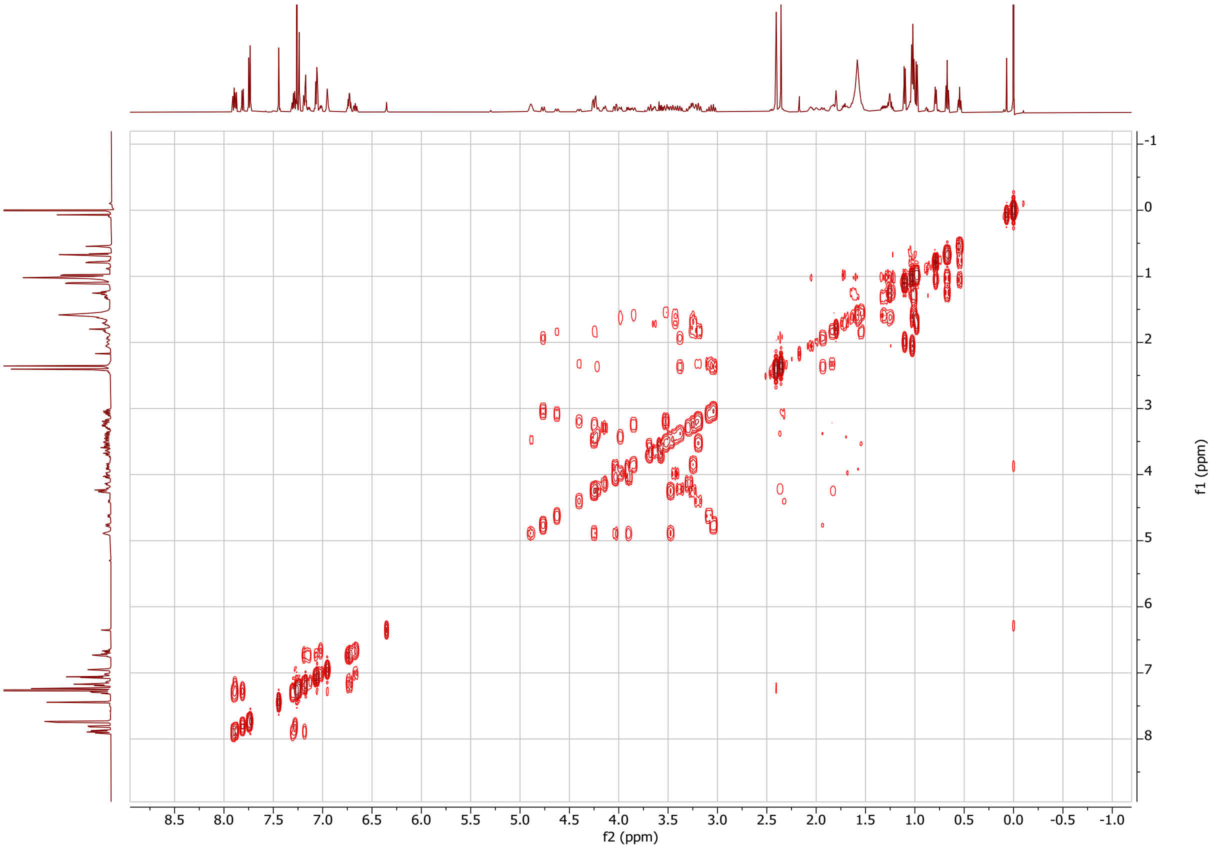

HSQC NMR of **1e**

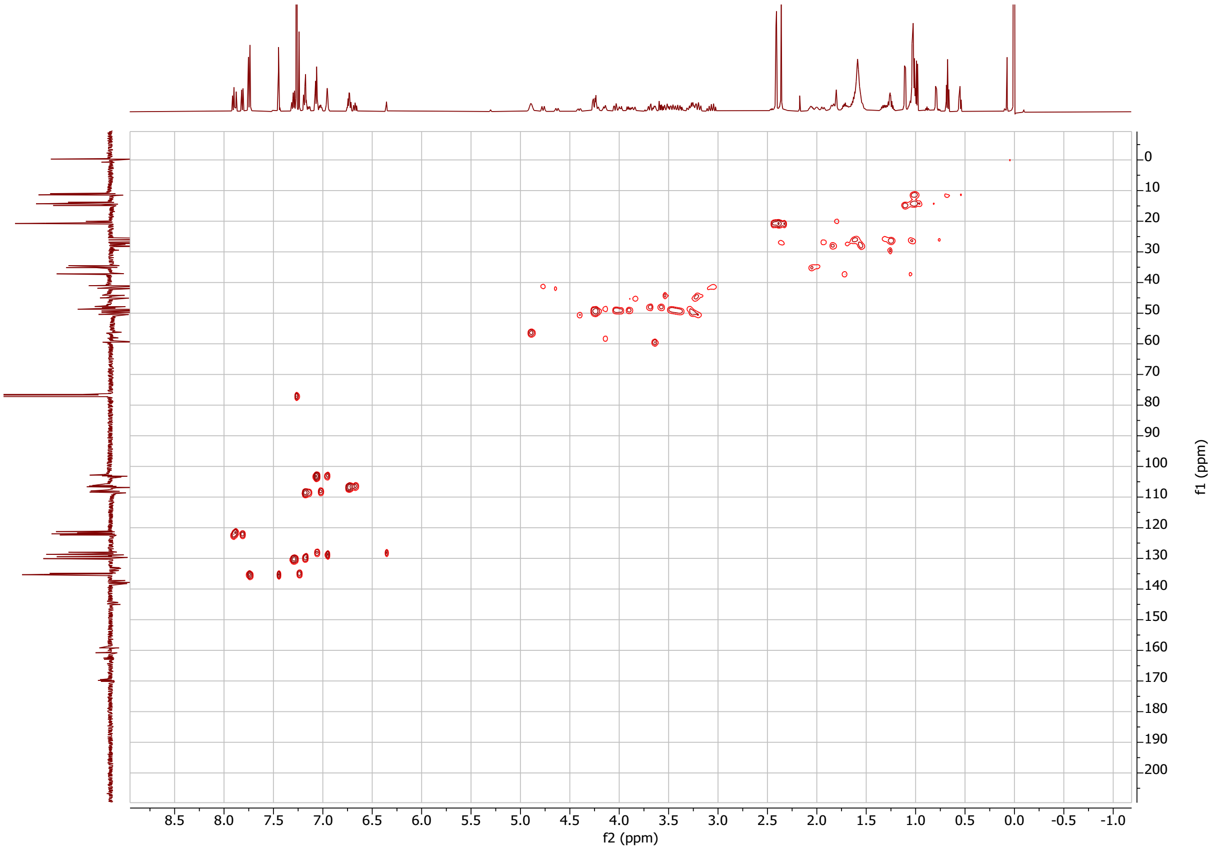

HMBC NMR of **1e**

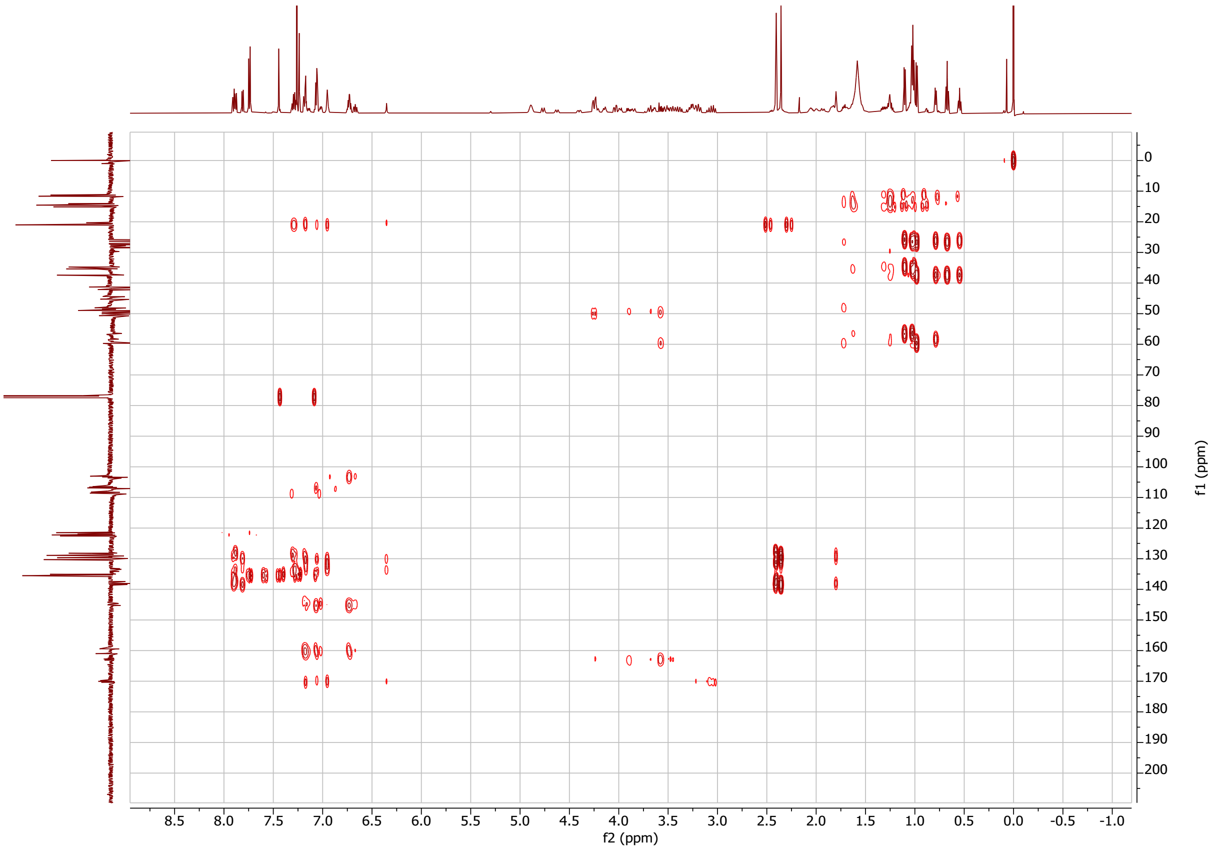

^{19}F-NMR of **1e**

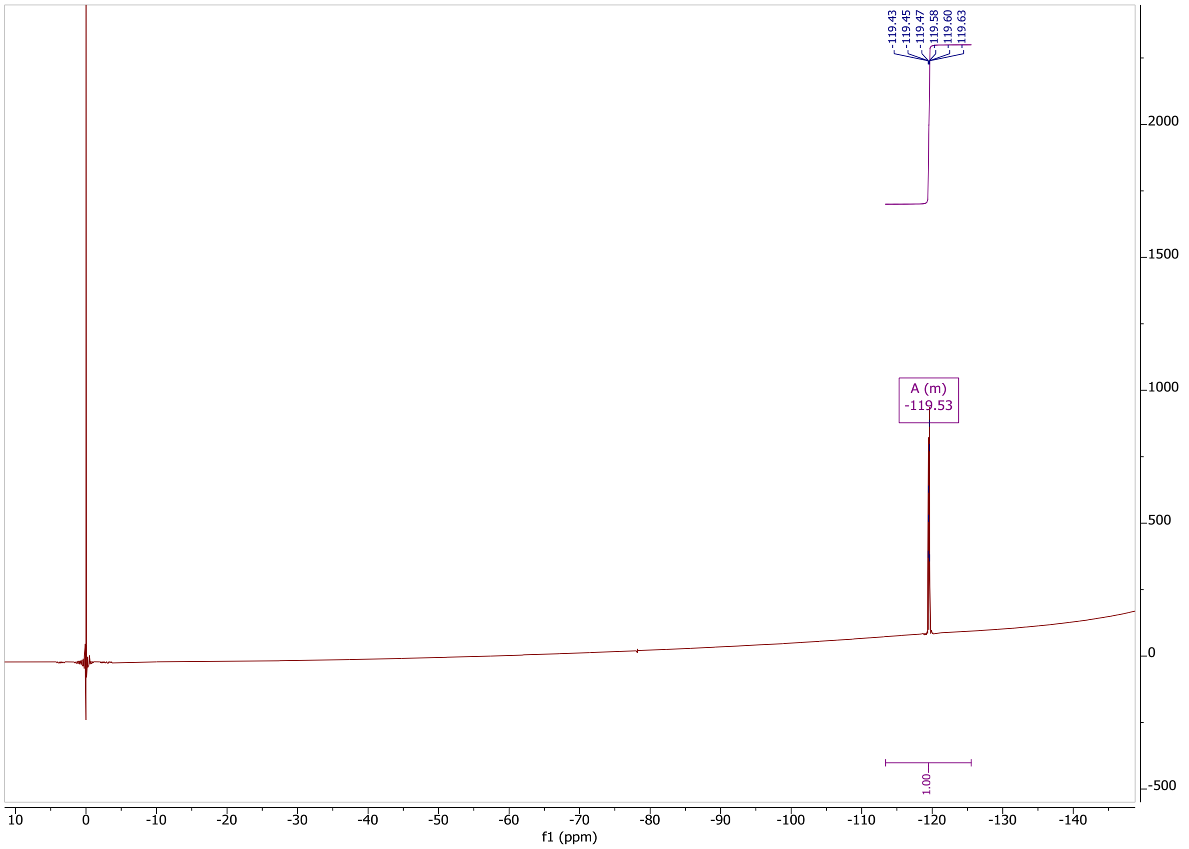

DEPTQ-NMR of **1e** (aliphatic region):

Integration of the resonances of the terminal CH_3-group of the *sec*-butyl substituent gave a summed integral of 0.9584. Percentages of each single peak area were: 27% of the first (left), 31% of the second, 17% of the third, and 25% of the fourth peak (four rotamers of **1e**).

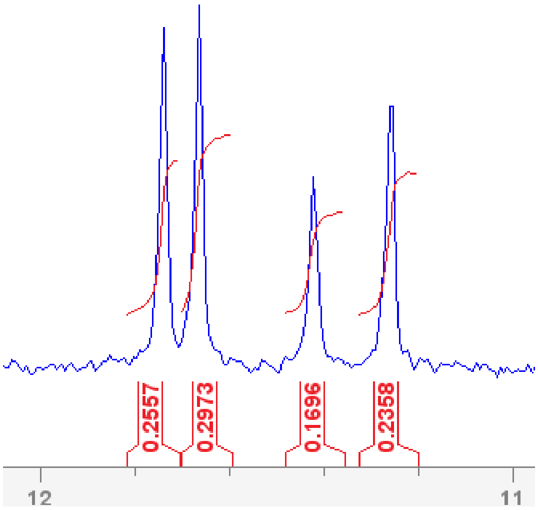

^{1}H-NMR of **1f**

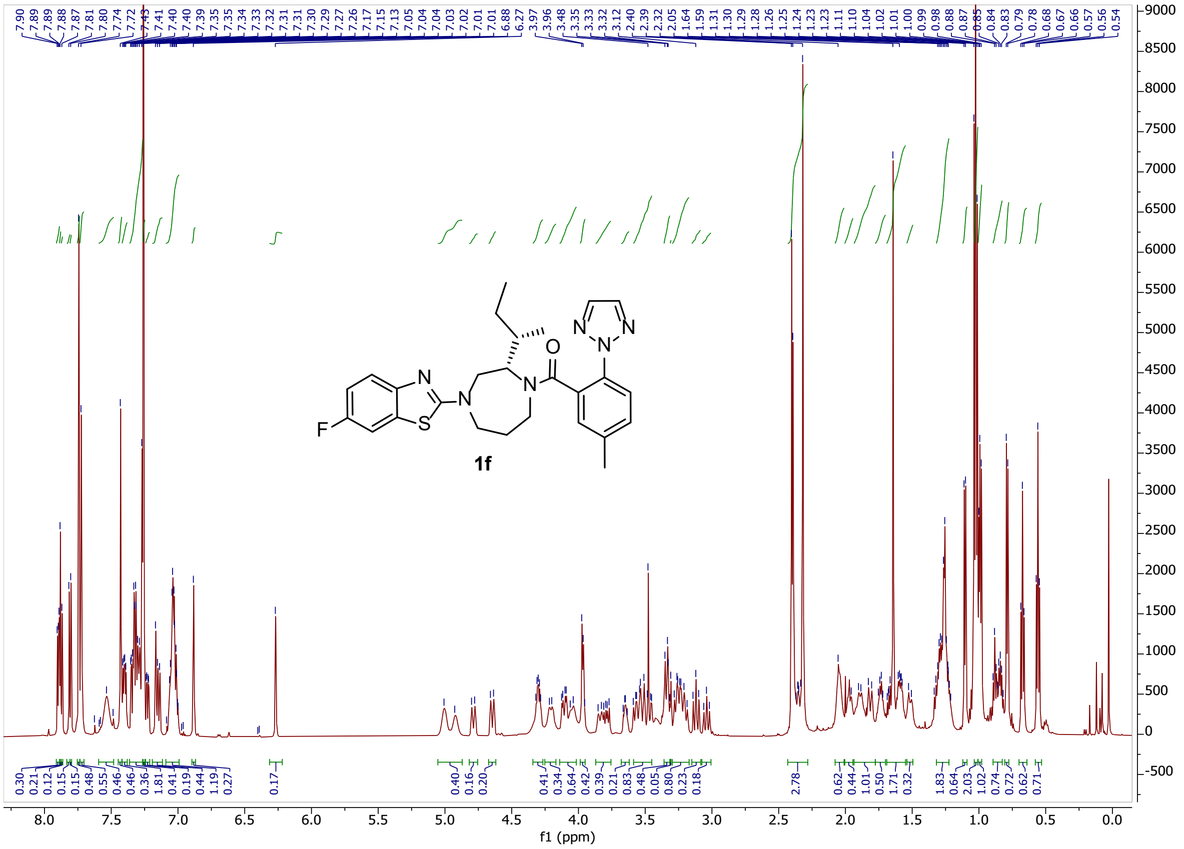

DEPTQ-NMR of **1f**

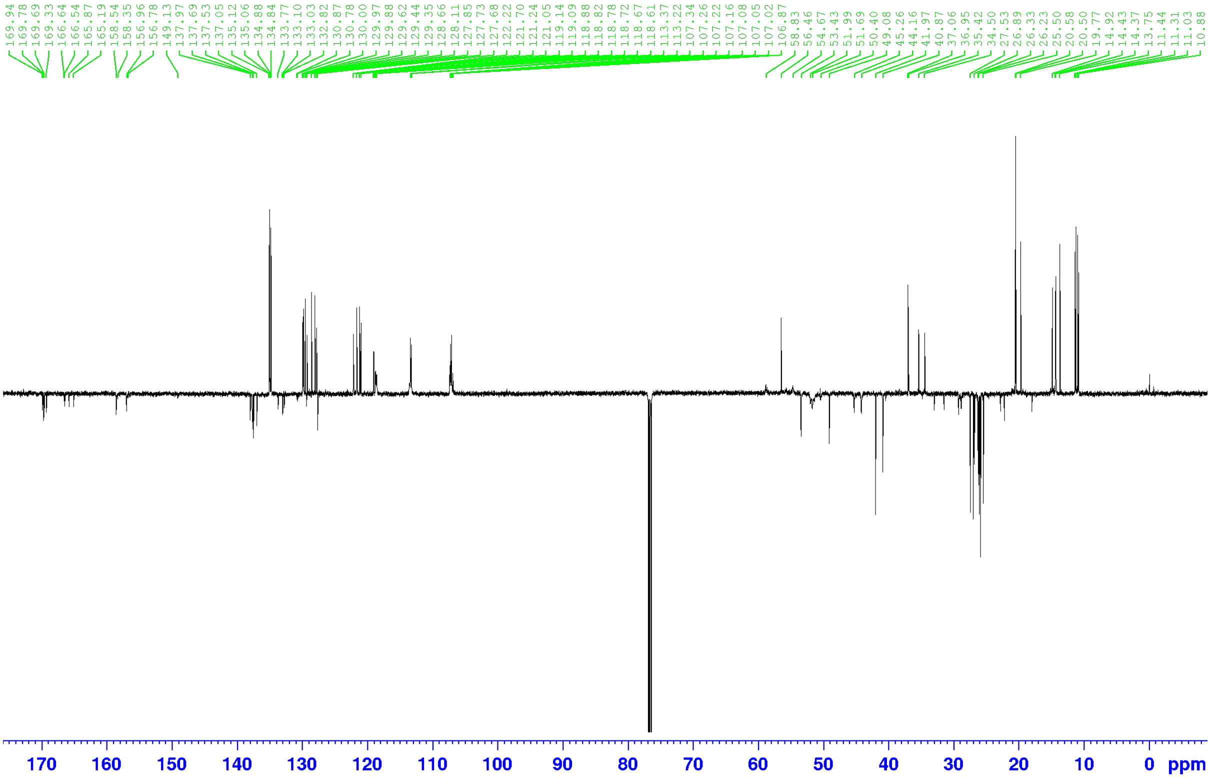

DEPTQ-NMR of **1f** (aromatic region)

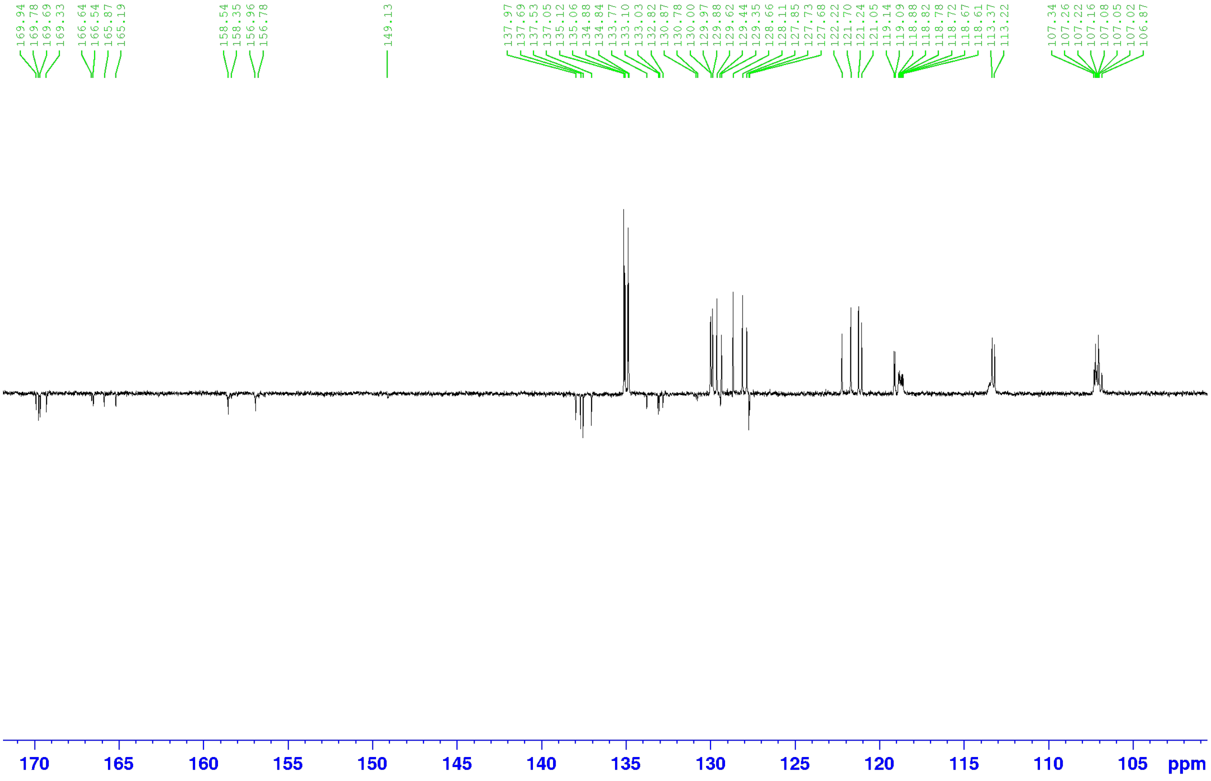

DEPTQ-NMR of **1f** (aliphatic region)

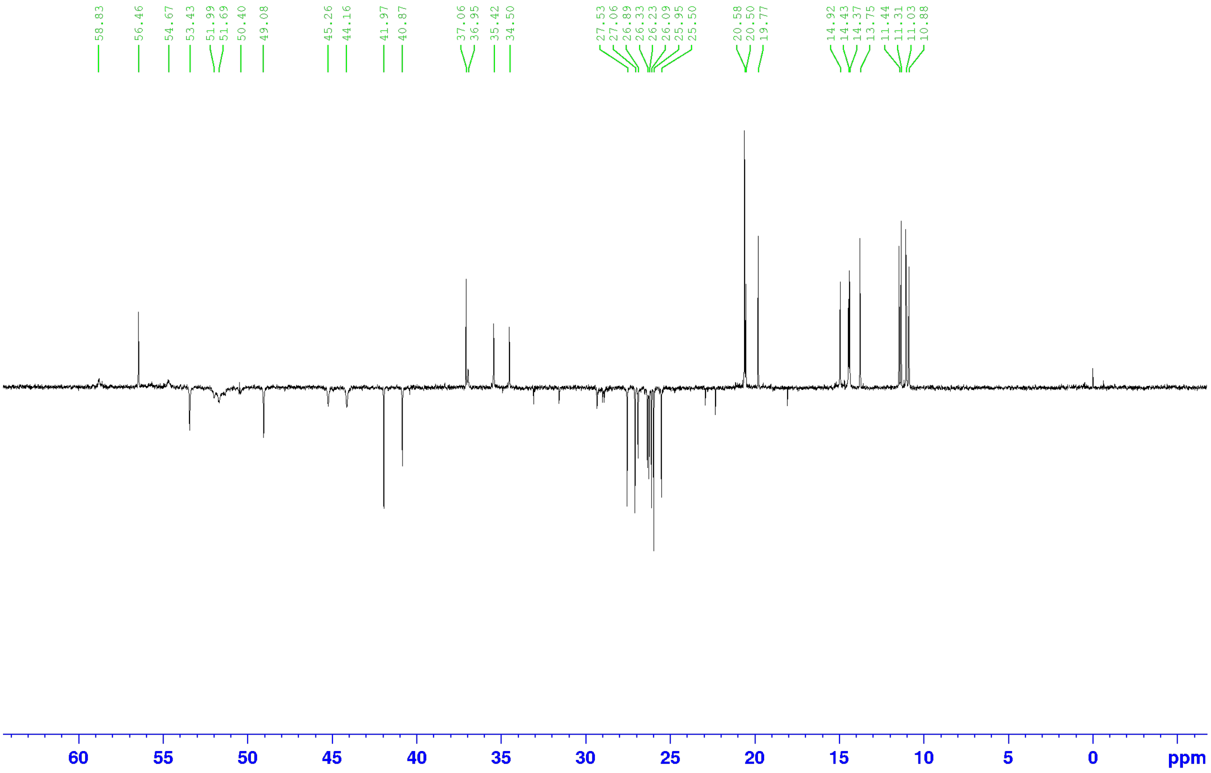

DEPTQ-NMR of **1f** (aliphatic region):

Integration of the resonances of the terminal CH_3-group of the *sec*-butyl substituent gave a summed integral of 0.9454. Percentages of each single peak area were: 21% of the first (left), 31% of the second, 25% of the third, and 23% of the fourth peak (four rotamers of **1f**).

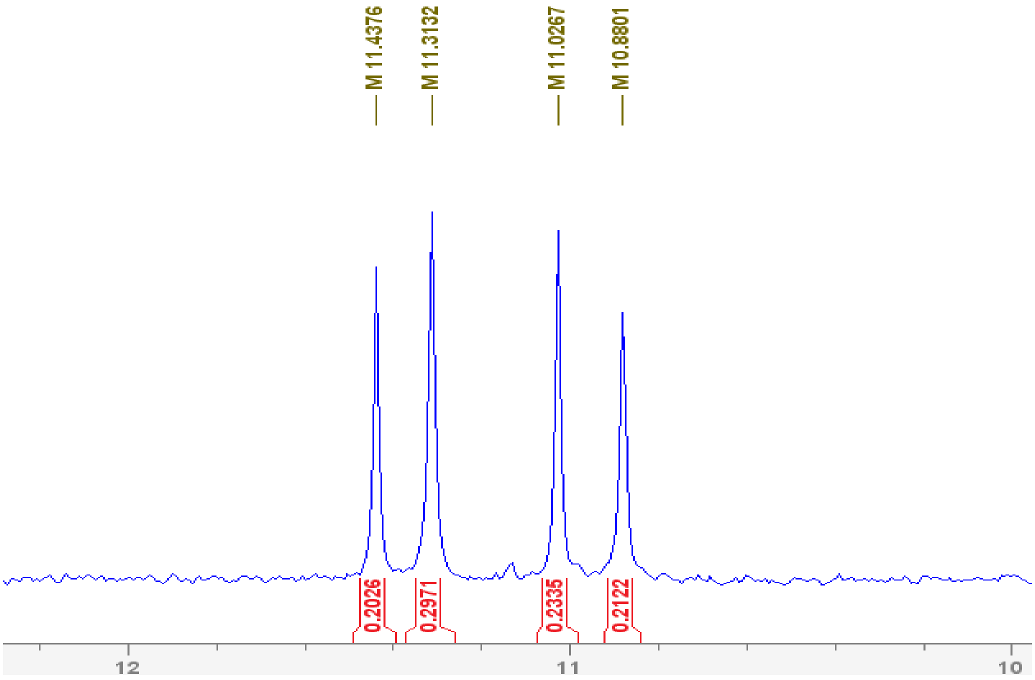

^{1}H-NMR of **2a**

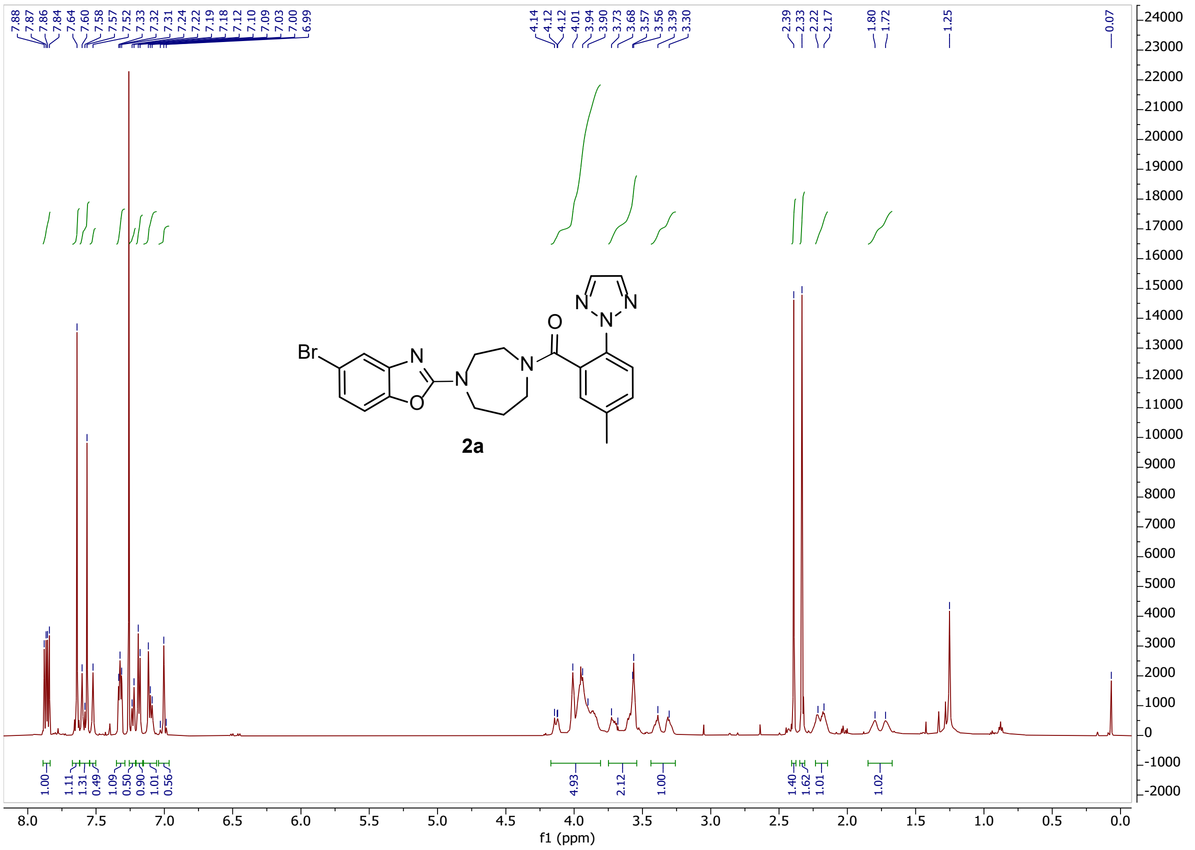

DEPTQ of **2a**

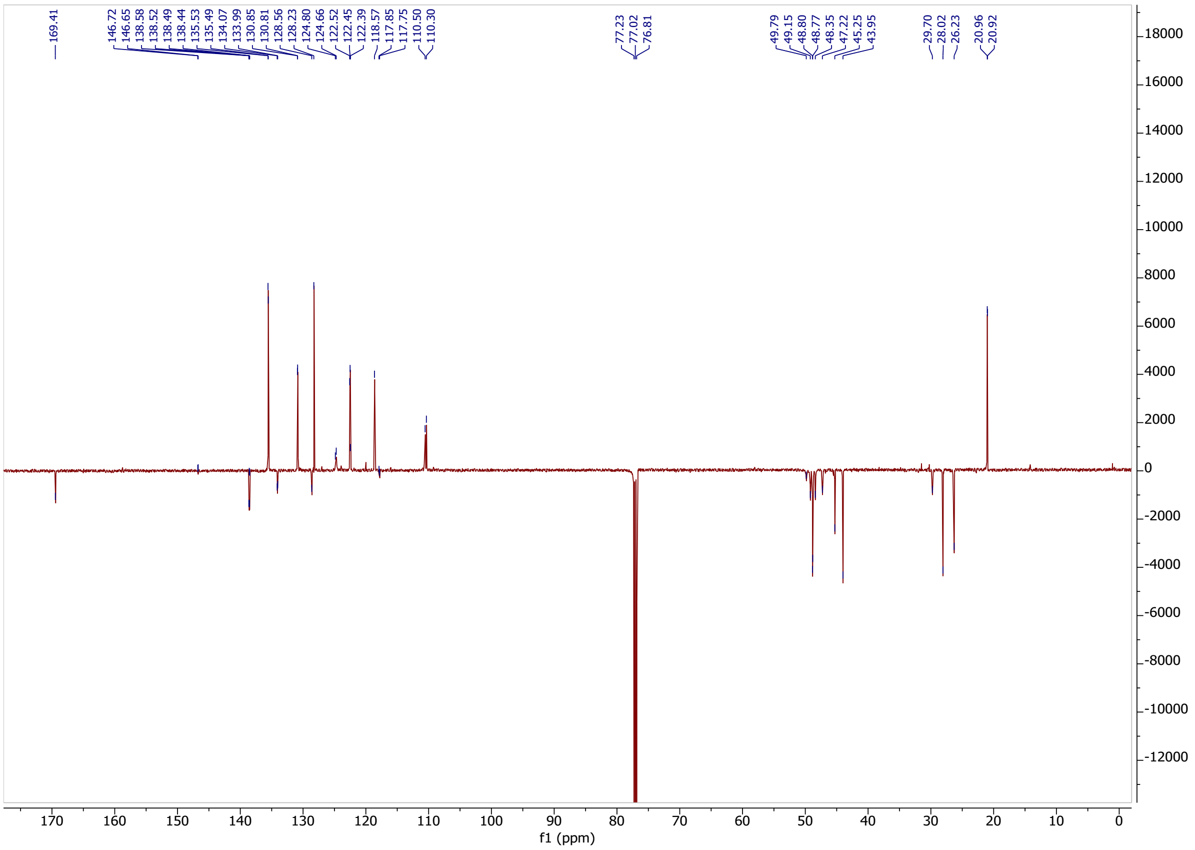

^{1}H-NMR of **2c**

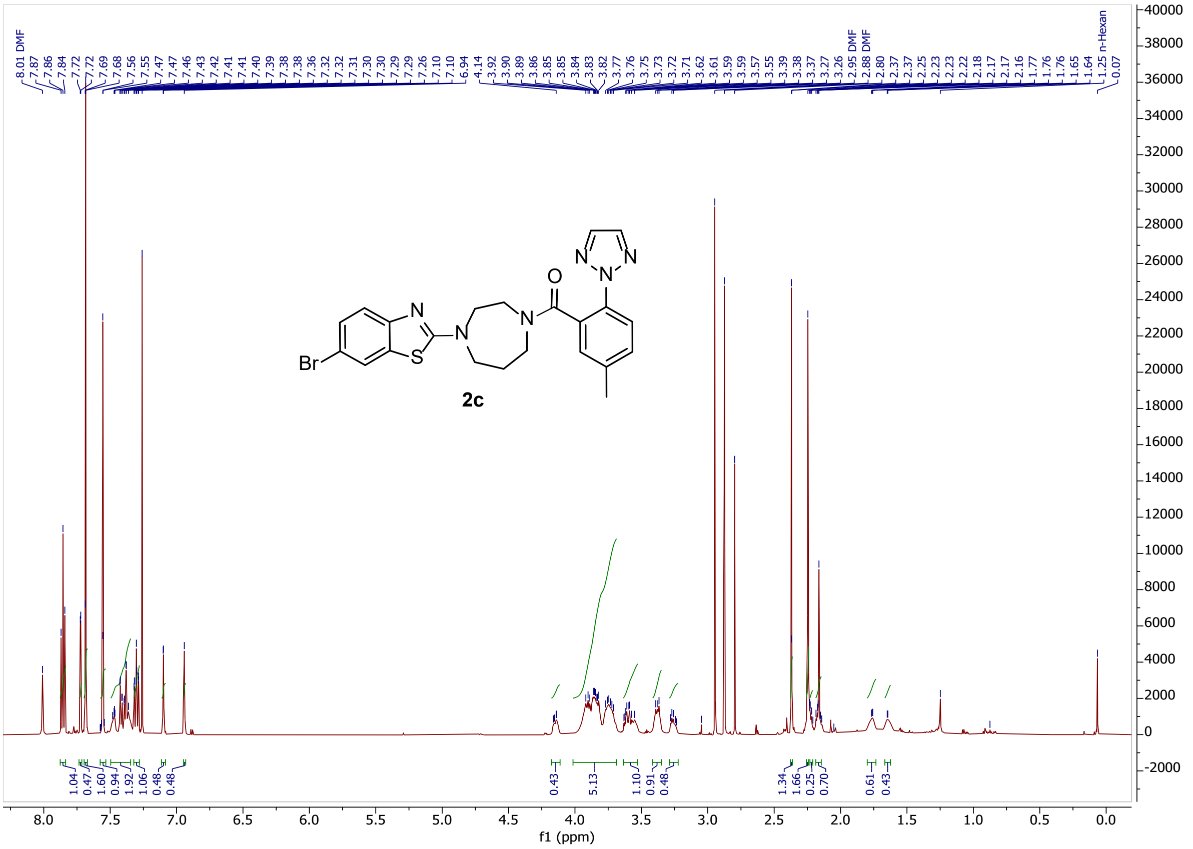

DEPTQ-NMR of **2c**

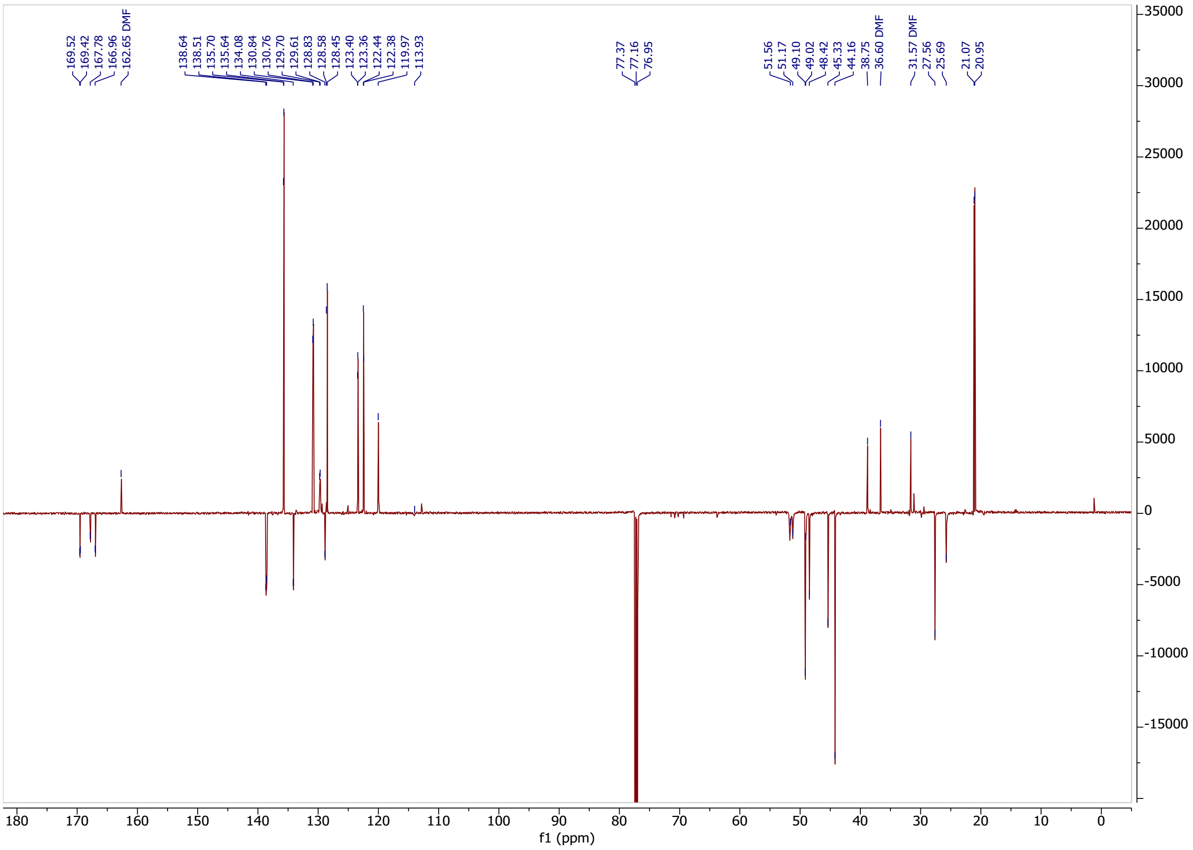

^{1}H-NMR of **2d**

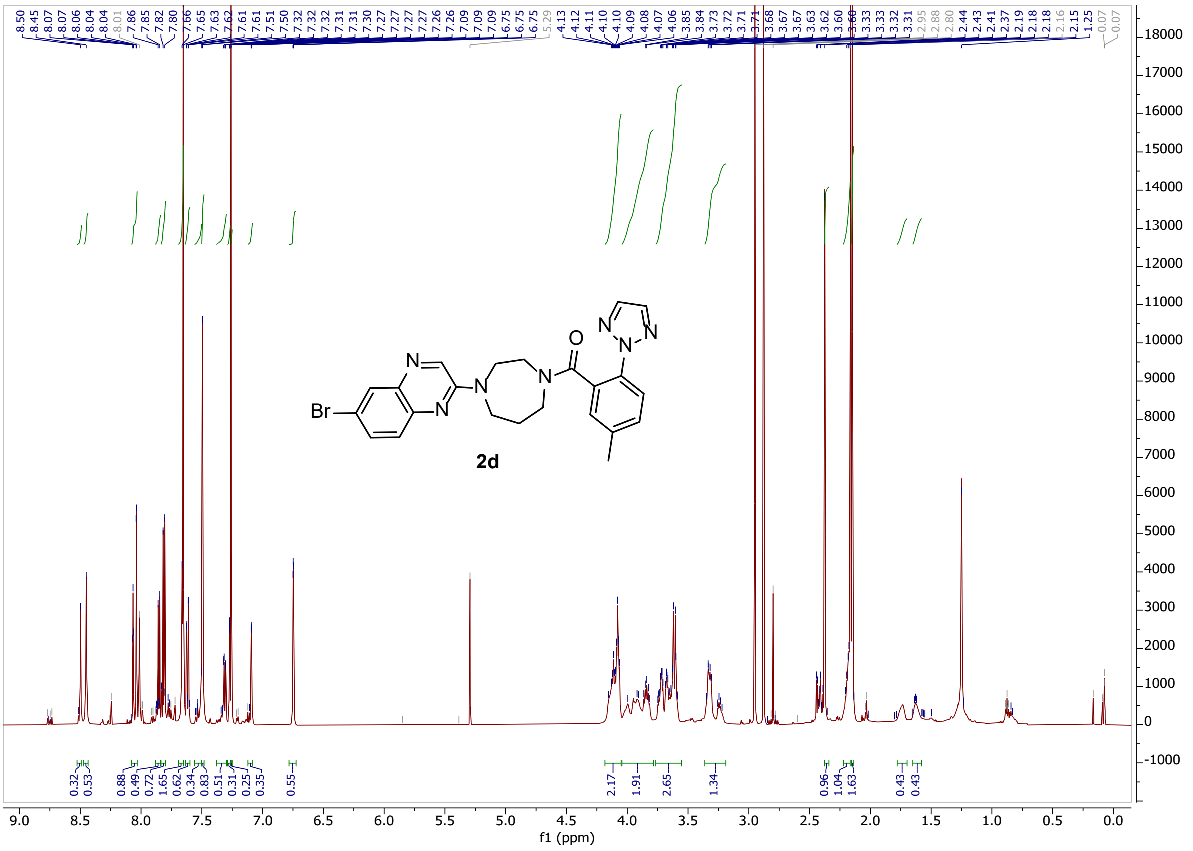

DEPTQ-NMR of **2d**

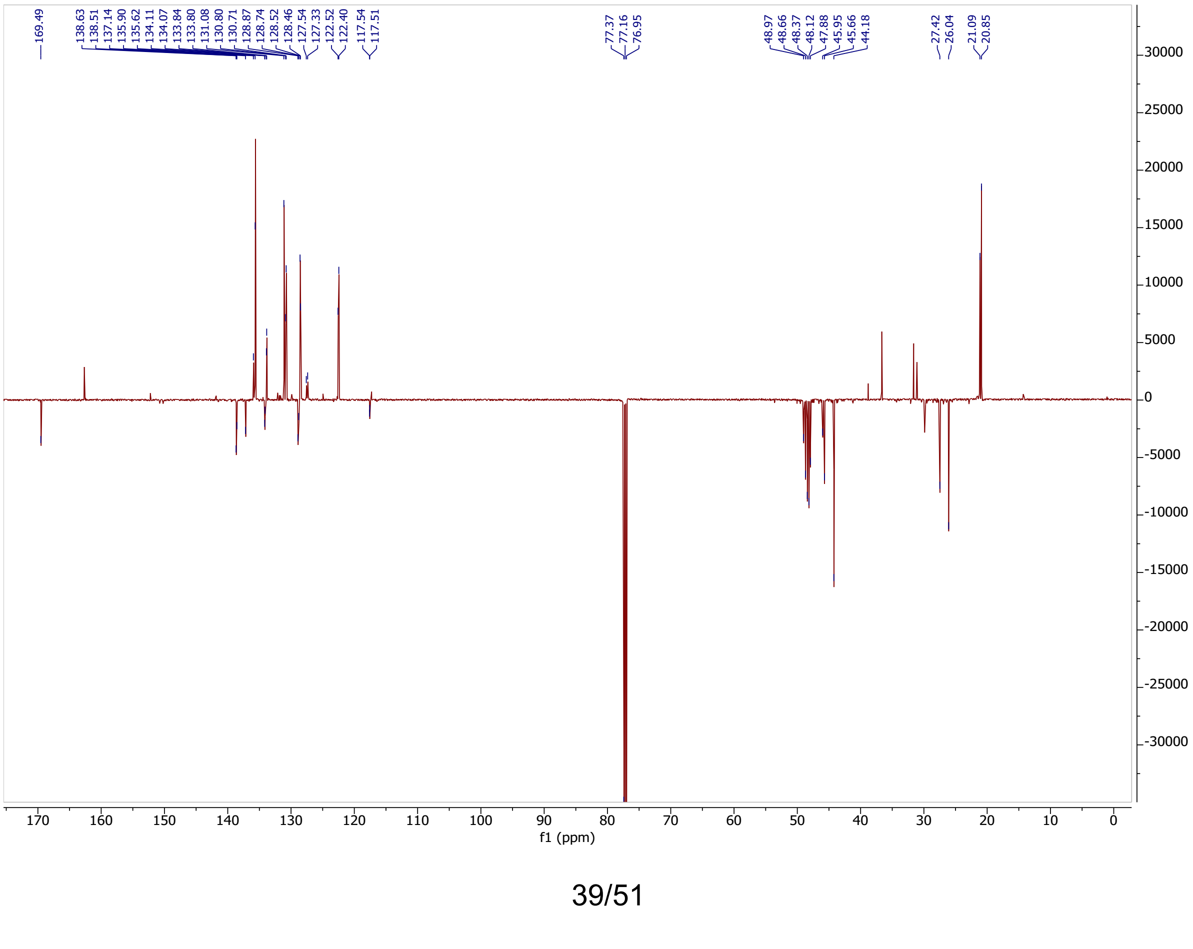

^{1}H-NMR of **2e**

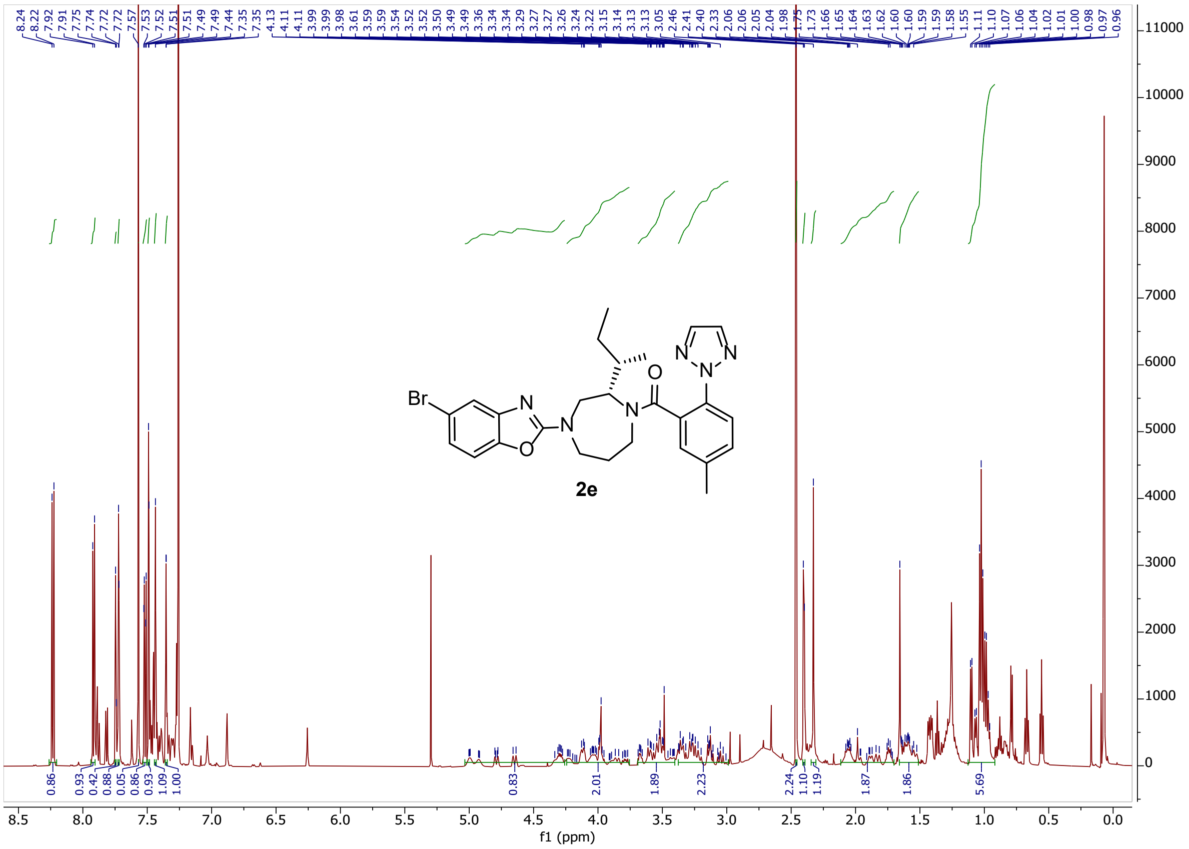

^{1}H-NMR of **3a**

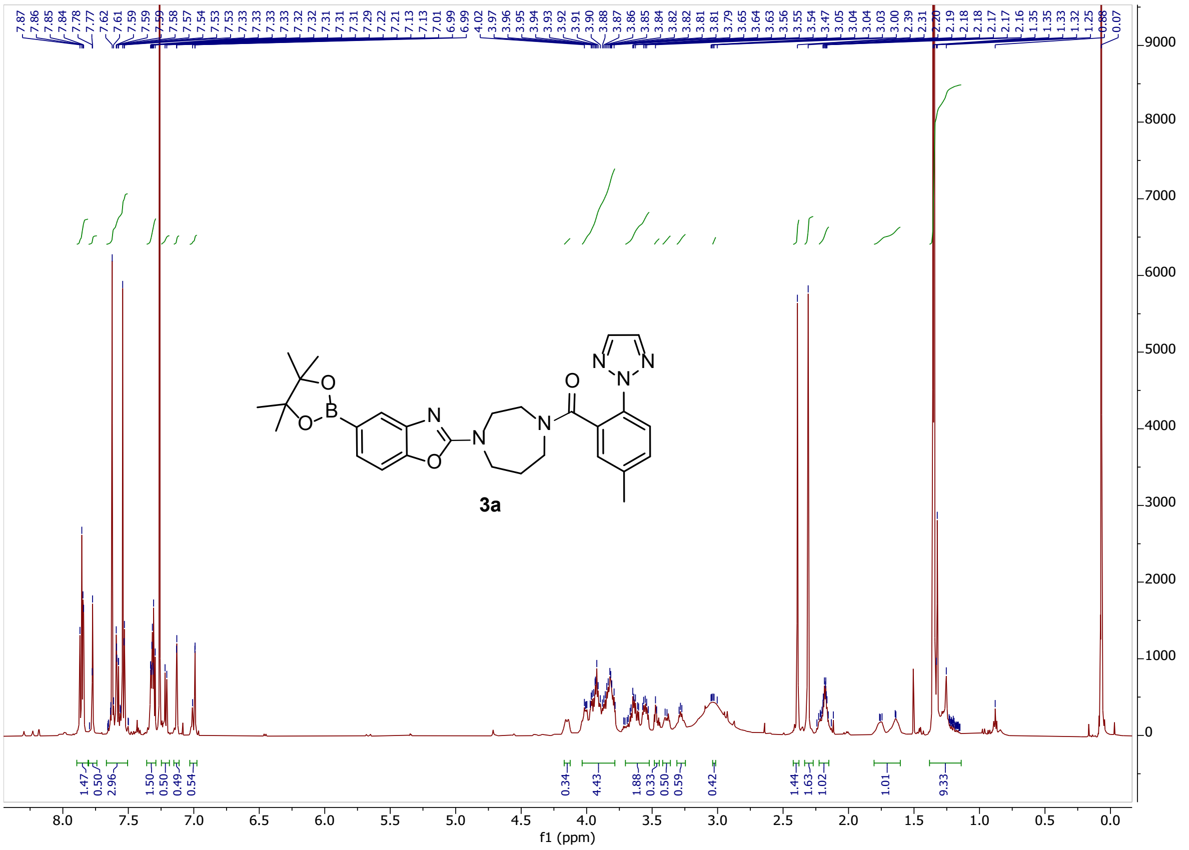

DEPTQ of **3a**

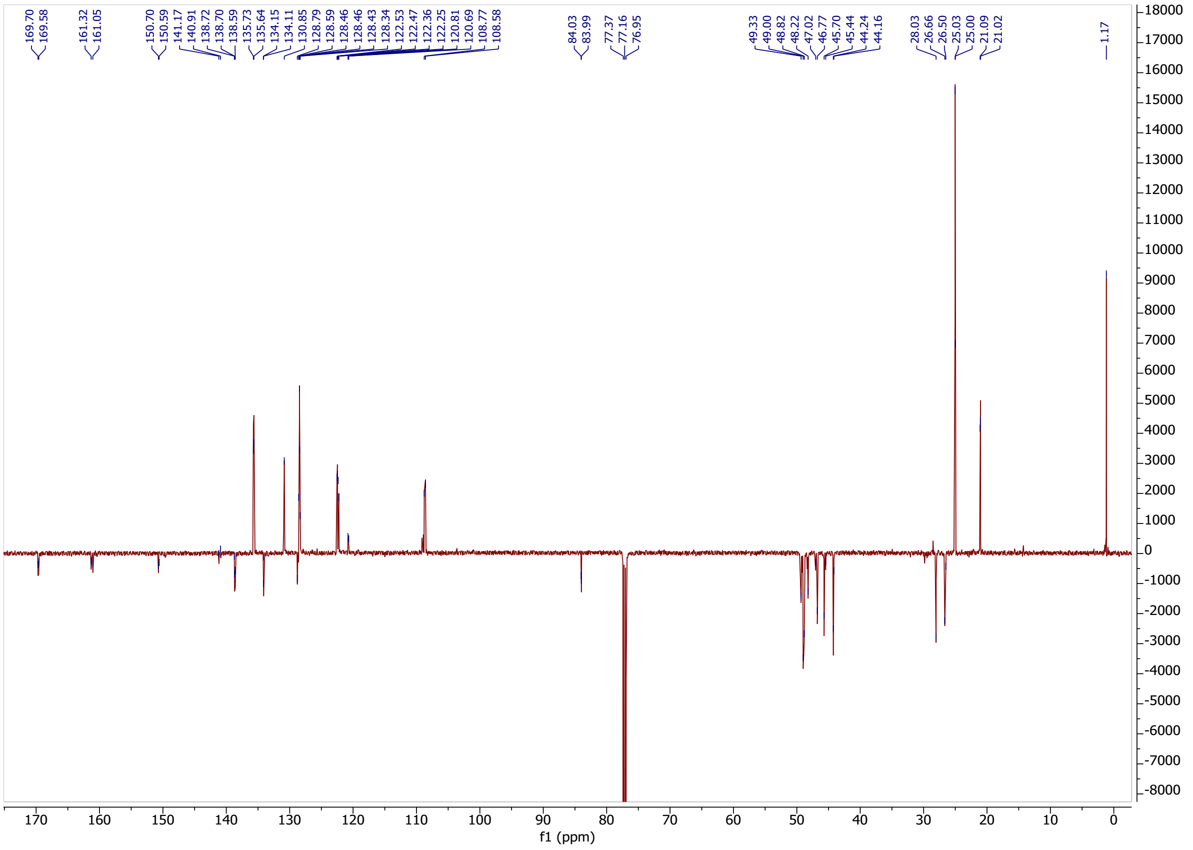

^{1}H-NMR of **3d**

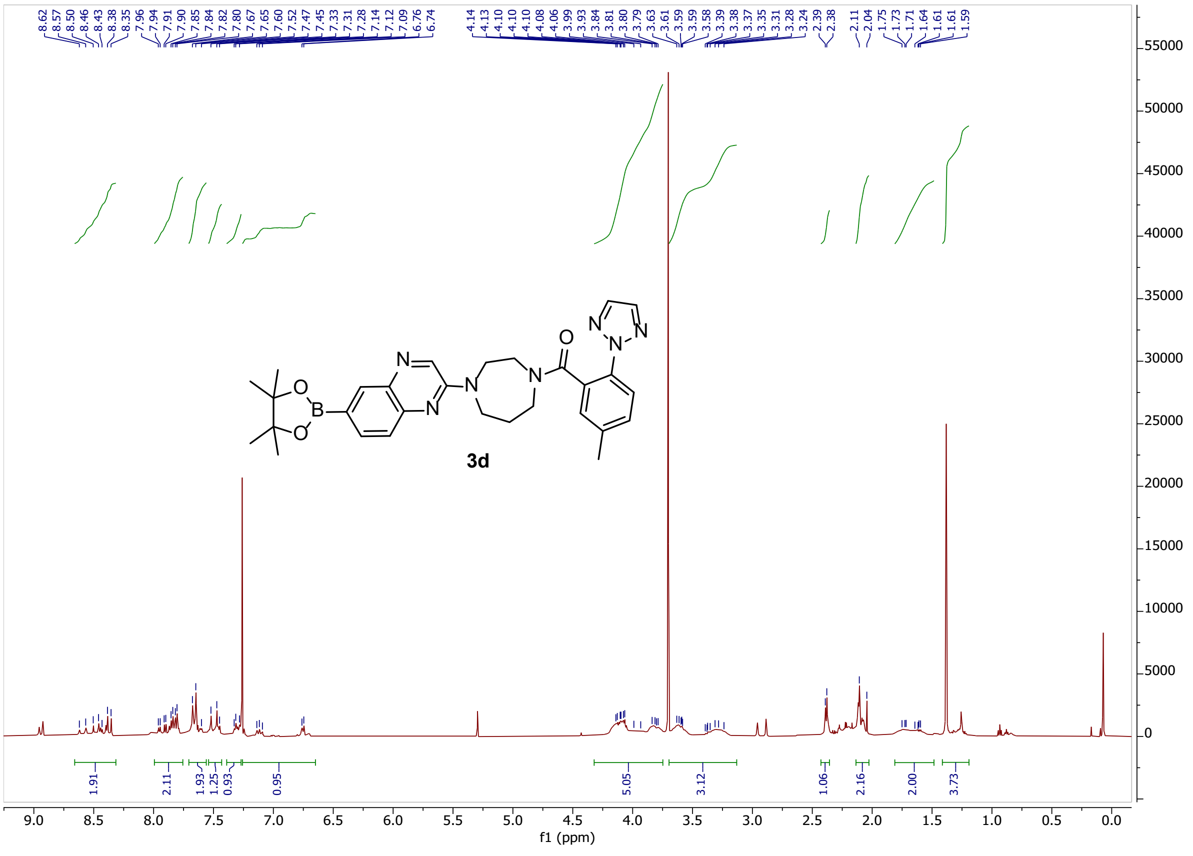

DEPTQ of **3d**

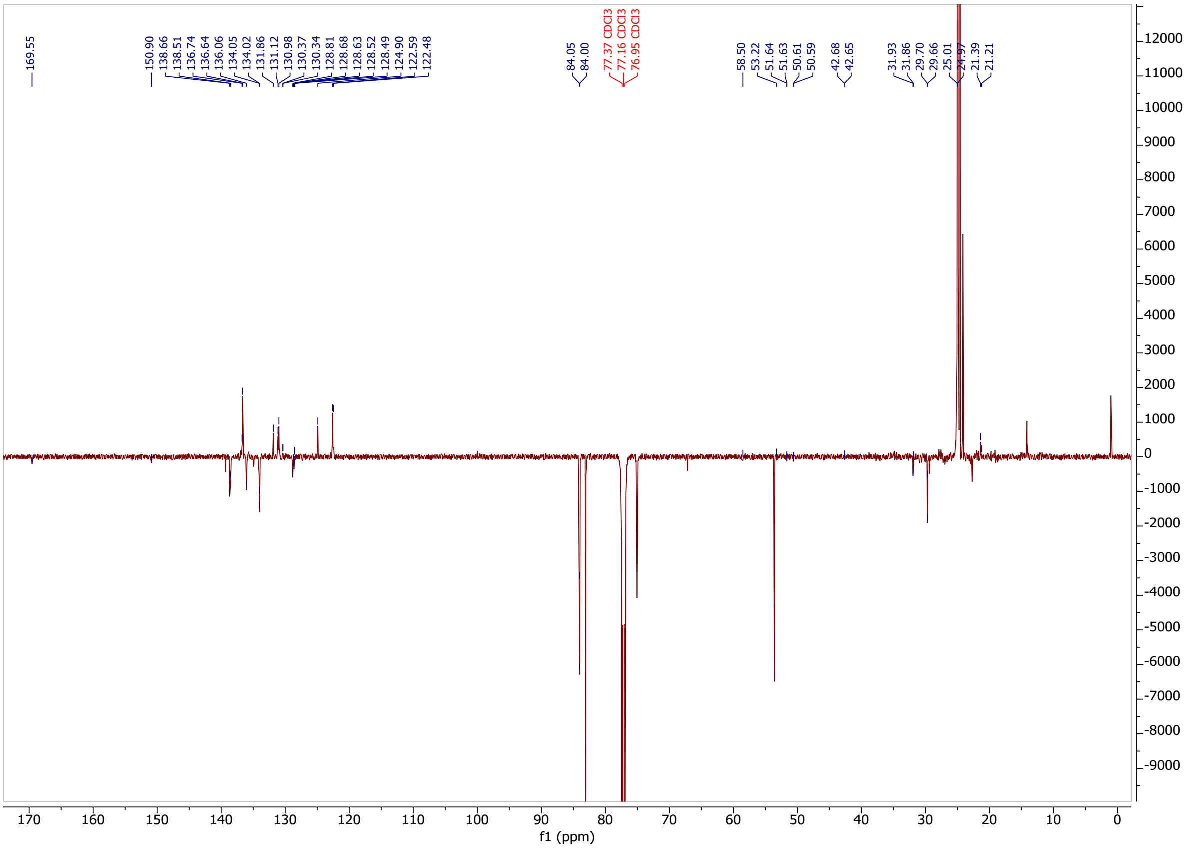

^{1}H-NMR of **4c** (boronic acid intermediate)

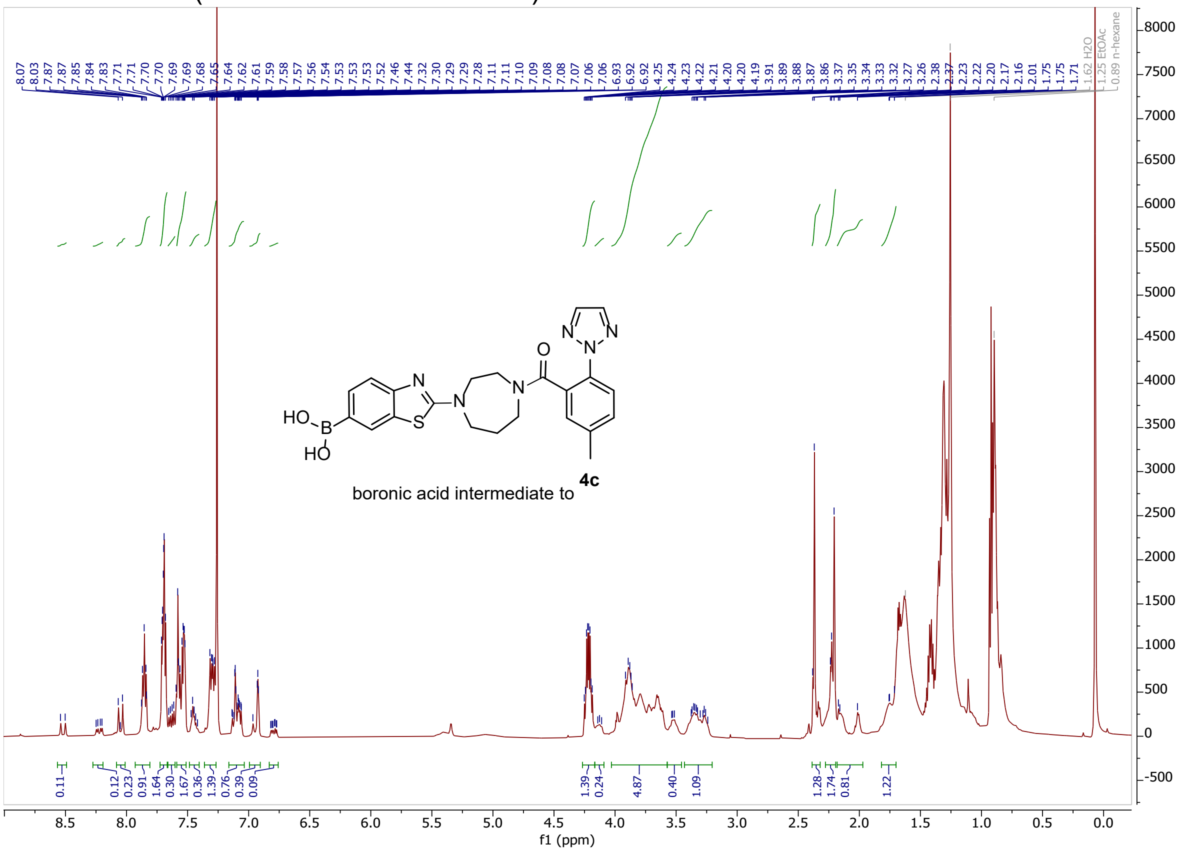

DEPTQ-NMR of **4c** (boronic acid intermediate)

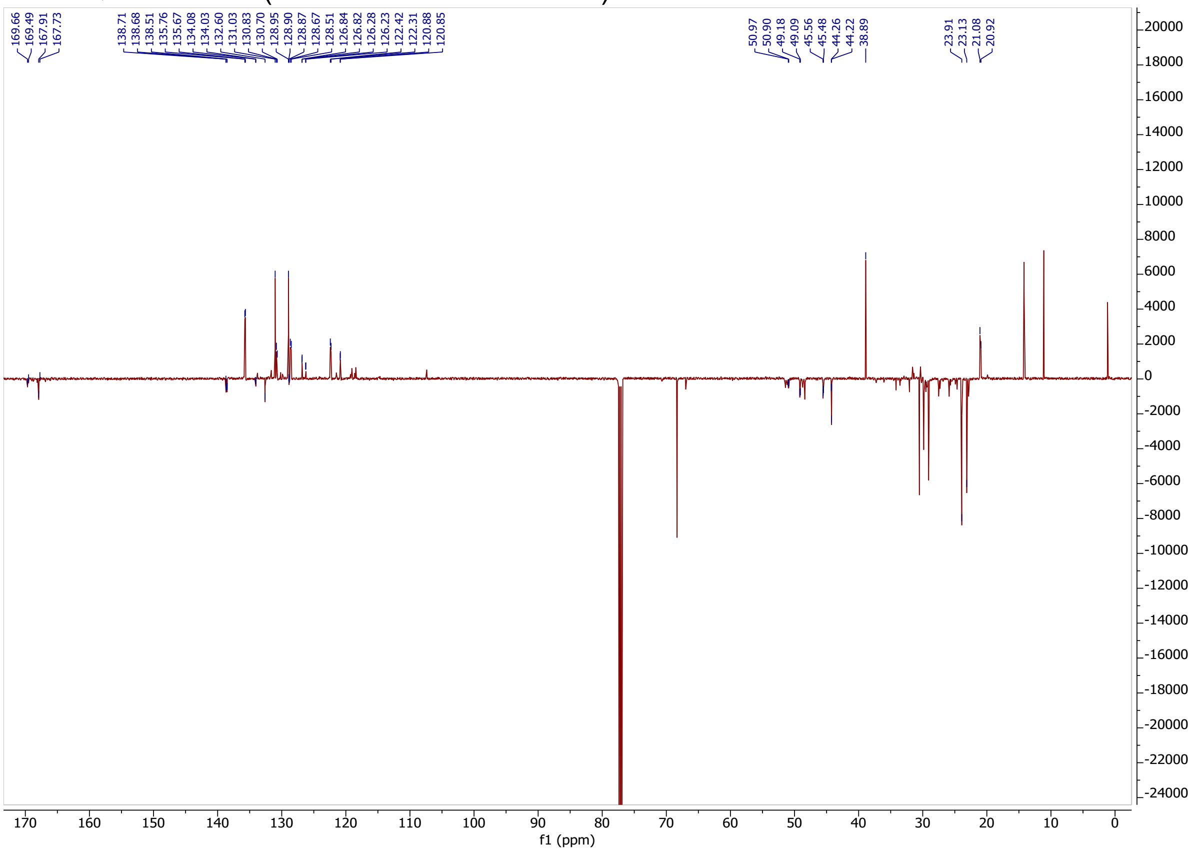

^{1}H-NMR of **7a**

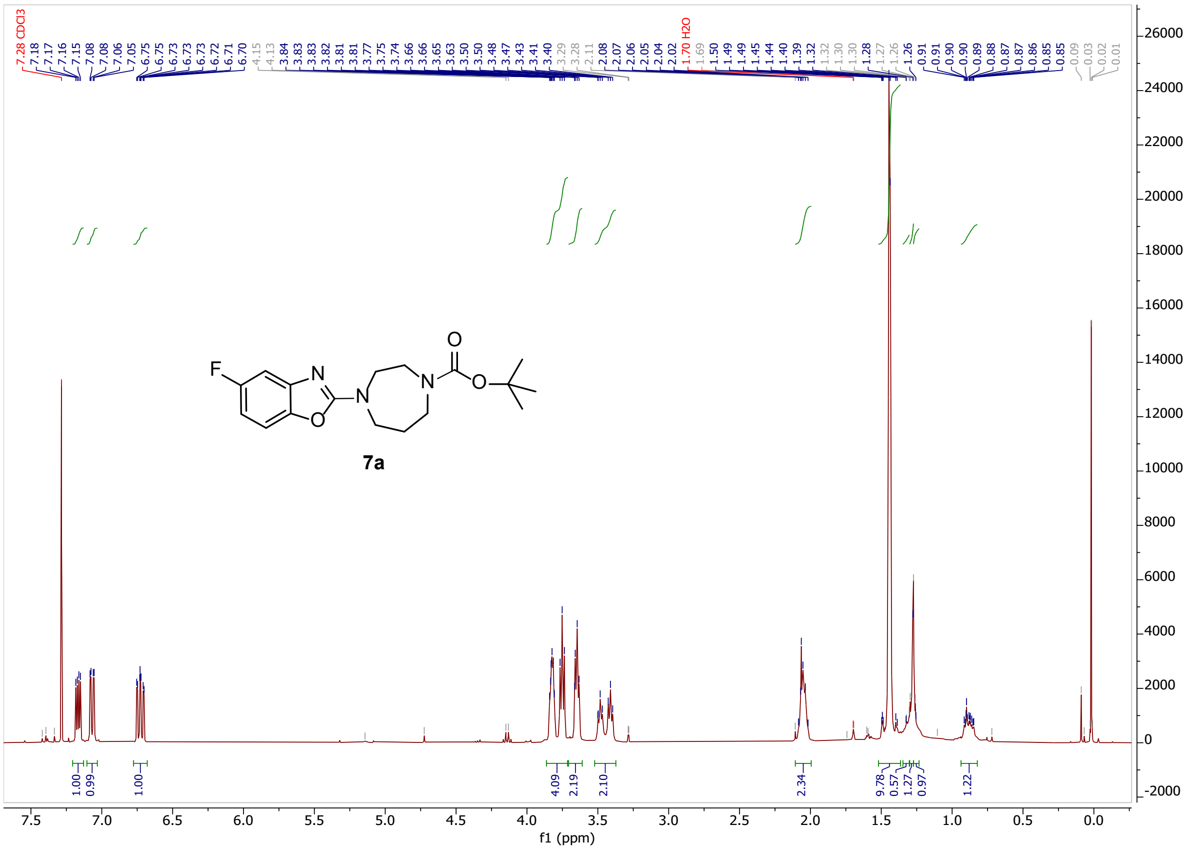

DEPTQ-NMR of **7a**

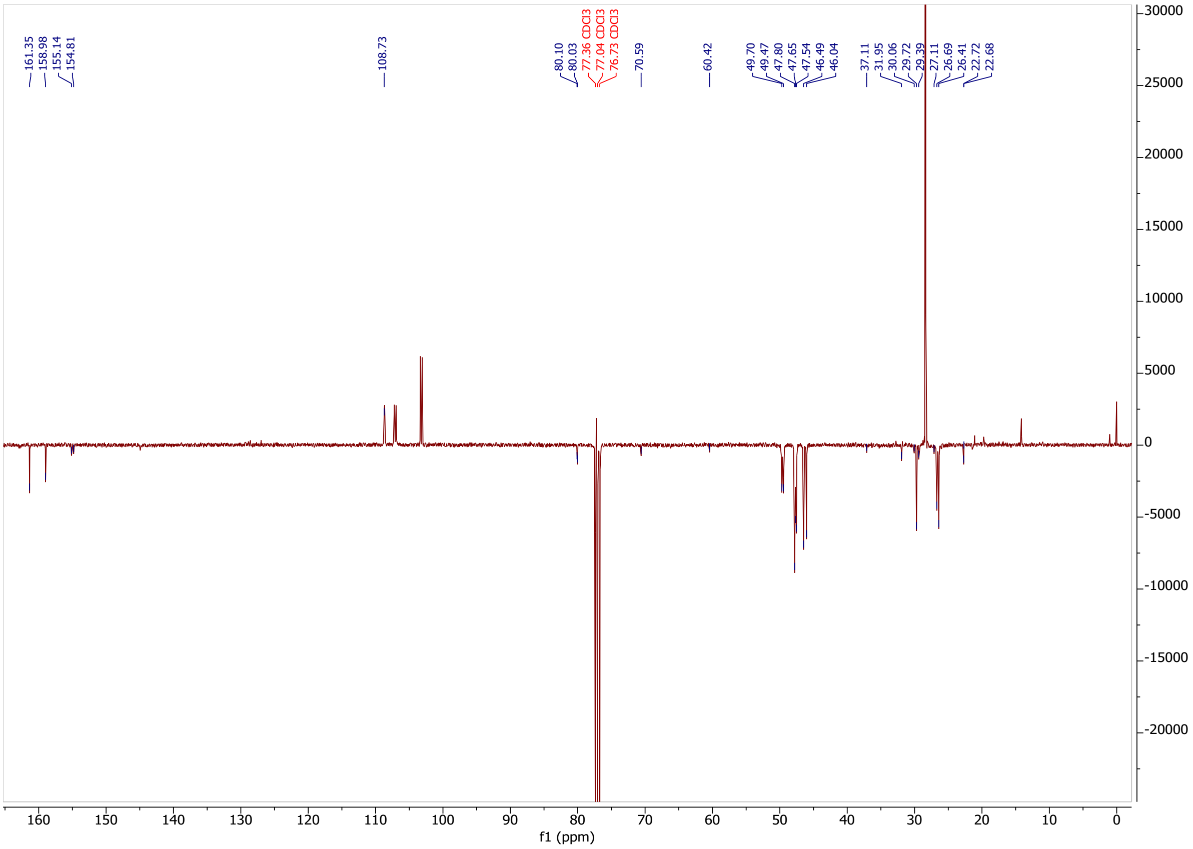

^{1}H-NMR of **8a**

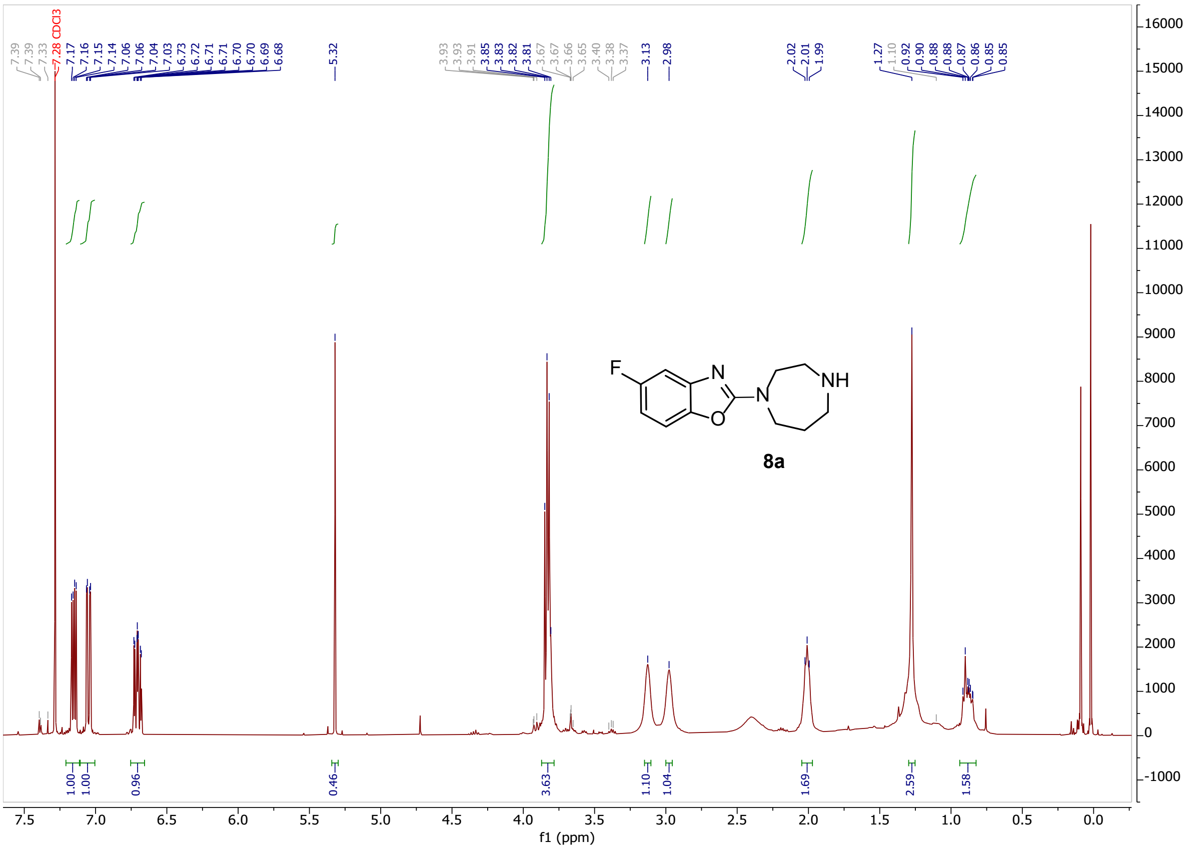

DEPTQ-NMR of **8a**

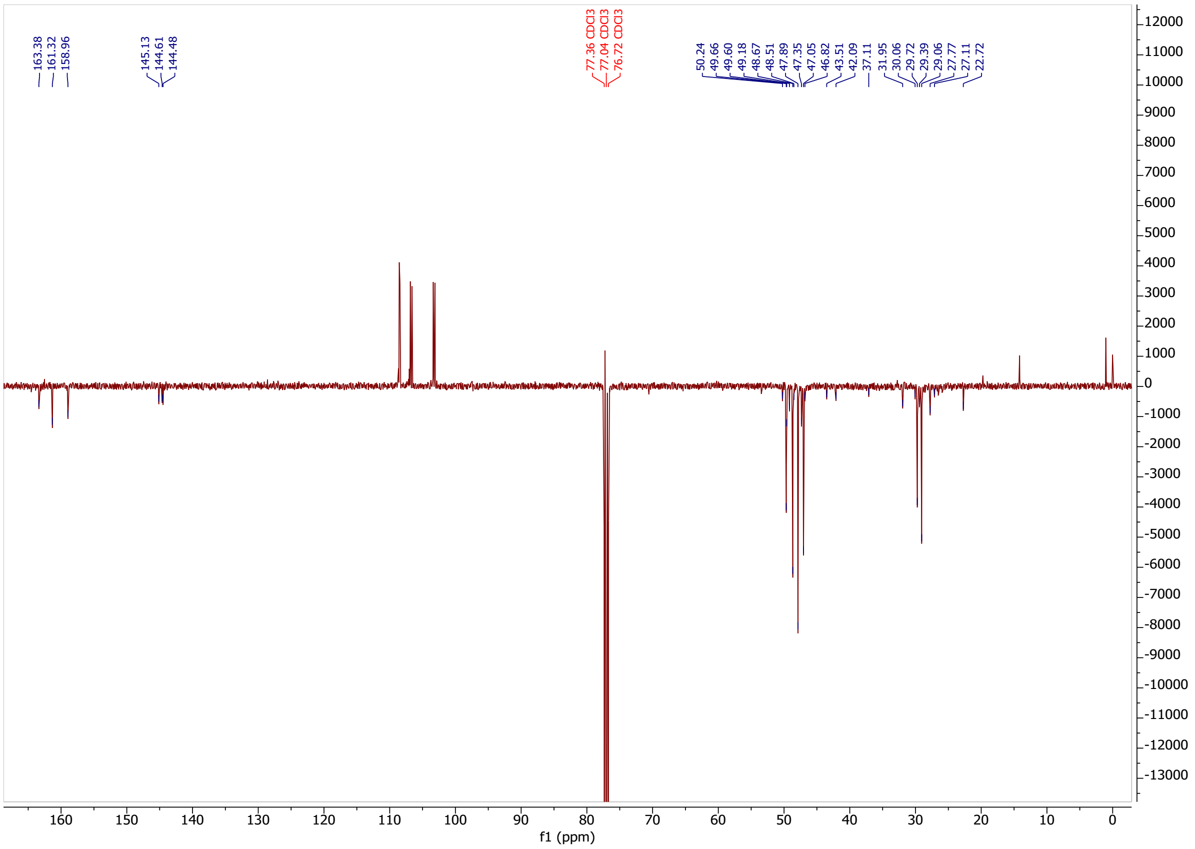

^{1}H-NMR of **9a**

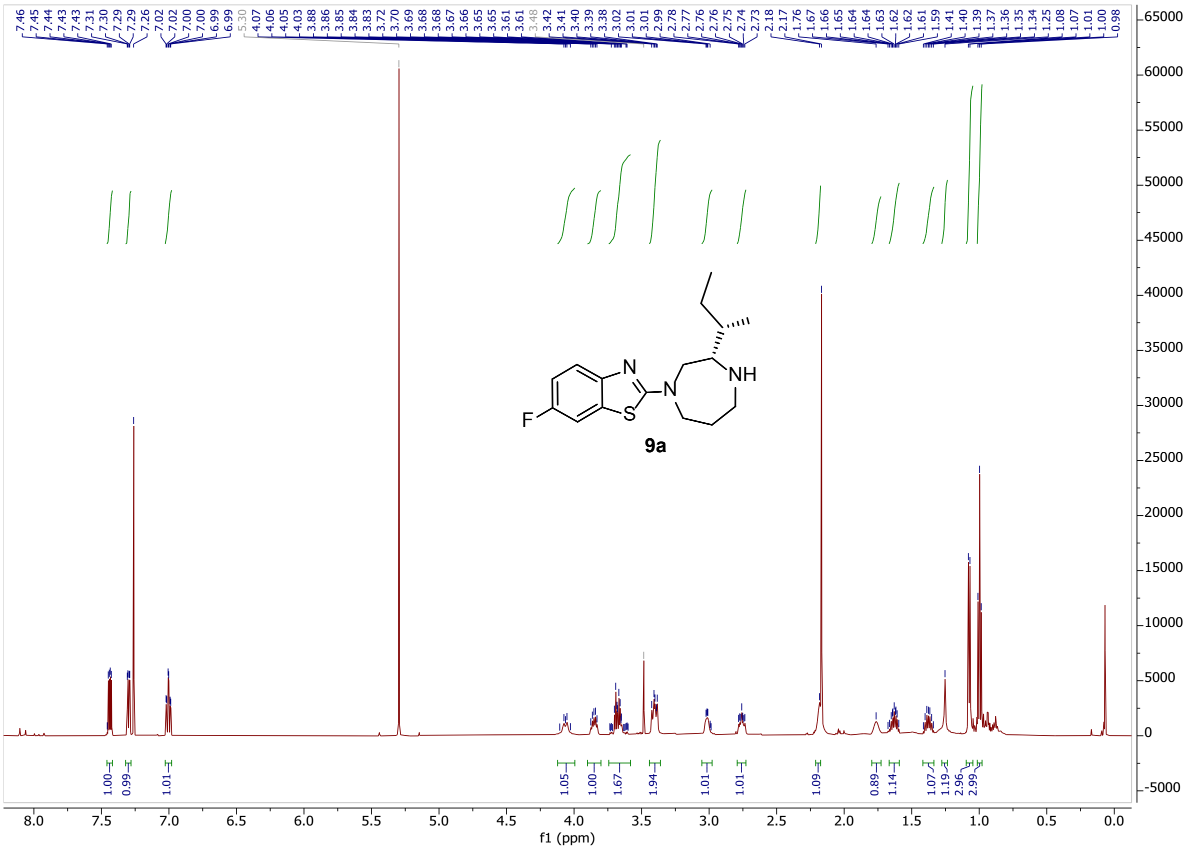

^{1}H-NMR of **10**

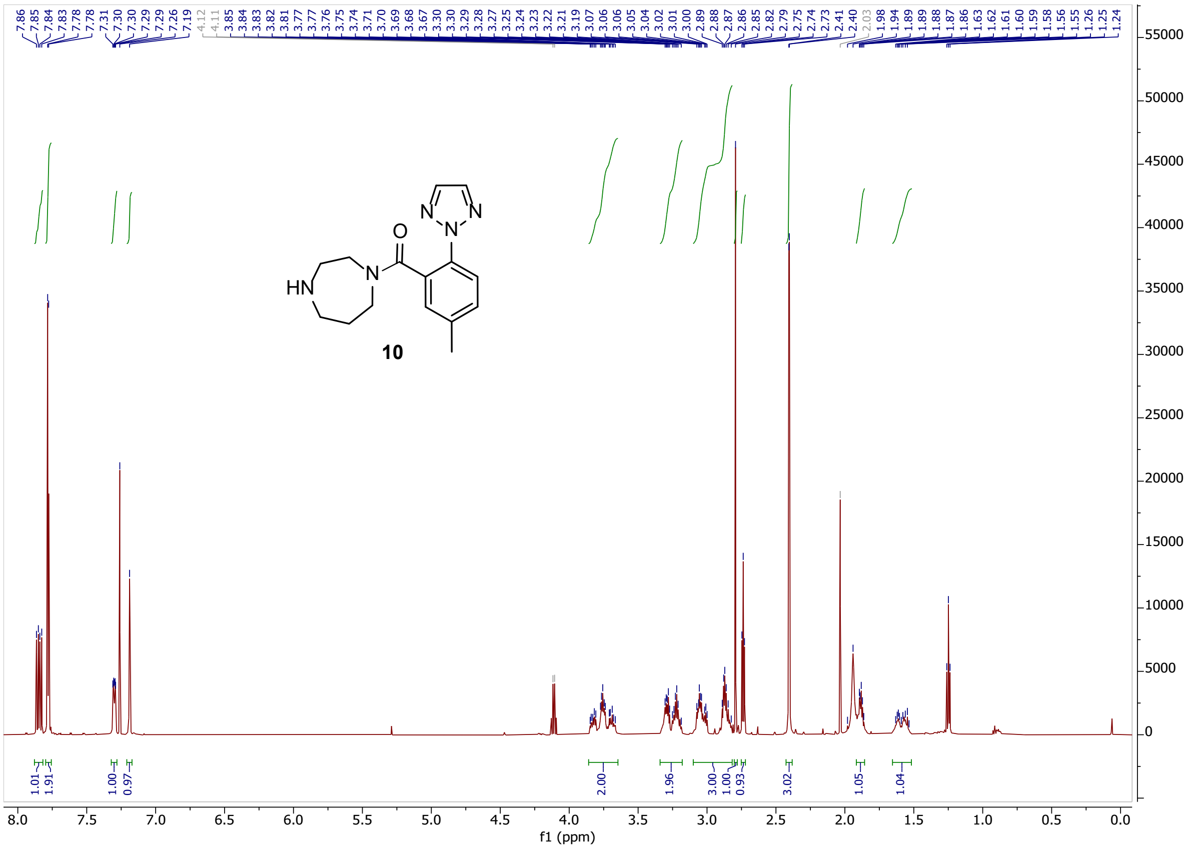

HPLC analysis of compounds 1a-1f (see Materials and methods for HPLC conditions)

Purity of **1a** (eluent system 4)

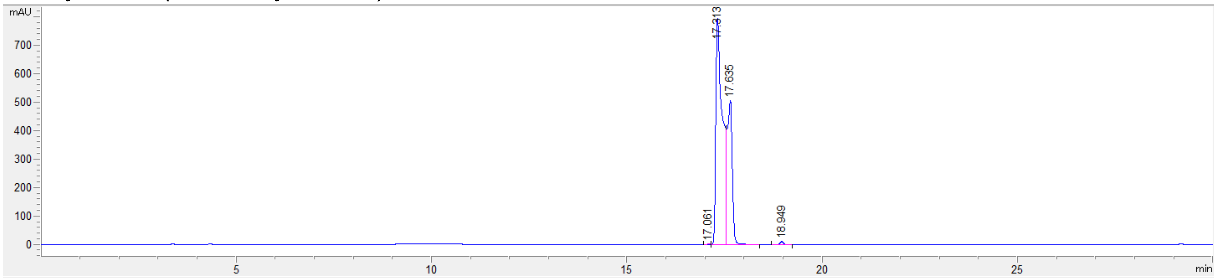

Purity of **1b**

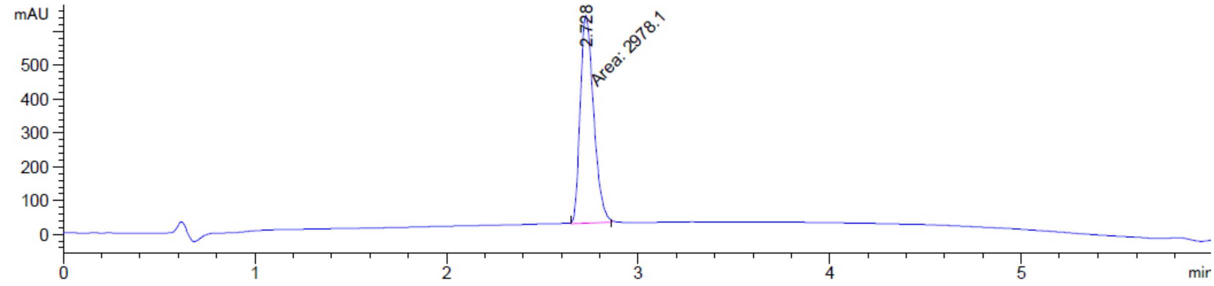

Purity of **1c**

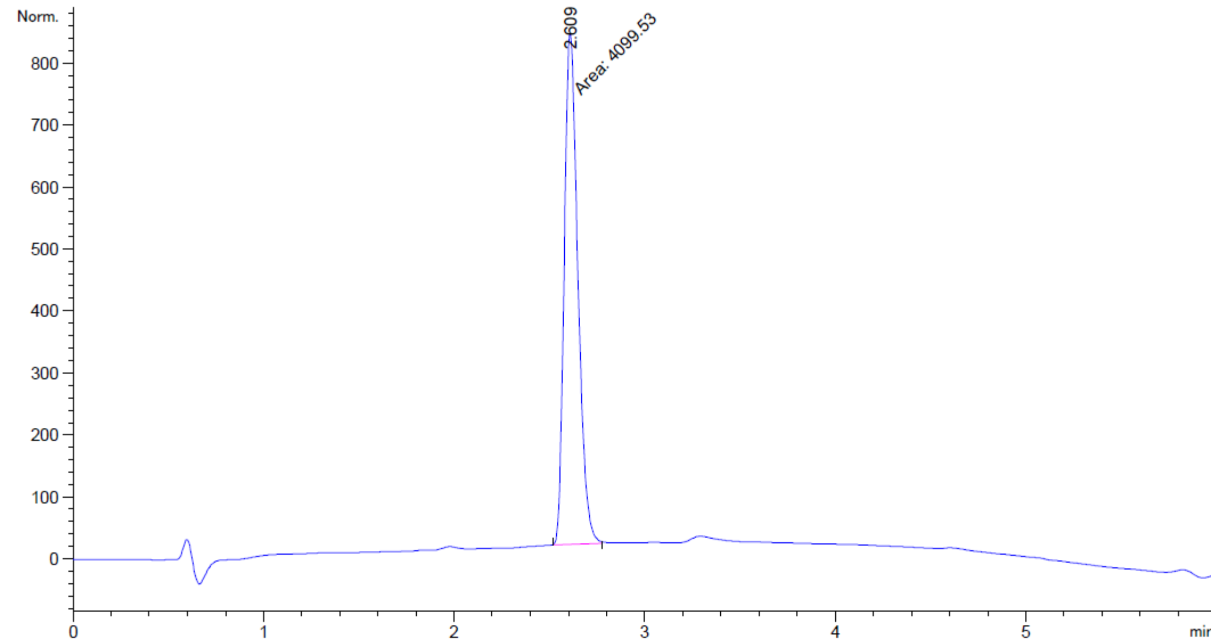

Purity of **1d**

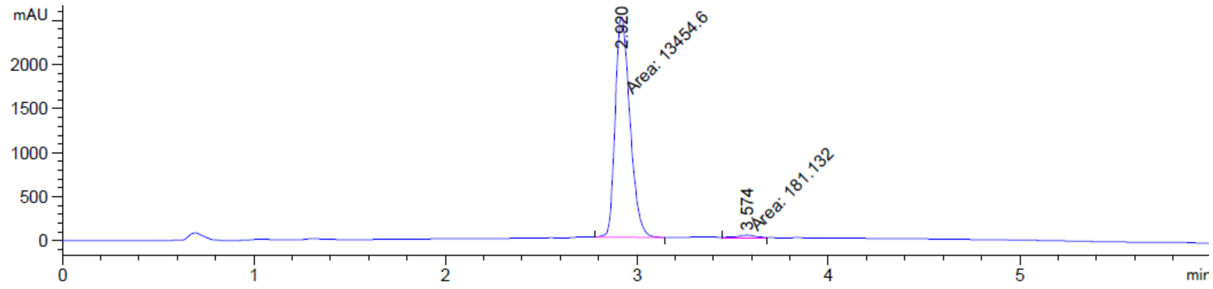

Purity of **1e** (eluent system 4)

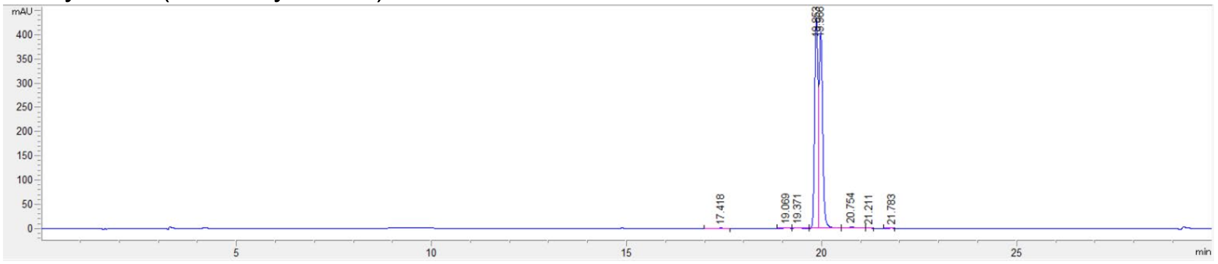

Purity of **1f**

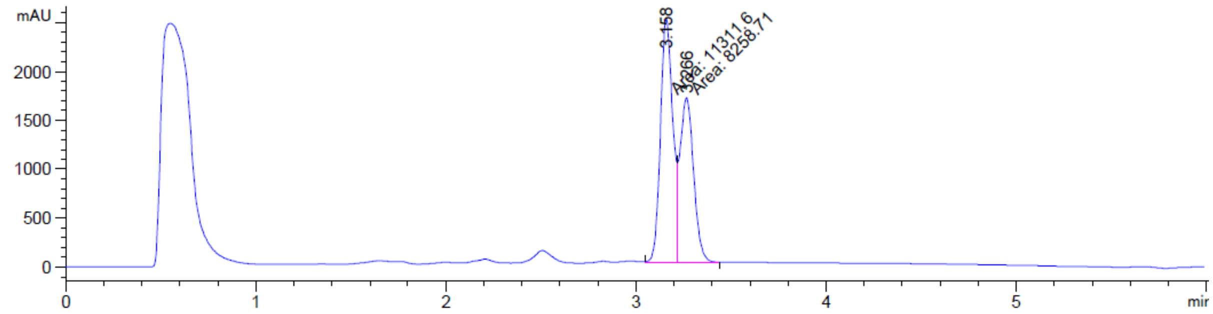

HPLC analysis of ^{18}F-labeled compounds by coinjection of non-radioactive reference compounds (see Materials and methods for HPLC conditions)

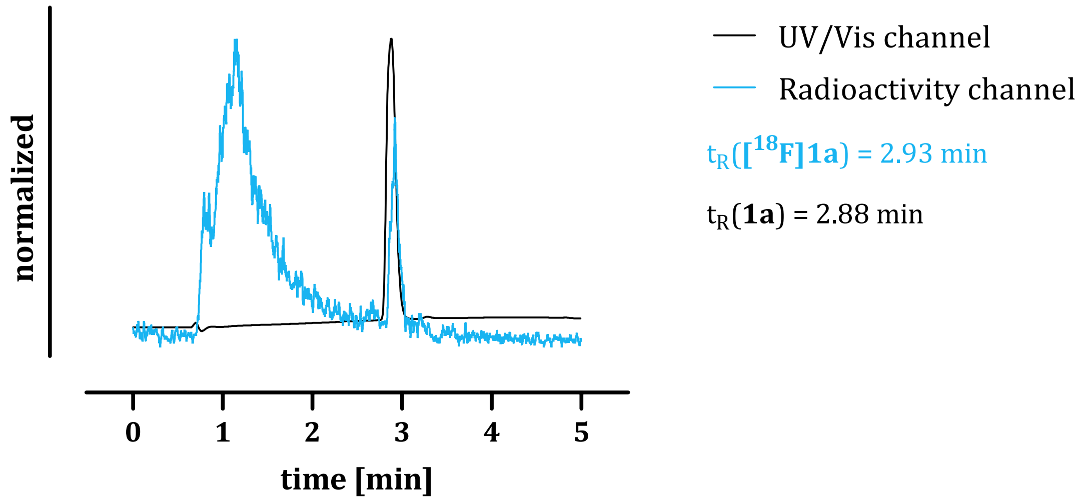

Figure S5. HPLC Coinjection of **1a** and **[^{18}F]1a** (sample drawn from the reaction after 20 min).

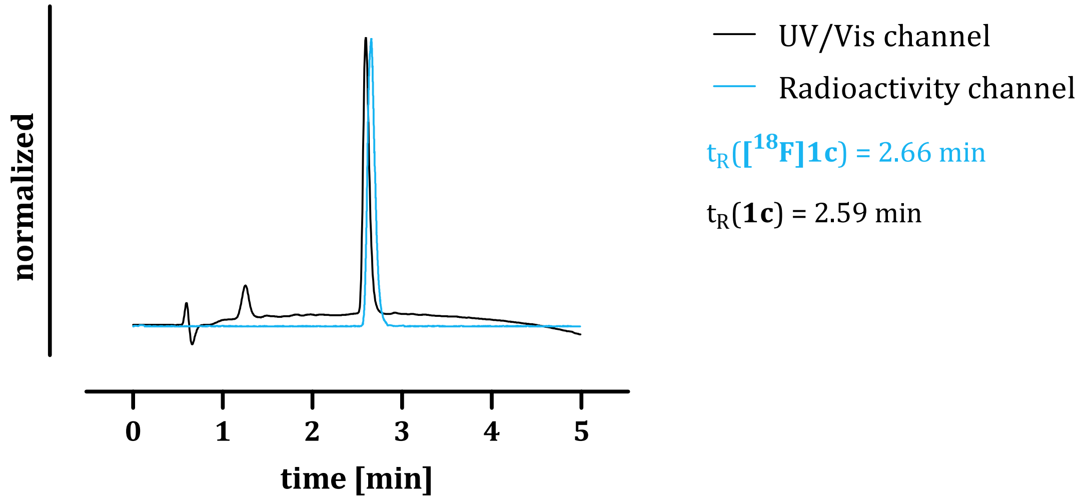

Figure S6. HPLC Coinjection of **1c** and **[^{18}F]1c** (isolated product).

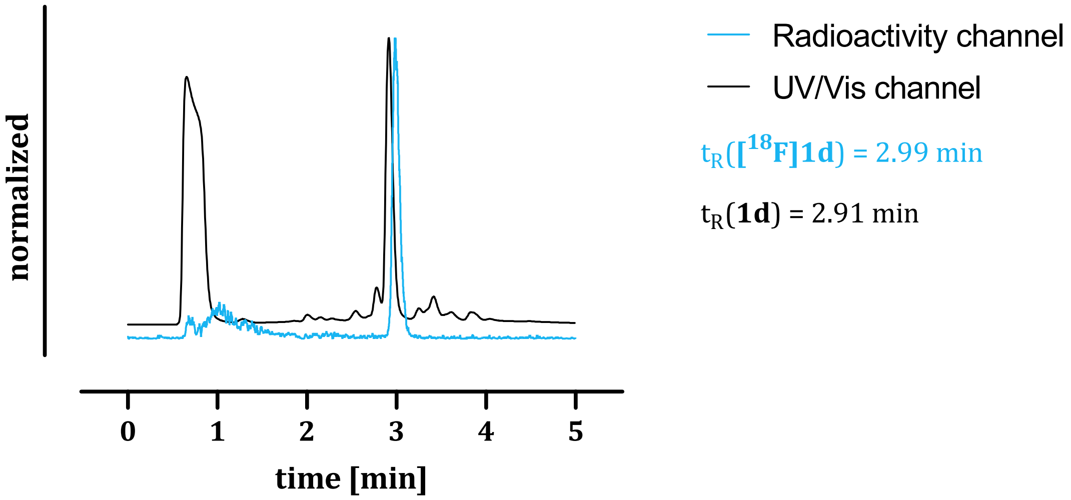

Figure S7. HPLC Coinjection of **1d** and **[^{18}F]1d** (sample drawn from the reaction after 10 min).

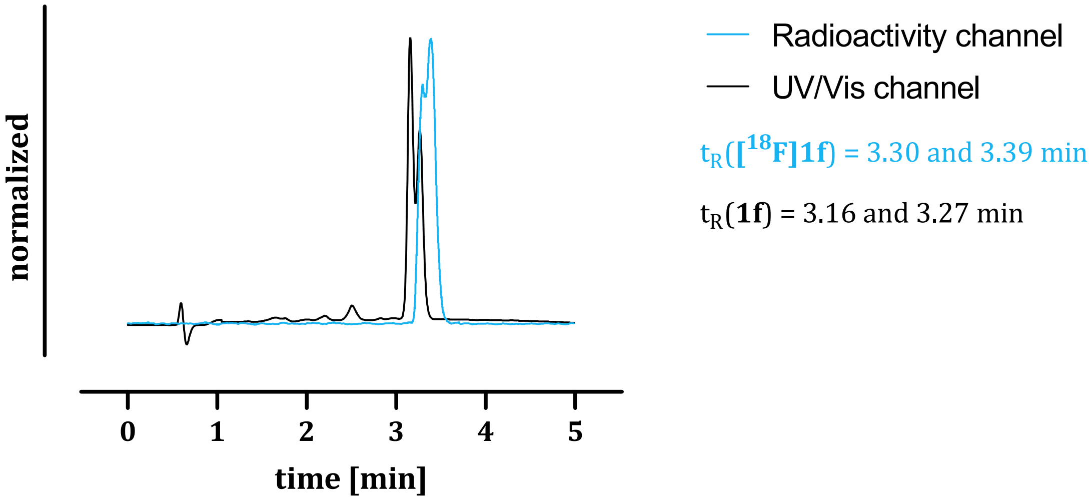

Figure S8. HPLC Coinjection of **1f** and **[^{18}F]1f** (isolated product).

References

1. Hore S, Srivastava A, Singh RP. Cu-Catalyzed Direct C-P Bond Formation through Dehydrogenative Cross-Coupling Reactions between Azoles and Dialkyl Phosphites. J Org Chem. 2019;84(11):6868-78.

2. Hellmann J, Drabek M, Yin J, Gunera J, Proll T, Kraus F, Langmead CJ, Hübner H, Weikert D, Kolb P, Rosenbaum DM, Gmeiner P. Structure-based development of a subtype-selective orexin 1 receptor antagonist. Proc Natl Acad Sci U S A. 2020;117(30):18059-67.
